# Multi‐Enzyme Nanoparticles as Efficient Pyroptosis and Immunogenic Cell Death Inducers for Cancer Immunotherapy

**DOI:** 10.1002/advs.202408729

**Published:** 2024-10-09

**Authors:** Hekai Yang, Guangzhao Xu, Fahui Li, Guanhong Guo, Ping Yan, Yuxi Chen, Yongkang Chen, Wen Sun, Weiguo Song, Wenda Zhong

**Affiliations:** ^1^ School of Pharmacy Shandong Second Medical University Weifang 261053 China; ^2^ Harway Pharma Co., Ltd. Dongying 254753 China; ^3^ State Key Laboratory of Fine Chemicals Dalian University of Technology Dalian 116024 China

**Keywords:** ER stress, immunogenic cell death, nanozyme‐cascade reaction, pyroptosis, tumor immunotherapy

## Abstract

Immunotherapy represents a widely employed modality in clinical oncology, leveraging the activation of the human immune system to target and eradicate cancer cells and tumor tissues via endogenous immune mechanisms. However, its efficacy remains constrained by inadequate immune responses within “cold” tumor microenvironment (TME). In this study, a multifunctional nanoscale pyroptosis inducer with cascade enzymatic activity (IMZF), comprising superoxide dismutase (SOD), catalase (CAT), peroxidase (POD), and glutathione oxidase (GSHO*x*), is dissociated within the acidic and glutathione‐rich TME. The vigorous enzymatic activity not only generates oxygen (O_2_) to alleviate hypoxia and promote M2 to M1 macrophage polarization but also yields reactive oxygen species (ROS) and depletes glutathione (GSH) within the TME. Functioning as an immunogenic cell death (ICD) activator and pyroptosis inducer, IMZF synergistically triggers dendritic cell maturation and inflammatory lymphocyte infiltration via ICD‐associated pyroptosis, thereby reversing immune suppression within the TMEs. Consequently, it exerts inhibitory effects on both primary and distal tumors. This cascade enzymatic platform‐based pyroptosis inducer offers an intelligent strategy for effectively overcoming immune suppression within “cold” tumors, thereby providing a promising avenue for advanced immunotherapeutic interventions.

## Graphical abstract

An ICD and pyroptosis‐induced nanoenzyme is first proposed for efficient tumor immunotherapy. The nanoenzyme IMZF demonstrates the SOD, CAT, POD, and GSHO*x*‐like catalytic ability; then thus, facilitating ROS generation, depleting GSH, and arousing ICD/pyroptosis pathways for efficient antigen presentation. This work provides a new design strategy for simultaneously enhancing antigen presentation and for reversal of immune suppression TME.

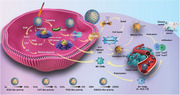

## 1 Introduction

Cancer immunotherapy has emerged as a forefront therapeutic modality, demonstrating successful applications in treating various refractory tumors and preventing tumor recurrence.^[^ ^1^ ^]^ However, the intricate, heterogeneous, and hypoxic TME, coupled with tumor‐intrinsic immune evasion, present significant challenges to conventional immunotherapeutic approaches.^[^ ^2^ ^]^ Currently, a major hurdle in this field lies in the constrained induction of sustained systemic anti‐tumor responses in “cold” tumors, characterized by inadequate immune lymphocytic infiltration and diminished immune reactivity.^[^ ^3^ ^]^ These tumor features contribute significantly to the limited efficacy in evoking enduring systemic anti‐tumor responses.^[^ ^4^ ^]^

The hallmark features of an immune‐suppressive TME include overexpression of GSH, hypoxia, low infiltration of immunogenic T cells, and inefficient antigen presentation.^[^ ^5^ ^]^ Therefore, it is conceivable to devise appropriate strategies to reprogram immunologically “cold” tumors into “hot” tumors, thereby reactivating immune responses.^[^ ^6^ ^]^ According to the previous reports, the ICD pathway and pyroptosis pathway can effectively reverse the immune‐suppressive TME to realize cancer immunotherapy.^[^ ^7^ ^]^ ICD pathway can be triggered by severe ER stress through ROS overproduction, in which the protein homeostasis of the ER itself is severely compromised.^[^ ^8^ ^]^ Numerous heat shock proteins and calcium‐binding proteins within the ER lumen are activated, translocated to the cell membrane, and serve as “eat me” signals, recruiting dendritic cells and triggering endogenous immune responses.^[^ ^9^ ^]^ Pyroptosis eliciting can also boost inflammatory responses, thereby promoting anti‐tumor immune activity.^[^ ^10^ ^]^ The substantial generation of ROS on the mitochondrial membrane disrupts its permeability, leading to membrane potential alterations, cytochrome c (Cytc) release, and other protein effluxes.^[^ ^11^ ^]^ In the presence of dATP, cytosolic Cytc binds apoptotic protease activating factor 1 (Apaf‐1), forming a complex that recruits procaspase‐9 via its caspase recruitment domain (CARD). This recruitment induces caspase‐9 activation, subsequently activating caspase‐3, which possesses potent cleavage activity.^[^ ^12^ ^]^ Activated caspase‐3 cleaves GSDME, causing its translocation to the membrane where it forms pores. This process results in cell swelling, cytoplasmic outflow, and eventual membrane rupture, characteristic of pyroptosis.^[^ ^13^ ^]^ The infiltration of inflammatory factors induced by pyroptosis enhances anti‐tumor immune responses significantly and may induce enduring immune memory.^[^ ^14^ ^]^

Thus, triggering the ICD and pyroptosis by efficient ER and mitochondria targeting ROS generators is a potential approach to realize cancer immunotherapy.^[^ ^15^ ^]^ However, the immune‐suppressive TME features (such as hypoxia and GSH overproduction) limit the efficacy of ROS generators.^[^ ^16^ ^]^ To face the challenge, researchers have engineered numerous strategies to facilitate abundant production of H_2_O_2_/O_2_ and GSH elimination, thereby promoting ROS generation.^[^ ^17^ ^]^ ZIF‐8 (Zeolitic Imidazolate Framework‐8) is a metal–organic framework (MOF) material composed of zinc ions and imidazole‐based ligands. It holds significant promise in drug delivery systems due to its notable attributes, including a high specific surface area, appropriate porosity, robust stability, and excellent biocompatibility. Further, the Mn and Fe elements incorporated with ZIF‐8 confer multifunctional nanozyme properties, enhancing its utility in diverse therapeutic applications. For instance, the IMZF can act as a SOD‐like catalyst, converting superoxide anions (O_2_^•−^) into H_2_O_2_, which is subsequently catalyzed by CAT and POD‐like catalysts to generate O_2_ and •OH.^[^ ^18^ ^]^ The GSHO*x*‐like catalyst facilitates the GSH elimination, which can guarantee the efficiency of ROS generator and promote the cancer immunotherapy.^[^ ^19^ ^]^ Although the mentioned strategies can significantly overcome the immune‐suppressive TME features, the combination of efficient ICD/pyroptosis inducer and different nano‐enzymes is still a challenge and reported limited.^[^ ^20^ ^]^ Moreover, it is necessary to study the pharmacological mechanism in the therapeutic strategy, which is also reported rarely.^[^ ^21^ ^]^

In this study, an efficient nanoenzyme‐based ICD/pyroptosis inducer named IMZF is first prepared and studied. The multifunctional nanoenzyme based on IMZF can generate H_2_O_2_, O_2_, and •OH, while consuming glutathione, thereby altering the immunosuppressive TME. The ER‐targeted photosensitizers (PSs) in IMZF can induce ER stress and trigger the release of numerous damage‐associated molecular patterns (DAMPs), thereby inducing long‐lasting ICD. In addition, mitochondria‐targeted PSs in IMZF can also arouse the active Cleaved‐caspase 3 production, cleaving the GSDME protein, and eventually leading to pyroptosis. The triggering ICD and pyroptosis pathways simultaneously promote the infiltration of many inflammatory lymphocytes. Consequently, effective immunotherapy is stimulated, and the growth of both proximal and distal tumors is suppressed. The efficacy of the ICD/pyroptosis inducer and nano‐enzymes combination strategy and the corresponding pharmacological pathway study can bring new inspiration for the development of new immunotherapy drugs.

## 2 Results and Discussion

### 2.1 Preparation, Characterization, and In Vitro ROS Generation of IMZF

**Figure 1:**
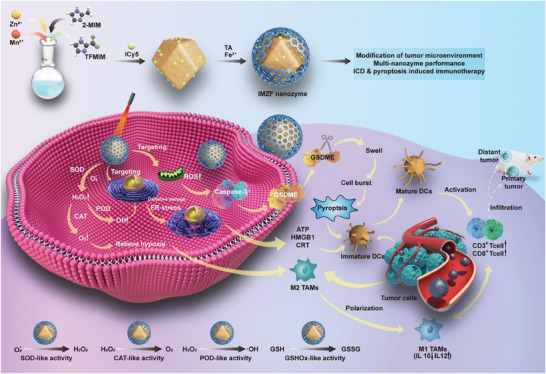
Preparation of IMZF and schematic diagram illustrating the induction of pyroptosis and ICD by IMZF for realizing immunotherapy.

**Figure** **1** provides a detailed description of the design of IMZF nanozyme. The PSs ICy5 is loaded on ZIF‐8 through capillary forces (yield the IMZ). Then, the TA and Fe^2+^ can form the pH‐responsive metal‐polyphenol network shell to yield the IMZF. When IMZF nanozyme is endocytosed by tumor cells, the outer shell will act as the SOD‐like catalyst to converse the O_2_^•−^ to be H_2_O_2_. Then, the H_2_O_2_ will be catalyzed to generate O_2_ and •OH by Zn, Fe, and Mn ions. Meanwhile, the Zn, Fe, and Mn ions will consume the GSH. Due to the reduced GSH and abundant O_2_, the PDT efficiency of ICy5 is boosted. The ER and mitochondria dual‐targeting ability of ICy5 will arouse the ICD and pyroptosis pathways by causing numerous DAMPs releases and activating caspase 3 cleaves GSDME. The infiltration of DC cells and T lymphocytes are promoted, eventually realizing anti‐cancer immunotherapy. The synthesis and characterization processes of ICy5 are detailed in Scheme S1 and Figures S1–S3, Supporting Information.

**Figure 2:**
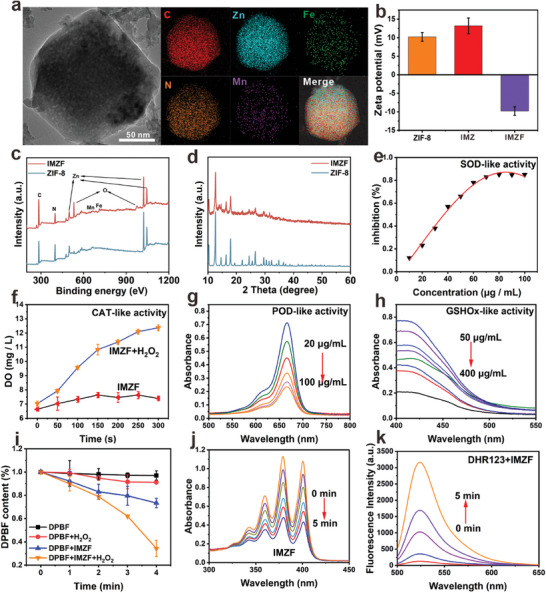
a) TEM and corresponding elemental mapping of IMZF (scar bar: 50 µm). b) Zeta potential of ZIF‐8, IMZ, and IMZF. c) XPS survey spectra of ZIF‐8 and IMZF. d) XRD survey spectra of ZIF‐8 and IMZF. e) The SOD‐like activity of IMZF. f) The CAT‐like activity of IMZF (mean ± SD, *n* = 3). g) Detection of •OH generation under different concentrations of IMZF using MB assay. h) DTNB probe caused by IMZF under acidic conditions for GSH depletion detection (pH = 6.5). i) Time‐dependent DPBF degradation curves for IMZF with or without H_2_O_2_ (10 mm) under 660 nm laser irradiation (10 mW cm^−2^) (mean ± SD, *n* = 3). j) Detection of ^1^O_2_ generation using ABDA assay under 660 nm laser irradiation (10 mW cm^−2^). k) DHR123 detection of ^1^O_2_ generation from IMZF.

TEM images display the morphology of various intermediates and the final product (Figure S4, Supporting Information). Elemental analysis confirms the containing of Mn, Fe, Zn, and N in IMZF nanoenzyme (**Figure** **2**a). The laser granulometry tests the nanoparticle size of IMZF to be 155.08 nm (Figure S5a, Supporting Information). In addition, the negative zeta potential of IMZF can be seen in Figure 2b, indicating the form of Fe─TA shell affords negative surface characteristics. After monitoring IMZF size over 7 days, the minimal size variation highlights its stable dispersion in the aqueous phase (Figure S5b, Supporting Information). Moreover, minimal size variation can also be found in different media, showing its solvent environment adaptability and dispersion stability (Figure S5c, Supporting Information). In addition, when the pH is 7.4, the zeta potential of IMZF remains relatively stable over a period of 7 days, indicating that IMZF exhibits good stability and dispersibility (Figure S5d, Supporting Information). X‐ray photoelectron spectroscopy (XPS) analysis confirms the presence of Fe elements in IMZF compared to ZIF‐8 (Figure 2c). As further shown in Figure S6a–c, Supporting Information, the valence status of Zn and Fe can be verified as Zn^2+^ and Fe^2+^.^[^ ^22^ ^]^ Due to the relatively low content of Mn, its signal peak is indistinctive. X‐ray diffraction (XRD) analysis substantiates that the form of IMZF does not change the lattice structure of ZIF‐8 (Figure 2d). Through ICP‐OES measurements, we determine the content of each element. The contents of Fe, Zn, and Mn in IMZF are 3.12%, 12.86%, and 0.91%, respectively. Thus, the existence of Fe and Zn plays the more important roles in the cascade catalytic processes. UV–vis spectroscopy further confirms the successful loading of ICy5 onto ZIF‐8, quantified at 65.4% (Figure S6d, Supporting Information).

Subsequently, the multi‐enzyme‐like activities of IMZF were evaluated. For this purpose, the Cu/Zn‐SOD and Mn‐SOD enzyme activity assays based on WST‐8 were used to assess IMZF's SOD‐like activity. WST‐8 reacted with superoxide radicals (O_2_^•−^) generated by xanthine oxidase, producing formazan with strong absorption at 450 nm. Upon addition of IMZF, UV–vis absorption intensity rapidly decreased with increasing IMZF concentration, confirming concentration‐dependent SOD‐like activity (Figure 2e). Subsequently, endogenous H_2_O_2_ in tumors was degraded to O_2_ and H_2_O via CAT‐like activity, aiding in alleviating tumor hypoxia, and potentially, reversing the tumor immune‐suppressive microenvironment. Hence, the CAT‐like activity of IMZF was evaluated using a dissolved O_2_ meter. Upon mixing IMZF (50 µg mL^−1^) with H_2_O_2_ (50 mm), an increase in O_2_ gas concentration over time was observed (Figure 2f), indicating IMZF's contribution to alleviating tumor hypoxia through CAT‐like activity, significantly enhancing photodynamic efficiency. The enriched H_2_O_2_ in tumors could be catalyzed by POD‐like enzymes to •OH, utilized in cancer CDT. Therefore, POD‐like activity was monitored using methylene blue (MB), which was degraded by •OH, resulting in a decrease in its UV absorbance peak. After mixing IMZF with H_2_O_2_ (10 mm) for 30 min, a linear relationship was observed between MB consumption and IMZF concentration, with the UV absorbance peak of MB at 665 nm progressively decreasing as the IMZF concentration increased. This indicates the generation of •OH mediated by IMZF (Figure 2g). Despite the production of abundant ROS by IMZF, the overexpression of GSH (≈10 mm) in tumors contributed to prevention of ROS damage. The GSHO*x*‐like activity of IMZF against GSH elimination was evaluated using 5,5′‐dithiobis(2‐nitrobenzoic acid) (DTNB). DTNB is a GSH indicator, reacting with GSH to produce a colored product. Addition of IMZF to a mixture of GSH and DTNB resulted in decreased UV–vis absorption intensity at 412 nm with increasing IMZF dose, confirming IMZF's potent GSHO*x*‐like activity (Figure 2h). Subsequently, under 660 nm laser irradiation (10 mW cm^−^^2^), the generation of ^1^O_2_ was monitored by the attenuation of UV–vis absorption of 1,3‐diphenylisobenzofuran (DPBF). The UV–vis–NIR intensity of the IMZF with H_2_O_2_ (10 mm) group decreased significantly under laser irradiation, which could be attributed to the abundant O_2_ through the CAT‐like performance of IMZF (Figure 2i; Figure S7, Supporting Information). Subsequently, the release of ICy5 from IMZF under different conditions was analyzed. In contrast to the slow release of ICy5 from IMZF at pH 7.4, the release rate of ICy5 was faster in acidic solutions with pH 6.5 and 5.5, which was beneficial to the controlled drug release in acidic TME (Figure S8, Supporting Information). To better demonstrate the pH‐responsive performance of IMZF nanoparticles, they were incubated in PBS buffers with pH values of 7.4, 6.5, and 5.5 after 2 h treatment. Dynamic light scattering was used to measure the hydrodynamic sizes of IMZF nanoparticles in these three different pH buffers. As the pH decreased, the size of IMZF nanoparticles also decreased (Figure S9, Supporting Information). Similarly, the morphology of IMZF observed under TEM gradually degraded (Figure S10, Supporting Information).

Following a series of experiments confirming the enzyme properties of IMZF, ICy5 was determined to have a ^1^O_2_ quantum yield of 87% using RB as a standard (Figure S11, Supporting Information). ABDA was used to detect the ability of IMZF and ICy5 to generate ^1^O_2_ under 5 min of near‐infrared (NIR) light irradiation, demonstrating IMZF's excellent ^1^O_2_ generation capability (Figure 2j; Figure S12, Supporting Information). DHR123 and SOSG probes further confirmed IMZF's excellent ROS generation ability under NIR light irradiation (Figure 2k; Figures S13–S15, Supporting Information). These findings confirm IMZF's outstanding multi‐enzyme‐like activities, including SOD‐like, CAT‐like, POD‐like, and GSHO*x*‐like enzyme activities. IMZF not only self‐supplied sufficient H_2_O_2_ and O_2_ but also generated abundant ROS and depleted GSH, suggesting potential for overcoming tumor resistance and immune‐suppressive microenvironment defences.

### 2.2 In Vitro Photodynamic Performance of IMZF

**Figure 3:**
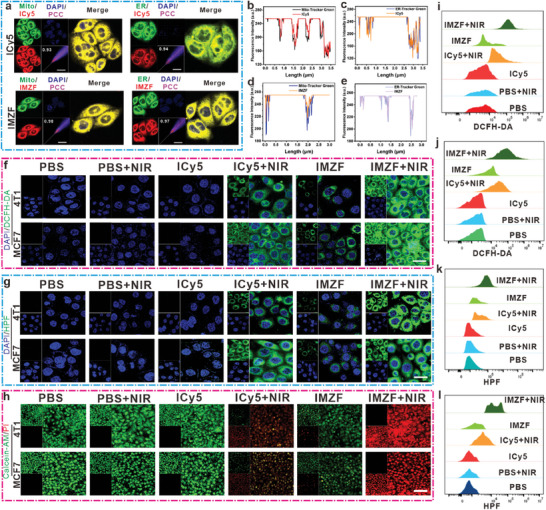
Spontaneous ER‐localizability and mito‐localizability of ICy5 and IMZF. a) Representative merged CLSM images of MCF‐7 cells treated with 0.5 µm of ER‐Tracker Green or Mito‐Tracker Green and 50 µg mL^−1^ of IMZF (red) for 2 h. For the ER/Mito‐Tracker Green, emissions were collected at 500–600 nm (*λ*_ex_ = 504 nm), For the IMZF probes, emissions were collected at 750–800 nm (*λ*_ex_ = 640 nm). Scale bar = 10 µm. b,d) Pearson correlation coefficients of the IMZF/ER‐Green‐Tracker signal overlapped in the CLSM images. c,e) Pearson correlation coefficients of the IMZF/ER‐Green‐Tracker signal overlap in the CLSM images. f) The generation of ROS in different treatment groups was assessed using DCFH‐DA respectively. For the DCFH‐DA probes, emissions were collected at 500–600 nm (*λ*_ex_ = 488 nm). Scale bar = 10 µm. g) The generation of •OH in different treatment groups was assessed using HPF, respectively. For the HPF probes, emissions were collected at 500–600 nm (*λ*_ex_ = 490 nm). Scale bar = 10 µm. h) Qualitative analysis of calcein AM and PI probe was used to evaluate the killing effect of MCF‐7 and 4T1 cells treated with IMZF under 660 nm (10 mW cm−^2^, 5 min) NIR irradiation. Scale bar = 50 µm. i,j) Indication of the fluorescence intensities of DCFH‐DA, respectively, detected using FCM in different treatment groups for two cells. k,l) Indication of the fluorescence intensities of HPF, respectively, detected using FCM in different treatment groups for two cells.

Building upon IMZF's exceptional nanozyme activity and significant ROS generation capacity in solution, we further validated its outstanding ROS production capability through cellular experiments. Initially, the cellular uptake of ICy5 and IMZF within 2 h was analyzed in MCF‐7 cells. It was observed that both IMZF and ICy5 were effectively internalized by MCF‐7 cells within this timeframe, indicating robust cellular uptake of IMZF (Figure S16, Supporting Information). According to the previous reports, the Cy5‐based owned the dual‐targeted ability to ER and mitochondria. Using a mitochondrial green fluorescent probe, the co‐localization coefficients of ICy5 and IMZF with the mitochondria were assessed following a 2‐h co‐incubation period. IMZF exhibited a co‐localization coefficient of 0.98 with mitochondria (**Figure** **3**a,d), whereas ICy5 demonstrated a coefficient of 0.93 (Figure 3a,b). This indicates the excellent mitochondrial targeting capability of the obtained compounds. Subsequently, the co‐localization coefficients of ICy5 and IMZF with the ER were measured using the similar approach. IMZF showed a co‐localization coefficient of 0.97 with the ER (Figure 3a,e), while ICy5 displayed a coefficient of 0.94 (Figure 3a,c), confirming effective targeting within the ER. Further, the co‐localization analyses were subsequently conducted on lysosomes and the Golgi apparatus, revealing relatively low co‐localization coefficients with both organelles for IMZF (Figure S17, Supporting Information). Similarly, ICy5 exhibited low co‐localization coefficients with lysosomes and the Golgi apparatus (Figure S18, Supporting Information). These experiments elucidated that the IMZF were predominant uptaken within the ER and mitochondria. To unequivocally establish the superior localization capability of IMZF nanoparticles within the ER and mitochondria in tumor cells, co‐localization analysis was performed using 4T1 cells. The overlap between the ER and mitochondrial green fluorescence probes and IMZF nanoparticles was pronounced, with co‐localization coefficients of 0.95 and 0.96, respectively (Figure S19, Supporting Information).

**Figure 4:**
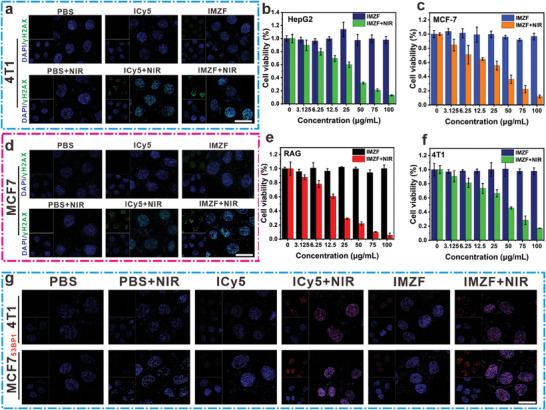
a,d) This denotes the analysis of γH2AX protein expression in 4T1 and MCF‐7 cells following various treatments using CLSM. For the γH2AX, emissions were collected at 500–600 nm (*λ*_ex_ = 488 nm). b,c,e,f) The phototoxicity of ICy5 and IMZF toward HepG2, MCF‐7, RAG, and 4T1 cells under 660 nm laser irradiation (10 mW cm^−2^, 5 min). g) This denotes the analysis of 53BP1 protein expression in 4T1 and MCF‐7 cells following various treatments using CLSM. For the 53BP1, emissions were collected at 600–700 nm (*λ*_ex_ = 561 nm). (mean ± SD, *n* = 3, *p < 0.05, **p < 0.01, ***p < 0.001, and **** *p* < 0.0001). Scale bar = 10 µm.

The generation of ROS holds paramount significance in the context of tumor cell cytotoxicity. Initially, ROS production across different treatment cohorts was evaluated utilizing the DCFH‐DA probe. Notably, irrespective of 4T1 or MCF‐7 cell lines, the IMZF + NIR group exhibited the highest ROS production (Figure 3f). Quantification of DCFH‐DA fluorescence via FCM in both 4T1 and MCF‐7 cells corroborated observations made under CLSM, demonstrating the superior ROS generation of the IMZF + NIR group (Figure 3i,j). Similarly, ROS generation was also detected in hypoxic cells treated with IMZF + NIR (Figure S20, Supporting Information). IMZF manifested notable POD‐like activity in solution, enabling the conversion of H_2_O_2_ to •OH. The •OH generation performance was assessed by using the HPF probe. Significantly intense green fluorescence, indicative of •OH presence, was observed in 4T1 and MCF‐7 cells treated with IMZF + NIR (Figure 3g). FCM analysis yielded congruent outcomes (Figure 3k,l). In addition, to further elucidate intracellular •OH generation, O27 probe was employed, revealing intense green fluorescence within the cellular milieu (Figure S21, Supporting Information). Cell viability after various treatments was assessed using Calcein‐AM and PI staining. Specifically, co‐incubation of IMZF with MCF‐7 cells, followed by NIR irradiation (660 nm, 10 mW cm^−2^, 5 min) resulted in pronounced cell death. A similar phenomenon was observed in 4T1 cells (Figure 3h). At the cellular level, it had been demonstrated that IMZF could generate cytotoxic ROS. Upon cellular uptake, IMZF induced substantial intracellular ROS production, resulting in DNA damage. The release of nuclear proteins from damaged DNA to the cell membrane surface served as a recruitment signal, attracting a large number of free dendritic cells to exert immunogenic effects. Thus, detecting nuclear damage holds significance for the activation of ICD. To delineate the dynamic process of DNA damage repair, an analysis of phosphorylated histone H2AX (γH2AX) and p53 binding protein 1 (53BP1) was conducted. 4T1 cells underwent treatment with PBS, NIR irradiation (660 nm, 10 mW cm^−2^, 5 min), and IMZF. Immunostaining with γH2AX antibody was performed on 4T1 cells under normal oxidative conditions exposed to light. Notably, compared to other groups, the cells exhibited a significant increase in nuclear density foci following treatment with IMZF + NIR. Incubation with green fluorescence secondary antibodies revealed a marked enhancement in γH2AX fluorescence intensity in the IMZF + NIR group (**Figure** **4**a); consistent results were also detected in MCF‐7 cells (Figure 4d). Further, immunostaining with red fluorescence secondary antibodies for 53BP1 showed intense red fluorescence in the IMZF + NIR group, indicating significant DNA damage (Figure 4g). Under dark conditions, cell viability exceeded 95% at concentrations of 100 µg mL^−1^ for IMZF, indicating minimal cytotoxicity of the nanohybrid. Similarly, IMZF also exhibited minimal cytotoxicity toward COS‐7 and HUVEC cells (Figure S22, Supporting Information). However, under light irradiation (660 nm, 10 mW cm^−2^, 5 min), the viability of MCF‐7 cells dramatically decreased to 11% (IMZF). Curve fitting yielded the half‐maximal inhibitory concentration (IC_50_) of IMZF in MCF‐7 cells (IC_50_ = 25.82 µg mL^−1^) (Figure 4c). Subsequently, IC_50_ values of IMZF in 4T1, RAG, and HepG2 cells were determined to be 40.12, 12.81, and 20.72 µg mL^−1^, respectively, indicating considerable phototoxicity across the four tumor cell lines (Figure 4f,e). The significantly different toxicity between normal and tumor cells indicates the biosafety and excellent cancer growth inhibitory potential of IMZF.

### 2.3 In Vitro Pyroptosis Assessment and Induction of ICD by IMZF under NIR Irradiation Conditions

**Figure 5:**
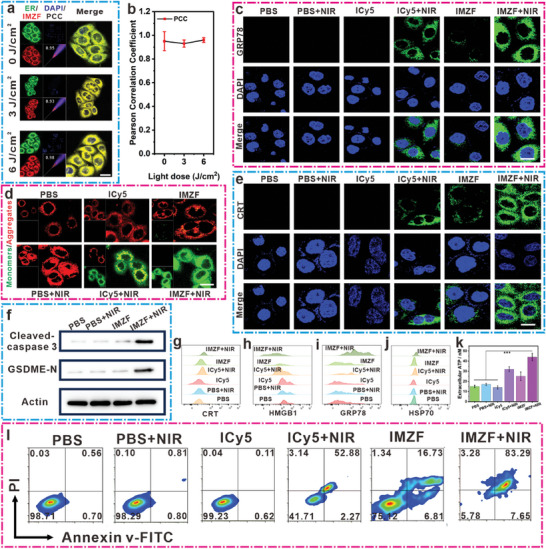
a) Conducting a co‐localization experiment in MCF‐7 cells by co‐incubating them with an ER green fluorescent probe and IMZF. For the ER‐Tracker Green, emissions were collected at 500–600 nm (*λ*_ex_ = 504 nm). For the IMZF probes, emissions were collected at 750–800 nm (*λ*_ex_ = 640 nm). Scale bar = 10 µm. b) Determining the PCC between the green and red fluorescent channels. c,e) Representation of the GRP78 and CRT immunostaining analysis of MCF‐7 cells under different treatments. For the green probes, emissions were collected at 500–600 nm (*λ*_ex_ = 488 nm). For the DAPI probes, emissions were collected at 400–500 nm (*λ*_ex_ = 350 nm). Scale bar = 10 µm. d) Detection of membrane potential in different treatment groups of cells using JC‐1. For the JC‐1 monomer probes, emissions were collected at 500–600 nm (*λ*_ex_ = 514 nm). For the JC‐1 aggregates probes, emissions were collected at 600–700 nm (*λ*_ex_ = 585 nm), For the DAPI probes, emissions were collected at 400–500 nm (*λ*_ex_ = 350 nm). Scale bar = 10 µm. f) Western blot analysis for protein expression. g–j) The variations in CRT, HMGB1, GRP78, and HSP70 were assessed via FCM utilizing markers. k) Detection of extracellular ATP content in cells treated with different treatments after NIR irradiation (mean ± SD, *n* = 3). l) FCM analysis of MCF‐7 cells treated with Annexin V‐FITC and PI (mean ± SD, *n* = 3, *p < 0.05, **p < 0.01, ***p < 0.001, and **** *p* < 0.0001).

When ICD inducers provoke ER stress, it leads to the translocation of a substantial amount of proteins, such as CRT, HSP70, and HMGB1 proteins, to the cell membrane surface.^[^ ^23^ ^]^ Serving as an “eat‐me” signal, this prompts a significant influx of dendritic cells and activates the host's innate immune response. Initially, if co‐incubating the ER‐targeting green fluorescent probe with IMZF in MCF‐7 cells, followed by exposure to varying intensities of NIR irradiation, IMZF consistently localizes well within the ER (**Figure** **5**a,b). This observation underscores IMZF's capacity for effective and stable ER targeting performance. Subsequently, the markers associated with ICD were examined. ROS generation by IMZF within the ER led to ER stress, where mild ER stress could be alleviated by chaperone proteins to restore ER function, while excessive ROS production induced severe ER stress, resulting in the release of a plethora of DAMPs, including GRP78, HMGB1, and CRT. Co‐incubation of IMZF with MCF‐7 cells, followed by exposure to NIR light (10 mW cm−^2^, 5 min) revealed a significant increase in GRP78 protein expression (Figure 5c,i). The translocation of CRT protein from the ER lumen to the cell membrane surface was observed, indicative of ER stress (Figure 5e,g). The HMGB1 protein significantly translocated to the extracellular fluid in the IMZF + NIR treatment group (Figure 5h; Figure S23, Supporting Information). Concurrently, a substantial activation of HSP70 was detected, with subsequent extracellular translocation, further indicating ICD induction (Figure 5j; Figure S24, Supporting Information). A key hallmark of ICD is the extracellular release of ATP and LDH. The ATP efflux after different treatments was evaluated, showing that IMZF + NIR treatment induced the highest level of ATP and LDH efflux compared to the PBS control and IMZF alone group (Figure 5k; Figure S25, Supporting Information). Overall, these findings suggest that IMZF, through targeted ER localization and subsequent ROS generation, induced ER stress and triggered ICD, promoting the release of DAMPs and ATP efflux, ultimately enhancing the immune response against tumors. To further elucidate the superior ability of IMZF nanoparticles to induce ICD, hypericin was used as a positive control. The results indicate that following treatment with IMZF nanoparticles and subsequent NIR light (660 nm, 10 mW cm^−^^2^, 5 min) irradiation, CRT activation was significantly pronounced, whereas hypericin only elicited minimal green fluorescence (Figure S26, Supporting Information). Based on its ability to effectively target mitochondria, IMZF further induced alterations in mitochondrial membrane permeability (MMP). Typically, changes in MMP led to the mixing of intra‐ and extramitochondrial components, along with the release of cytochrome c. Bcl‐2 protein, an anti‐apoptotic factor, experienced decreased expression, thereby promoting pyroptosis, whereas Bax protein, a pro‐apoptotic factor, exhibited increased expression, further facilitating pyroptosis. With enhanced MMP, cytochrome c was released from the mitochondrial intermembrane space into the cytoplasm. Subsequently, cytochrome c formed a complex with Apaf‐1 in the cytoplasm, thereby activating caspase 3, which underwent cleavage to its active form, Cleaved‐caspase 3. Notably, our observations revealed elevated expression of Bax protein and reduced expression of Bcl‐2 protein in the IMZF combined with NIR treatment group, accompanied by heightened cytoplasmic expression of Apaf‐1 (Figures S27–S29, Supporting Information). In addition, previous research supported Cleaved‐caspase 3 as a proficient protease capable of cleaving various intracellular proteins. The Gasdermin protein family had been identified as crucial execution molecules in inducing pyroptosis. Moreover, distinguishing the N‐terminal domain of GSDME is paramount. Upon cleavage by activated caspase 3, GSDME generated the GSDME‐N, responsible for membrane permeabilization and pyroptosis induction.^[^ ^11^, ^24^ ^]^ Employing western blotting and CLSM, the levels of GSDME N and Cleaved caspase‐3 in MCF‐7 cells subjected to different formulations were assessed, thereby elucidating their pyroptotic mechanisms. Results demonstrated significant caspase 3‐mediated cleavage and activation of GSDME in the IMZF combined with NIR treatment group compared to other treatment groups (Figure 5f; Figures S30 and S31, Supporting Information). Subsequently, we assessed alterations in mitochondrial membrane potential using JC‐1 staining post‐treatment across different groups. It was observed that the IMZF, combined with the NIR treatment group, exhibited prominent green fluorescence compared to the PBS group, indicative of mitochondrial membrane potential reduction. This corroborates our hypothesis regarding MMP triggering pyroptosis (Figure 5d; Figure S32, Supporting Information). FCM was employed in this study to quantitatively assess cellular pyroptosis. Results demonstrated that ICy5 treatment alone induced minimal cellular pyroptosis. However, co‐incubation of ICy5 and IMZF with MCF‐7 cells, followed by subsequent NIR irradiation (660 nm, 10 mW cm^−^^2^, 5 min), significantly induced cellular pyroptosis, with levels reaching 52.88% and 83.29%, respectively (Figure 5l; Figure S33, Supporting Information). This experiment conclusively established the capability of IMZF to induce ICD and activate cellular pyroptosis.

### 2.4 NIR‐Mediated IMZF In Vitro and In Vivo Pyroptosis Combined with ICD Induction Promotes Antitumor Immunity

**Figure 6:**
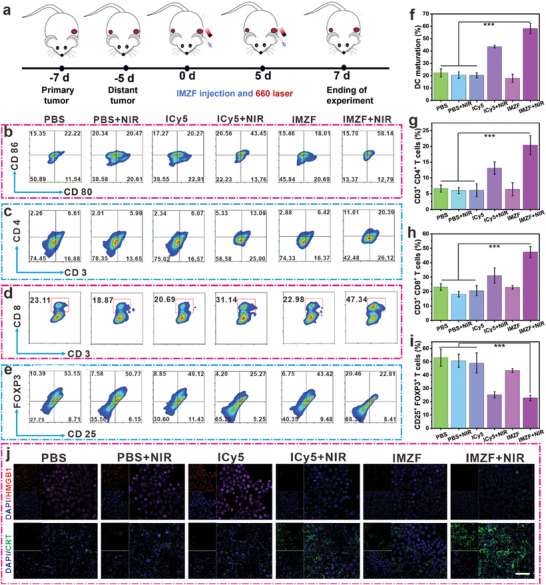
a) Immune model construction and research process. b–e) Representation of the expression of DC cells, CD4^+^ CTL cells, CD8^+^ CTL cells, and Treg cells detected using FCM methodology. The quantitative analysis of f) DC cells, g) CD4^+^ CTL cells, h) CD8^+^ CTL cells, and i) Treg cells. j) Immunofluorescence staining of HMGB1 and CRT of tumor tissues in different experimental groups. DAPI was used to stain the cell nucleus (mean ± SD, *n* = 3. *p < 0.05, **p < 0.01, ***p < 0.001 and **** *p* < 0.0001). Scale bar: 50 µm.

A pivotal factor in the immunosuppressive TME is the infiltration of M2 macrophages into “cold” tumors.^[^ ^25^ ^]^ A promising strategy in cancer immunotherapy involves transitioning macrophages from an M2 phenotype to an M1 phenotype to counter immune suppression. Previous research has indicated that heightened ROS production can induce oxidative stress.^[^ ^26^ ^]^ Motivated by the ROS‐generating capacity of IMZF, we conducted immune activation experiments using 4T1 cells. RAW264.7 macrophages were exposed to IL‐4 to establish an M2 macrophage population, confirming the polarization of tumor‐associated macrophages (TAMs) induced by IMZF. Co‐culturing RAW264.7 macrophages with differently treated 4T1 cells in a Transwell insert system enabled the analysis of M2 and M1 macrophage proportions via FCM. Compared to the control group, the proportions of M2 macrophages in the ICy5 + NIR and IMZF + NIR groups significantly decreased from 43.16% to 34.45% and 19.02%, respectively, while the proportions of M1 macrophages increased from 20.13% to 39.80% and 57.41%, respectively (Figures S34 and S35, Supporting Information). The levels of IL‐10 secreted by M2 macrophages and IL‐12 secreted by M1 macrophages were quantified using ELISA kits. Similarly, the trends in IL‐10 and IL‐12 levels in the ICy5 + NIR and IMZF + NIR groups mirrored those of M2 and M1 macrophages (Figure S36, Supporting Information). These findings suggest that IMZF effectively promotes the differentiation of M2 macrophages into M1 macrophages, thereby reversing immune suppression within the TME. Benefiting from promising outcomes achieved in ex vivo immune activation, we sought to explore the potential anti‐tumor immune response of IMZF. As illustrated in **Figure** **6**a, a bilateral 4T1 tumor model was established in mice, with primary and distant tumors engineered on each side, corresponding to days 7 and 5 prior to treatment initiation, respectively. IMZF was administered followed by 660 nm laser irradiation (10 mW cm^−2^, 5 min) of the primary tumor 4 h later. Subsequently, mice underwent additional treatments on day 5. Tumors from different treatment groups were dissociated into single‐cell suspensions and subjected to immune cell analysis using various antibody stains. In Figures S37 and S38, Supporting Information, a significant reduction in M2 macrophages was observed in the primary tumor group treated with IMZF + NIR compared to the PBS group (from 46.27% to 17.49%), while M1 macrophages significantly increased in the IMZF + NIR treated primary tumor group (from 16.45% to 46.92%). The observed shift in polarization toward M1 macrophages indicates that IMZF + NIR effectively reshaped the immunosuppressive TME into an immune‐supportive state. Subsequently, the expression of immune molecules in both primary and distant tumors in mice was evaluated. Compared to the PBS group, the ICy5 + NIR group exhibited a dendritic cell maturation rate of 43.45% at the primary tumor site (Figure 6b,f; Figure S39, Supporting Information). In comparison, the dendritic cell maturation rate increased to 58.14% with IMZF + NIR treatment (660 nm, 20 mW cm^−2^, 5 min). Similar trends were observed when assessing distant tumors (Figure S40, Supporting Information). CTLs play a crucial role in anti‐tumor immunity. In the primary tumors of the IMZF + NIR group, a significant increase in the proportion of CD3^+^ CD4^+^ T cells (from 6.61% to 20.39%) (Figure 6c,g; Figure S41, Supporting Information) and CD3^+^ CD8^+^ T cells (from 23.11% to 47.34%) (Figure 6d,h) was observed compared to the PBS group. Similar results were obtained when evaluating distant tumors (Figures S42 and S43, Supporting Information). Treg cells, a subset of immunosuppressive T cells, served as a marker for the host's immunosuppressive status. In the primary tumors of the IMZF + NIR group, a significant decrease in the proportion of CD25^+^ FOXP3^+^ T cells (from 53.15% to 22.82%) was observed compared to the PBS group (Figure 6e,i; Figure S44, Supporting Information). In addition, a significant decrease in the proportion of CD25^+^ FOXP3^+^ T cells was observed in distant tumors (from 47.03% to 14.18%) (Figure S45, Supporting Information). These findings collectively underscore the potent immunomodulatory effects of IMZF under NIR irradiation, thereby ameliorating the immunosuppressive TME. Further, IMZF not only triggered ICD but also induced cellular pyroptosis. The combined action of both led to substantial infiltration of inflammatory cells. This, in turn, not only increased the proportion of cytotoxic T cells but also diminished the population of suppressive T cells, thereby promoting anti‐tumor immunity. This robust immunostimulatory response correlated with the extent of ICD induced by the combination of PDT and CDT. Immunohistochemical analysis of tissue samples from various treatment groups confirmed a significant upregulation of CRT expression and the release of HMGB1 into the bloodstream in tumors treated with IMZF + NIR. These results are consistent with earlier cellular experiments (Figure 6j), highlighting the potential of this combination therapy in eliciting a potent anti‐tumor immune response.

### 2.5 Immunotherapy Through Induction of ICD and Pyroptosis

**Figure 7:**
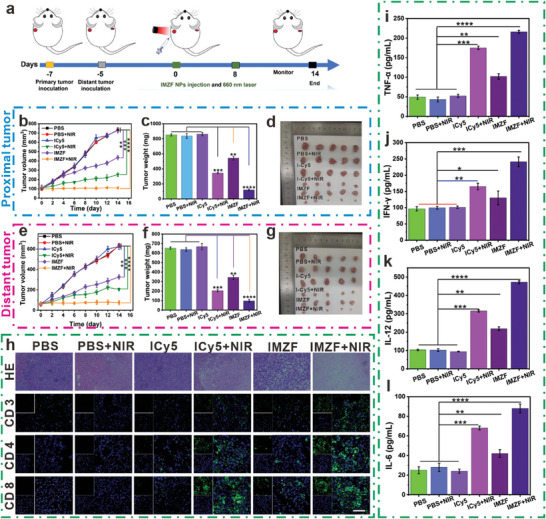
a) Mouse model generation process diagram. b) The volume of proximal tumors in mice (mean ± SD, *n* = 5). c) The weight of proximal tumors in mice (mean ± SD, *n* = 5). d) Images of proximal tumors in mice. e) The volume of distal tumors in mice (mean ± SD, *n* = 5). f) The weight of distal tumors in mice (mean ± SD, *n* = 5). g) Images of proximal tumors in mice. h) Immunofluorescent staining of tumor tissues in mice, including CD3 molecule, CD4 molecule, and CD8 molecule. i–l) The concentration of relative cytokines (TNF‐𝛼, IFN‐𝛾, IL‐12, and IL‐6) of serum post various treatments (mean ± SD, *n* = 3, and *p < 0.05, **p < 0.01, ***p < 0.001 and ****p < 0.0001). Scale bar = 50 µm.

The anti‐tumor efficacy of IMZF was evaluated in mice bearing bilateral 4T1 tumors, showing its excellent ROS generation and inducing lymphocyte infiltration through necrosis (Figure S46, Supporting Information). To evaluate the in vivo feasibility of IMZF, hemolysis assays were conducted. At a concentration of 1.0 mg mL^−1^, the hemolysis rate of IMZF was less than 5% (Figure S47, Supporting Information), indicating that the nanohybrid exhibited a low risk of hemolysis and was thus safe for in vivo applications. Subsequently, fluorescence imaging (FLI) was employed to monitor the distribution of IMZF in 4T1 tumor‐bearing BALB/c mice. FLI results demonstrated an increased accumulation of IMZF in the tumor region, peaking at 4 h post‐injection, and subsequently decreasing over time. Semi‐quantitative ex vivo imaging of the heart, liver, spleen, lungs, kidneys, and tumor at 36 h post‐injection revealed that the IMZF predominantly accumulated in the tumor tissue (Figure S48, Supporting Information). The antigenicity of cell death induced solely by PDT was typically insufficient; while, IMZF served as a multifunctional inducing agent, promoting extensive lymphocyte infiltration at the tumor site by ROS production and TME regulation. Mice were randomly divided into six groups (*n* = 5): PBS group, PBS + NIR group, ICy5 group, ICy5 + NIR group, IMZF group, and IMZF + NIR group. The treatment regimen for mice is depicted in **Figure** **7**a. In the PBS group, both primary and distant tumors exhibited rapid growth. After 14 days of treatment, the average volumes increased from 97 to 734 mm^3^ (7.56‐fold increase) and from 85 to 632 mm^3^ (7.43‐fold increase), respectively (Figure 6b,e). Compared to the PBS group, the IMZF + NIR group demonstrated significant inhibitory effects. After 14 days, the volume of the primary tumor remained almost unchanged, while the distant tumor increased from 62 to 85 mm^3^ (1.37‐fold increase), indicating a significant level of tumor suppression (Figure 7b,e). In these groups, mice in the IMZF + NIR group exhibited the smallest tumor mass (Figure 7c,d,f,g). Across all groups, there were no significant changes in average body weight, indicating the high safety and low toxicity of our materials (Figure S49, Supporting Information). Histological analysis with H&E staining of the heart, liver, spleen, lungs, and kidneys of each group of mice revealed no apparent organ damage in the IMZF + NIR group compared to the PBS group, demonstrating the absence of drug‐induced organ toxicity (Figure S50, Supporting Information). Examination of tumor tissues across all groups revealed dense tumor tissue without significant damage in the PBS group. However, pronounced tissue damage and sparse tissue distribution were observed in the ICy5 + NIR group. Similarly, the IMZF + NIR group exhibited noticeable tissue damage characterized by nuclear condensation and sparse tissue distribution (Figure 7h). The extent of the immune response to tumors correlates directly with the quantities of CTLs and helper T cells. Considering that the immune response to distant tumors reflects both primary tumor and systemic immune activation, we employed immunofluorescence staining to assess the infiltration of CD3^+^ T cells, CD4^+^ T cells, CD8^+^ T cells, and Treg cells in distant tumors. Immunofluorescence detection of CD3^+^ T cells, CD4^+^ T cells, and CD8^+^ T cells revealed significantly stronger fluorescence in both the ICy5 + NIR and IMZF + NIR groups compared to the PBS group (Figure 7h), indicating abundant infiltration of CD3^+^ T cells, CD4^+^ T cells, and CD8^+^ T cells into the tumors. The immunological memory of immune cells enabled rapid immune responses upon re‐encountering with antigens. Consequently, we measured relevant parameters. Central memory T cells (Tcm) predominantly resided in lymphoid tissues and likely provided protective effects through processes such as amplification, differentiation, and migration following antigenic stimulation. By secreting various cytokines such as TNF‐α and IFN‐γ, they offered timely immune protection upon secondary encounter with the same pathogen. Following IMZF and NIR treatment, a significant production of IFN‐γ and TNF‐α cytokines was observed in the serum (Figure 7i,j). Surprisingly, levels of IL‐6 and IL‐12 showed similar outcomes (Figure 7k,l). These findings suggest that IMZF can serve as an immunomodulatory agent stimulating T cell infiltration and activation in situ, thereby eliciting persistent and effective immune memory for CTL‐mediated immunity.

Given the prominent ability of IMZF to induce antitumor immune responses and reverse the immunosuppressive TME, we investigated the effects of combining IMZF with αPD‐1 on tumor growth and immune activation. First, a subcutaneous tumor model in mice was established and treated according to the protocol depicted in Figure S51a, Supporting Information with PBS (control), PBS + NIR, IMZF, αPD‐1, IMZF + NIR, and IMZF + NIR + αPD‐1. The growth curves of body weight, average tumor volume, and tumor weight changes are shown in Figures S51b–d, Supporting Information, respectively. Clearly, the control group exhibited the fastest tumor growth. In contrast, the αPD‐1 group showed a certain degree of inhibition of primary tumor growth. Further, the combination of IMZF + NIR + αPD‐1 significantly suppressed tumor growth. Immunofluorescence staining also confirmed that the IMZF + NIR + αPD‐1 combination treatment enhanced the infiltration of CD3^+^ T cells and CD4^+^ T cells (Figure S51e, Supporting Information). These results indicate that the IMZF, combined with αPD‐1 under NIR light stimulation, possessed potent antitumor efficacy. Throughout the treatment period, there were no significant differences in body weight among the groups. Evaluation of major organs via H&E staining did not reveal any noticeable tissue lesions or inflammation post‐treatment (Figure S52, Supporting Information). In addition, blood routine and biochemical analyses showed no significant differences between groups (Figures S53 and S54, Supporting Information), indicating good biocompatibility.

## 3 Conclusion

The in vitro and in vivo experiments confirmed the efficient antigen presentation and successful realization of cancer immunotherapy by IMZF. The multifunctional nanoenzyme performance of IMZF promoted the H_2_O_2_, O_2_, and •OH generation, while consuming glutathione, thereby altering the immunosuppressive TME. Due to the severe ER stress and cleaving GSDME protein, the arousing ICD and pyroptosis pathway facilitated the M2/M1 macrophage polarization, DC maturation, and CTL infiltration. Ultimately, the systemic immune responses were activated, which inhibited the growth of both proximal and distal cancer. This research presents a promising strategy to simultaneously reverse the immunosuppressive TME, inducing ICD and pyroptosis pathways, which eventually realize efficient cancer immunotherapy.

## 4 Experimental Section

### The SOD‐Like Activity of IMZF

The SOD‐like activity was evaluated utilizing the Cu/Zn‐SOD and Mn‐SOD Assay Kit with WST‐8. In short, WST‐8 reacted with O_2_^•−^ generated by xanthine oxidase, producing a vividly colored formazan with robust UV–vis absorbance at 450 nm. Upon exposure to nanomaterials exhibiting SOD‐like enzyme activity, O_2_^•−^ was converted into water and O_2_, hindering formazan production. Specifically, IMZF at various concentrations was incubated with WST and xanthine oxidase for 30 min at 37 °C. Subsequently, the UV–vis absorbance at 450 nm was measured. The percentage inhibition was calculated using the formula: inhibition (%) = [(*A*_0_ *− A*_i_)/*A*_0_] × 100%, where *A*_0_ and *A*_i_ represented the absorbances of the control and sample, respectively.

### •OH Generation Resulting from the POD‐Like Activity of IMZF

In the standard procedure, MB (5 µg mL^−1^), H_2_O_2_ (10 mm), and IMZF (100 µg mL^−1^) were combined in a NaHCO_3_ (25 mm) solution and incubated at 37 °C for 30 min. The degradation of MB induced by •OH was evaluated by monitoring absorbance changes at 665 nm. The concentration‐dependent POD‐like activity of IMZF was assessed using UV–vis–NIR.

### The Impact of IMZF's CAT‐Like Activity on the Generation Capability of O_2_

At 37 °C, O_2_ production was evaluated using a dissolved O_2_ meter to monitor the catalase‐mimicking activity of IMZF. A volume of 50 µL of H_2_O_2_ (2 m) was added to 2 mL of IMZF solution (50 µg mL^−1^). The concentration of dissolved O_2_ (mg L^−1^) was measured at different reaction time points.

### 1O_2_ Generation Ability of IMZF

The generation of ^1^O_2_ in IMZF solution under varying durations of irradiation was examined using 1,3‐diphenylisobenzofuran (DPBF) as a detection probe for ^1^O_2_. Specifically, IMZF samples, with or without H_2_O_2_ (1 mm), were dispersed in a 5 mL aqueous solution containing DPBF. The mixture underwent irradiation with a 660 nm laser at a power density of 10 mW cm^−2^ for different time periods. Subsequently, the production of ^1^O_2_ was evaluated using UV–vis spectrophotometry.

### Cell Culture

MCF7 cells, 4T1 cells, RAG cells, and HepG2 cells were cultured in DMEM medium supplemented with 10% fetal bovine serum and 1% antibiotic (penicillin/streptomycin, 100 U mL^−1^). The cells were maintained in a CO_2_/air atmosphere (5%/95%) at 37 °C.

### Measurement of Mitochondrial Membrane Potential

With respect to assessment of mitochondrial membrane potential using JC‐1 staining and confocal imaging, initially, the cells were seeded in glass‐bottom cell culture dishes and cultured in a medium containing IMZF (50 µg mL^−1^). After 4 h of incubation, the cells were stained with JC‐1 according to the protocol. Following three washes with incomplete medium, the cells were immediately subjected to confocal imaging upon irradiation with a 660 nm laser (10 mW cm^−2^, 5 min).

### Cytotoxicity Test

4T1 cells were plated in a 96‐well plate at a density of 1 × 10^4^ cells per well in 100 µL of medium and cultured at 37 °C for 24 h. After removing the original medium, varying concentrations (0–100 µg mL^−1^) of IMZF were introduced into the wells. The cells were then further cultured for 2 h in a constant temperature environment. Subsequently, the experiment was conducted under 660 nm light (10 mW cm^−2^, 5 min), followed by continued incubation for 24 h. Afterward, 20 µL of MTT solution (5 mg mL^−1^) was added to each well, and the cells were incubated for an additional 4 h. The old medium was aspirated, and 150 µL of dimethyl sulfoxide was added to each well. After oscillation for 10 min, absorbance at 570 nm was measured using a microplate reader (SpectraMax i3×) to assess cell viability via the provided formula.

(1) $$\mathrm{Cell}\mathrm{viability}(\%)=\frac{OD_{\mathrm{PS}}-OD_{\mathrm{black}\;\mathrm{control}}}{OD_{\mathrm{control}}-OD_{\mathrm{black}\;\mathrm{control}}}\times 100\%$$

For the determination of the darkness of the IMZF, cancelling the lighting conditions and other steps were the same. The 4T1 cells were measured in the same way.

### Cell Uptake Test

The cellular internalization of IMZF was assessed at multiple time intervals. Cells were seeded onto laser confocal culture dishes with 1 mL of medium, resulting in a cell density of 1 × 10^5^ per mL, and incubated for 24 h in a constant‐temperature environment. Following this, they were exposed to IMZF at a concentration of 50 µg mL^−1^ at varying time points. Subsequent to treatment, the cells underwent triple washing with PBS and were then examined using CLSM to visualize the internalization process.

### Detection of Intracellular ^1^O_2_

Individual cells were seeded onto laser confocal culture dishes to achieve a density of 1 × 10^5^ cells per mL. Subsequently, the culture dishes were incubated in a cell culture incubator for 24 h until the cells adhered completely. The seeded dishes were then divided into six experimental groups: PBS group, PBS + NIR group, I‐Cy5 group, I‐Cy5 + NIR group, IMZF group, and IMZF + NIR group. Following a 1‐h incubation period in the cell culture incubator, green fluorescent probes DCFH‐DA (10 µm) were introduced, and the cells were further incubated for an additional hour. The PBS + NIR group, I‐Cy5 + NIR group, and IMZF + NIR group were subjected to NIR light exposure (660 nm, 10 mW cm^−2^) for 5 min. Subsequent to light exposure, the old culture medium was removed, and the cells were washed three times with PBS. Observations were then conducted using CLSM.

### Subcellular Localization

The cells were aliquoted into laser confocal culture dishes and incubated in a constant‐temperature incubator for 24 h. Subsequently, a fresh culture medium containing IMZF (50 µg mL^−1^) was added, and the cells were further incubated for 2 h. After incubation, the purchased subcellular localization probe was added to the laser confocal culture dish according to the instructions, and the cells were incubated for an additional 1 h. The cells were washed three times with PBS, a fresh culture medium was added, and observations were made under a CLSM.

### Apoptosis Detection

Cells extracted from a 25T culture flask were seeded onto a 6‐well plate at a density of 1 × 10^5^ cells per mL. Subsequently, the plate was placed into a cell culture incubator and incubated for 24 h until the cells had adhered completely. According to the experimental protocol, the cells were divided into six groups: PBS group, PBS + NIR group, I‐Cy5 group, I‐Cy5 + NIR group, IMZF group, and IMZF + NIR group. I‐Cy5 and IMZF (50 µg mL^−1^) were added accordingly, and the incubation continued in the cell culture incubator for an additional 2 h. Subsequently, the PBS + NIR group, I‐Cy5 + NIR group, and IMZF + NIR group were exposed to NIR light at 660 nm and 10 mW cm^−2^ for 5 min. Following light exposure, the cells were further incubated in the cell culture incubator for 12 h. Enzymatic digestion was performed, followed by the addition of appropriate detection reagents, and the samples were subjected to machine testing.

### Expression of the GRP78 Protein

The cells in the 25T flask were individually cultured in laser confocal culture dishes at a density of 1 × 10^6^ cells per mL. Afterward, the dishes were placed in a cell culture incubator for 24 h until the cells had fully adhered to the surface. As per the experimental requirements, the cells were divided into six groups: PBS group, PBS + NIR group, I‐Cy5 group, I‐Cy5 + NIR group, IMZF group, and IMZF + NIR group. Following treatment with IMZF and I‐Cy5 (50 µg mL^−1^), the cells were further incubated in the incubator for an additional 2 h and then exposed to NIR light (660 nm, 10 mW cm^−2^) for 5 min. After light exposure, the old culture medium was aspirated, and the cells were fixed overnight with an immunostaining fixative solution. Subsequent to fixation, the cells were incubated with GRP78 primary antibody (1:100) for 4 h. Then, the primary antibody was removed, and the culture dishes were washed with PBS before being incubated with fluorescent secondary antibody for an additional 4 h. Following the incubation, the secondary antibody (1:500) was removed, and the dishes were washed with PBS. The samples were then observed under CLSM.

### Establishment of Bilateral Tumor Model in Mice

All procedures adhered to the Guidelines for the Protection and Utilization of Laboratory Animal Resources and were approved by the Laboratory Animal Ethics Committee of Shandong Second Medical University, under ethics approval number 2022SDL381. Six‐week‐old Balb/c mice were procured from Beijing Vital River Laboratory Animal Technology Co., Ltd. Initially, the mice were acclimatized to their new environment for 1 week. Subsequently, tumors were implanted in the left axillary region. Upon reaching the third day of left‐side tumor growth, tumors were implanted on the right side using the same method. Mice were divided into six groups as per experimental requirements: PBS group, PBS + NIR group, I‐Cy5 group, I‐Cy5 + NIR group, IMZF group, and IMZF + NIR group. Treatment was directed at the left‐side tumor. When the volume of the left‐side tumor approached 100 mm^3^, mice underwent intratumoral injection of IMZF (1 mg kg^−1^) and I‐Cy5. Four hours post‐injection, the mice were subjected to near‐infrared (660 nm, 20 mW cm^−^^2^) irradiation. After treatment, the weights and tumor volumes on both sides of the mice were recorded, with daily observations. The formula for calculating mouse tumor volume was as follows:

(2) $$V=\frac{a\times b^{2}}{2}$$

*V* represents the tumor volume, and *a* and *b* represent the long diameter and short diameter, respectively.

### Detection of Surface Molecular Expression in Mouse Tumors

After murine tumor cells were extracted, the tumor tissues were finely minced using surgical scissors. Subsequently, the minced tissues underwent a 30‐min digestion period in collagenase at 37 °C. Following digestion, the cells were suspended in PBS and filtered through a 300‐mesh filter membrane. The resulting cell suspension was centrifuged at 1500 rpm for 5 min. The pellet was then initially suspended in buffer, with 600 µL of buffer added to each tube. Subsequently, 30 µL aliquots were taken for staining, and staining procedures were conducted for 1 h before testing on the machine.

### Statistical Analysis

All in vitro experiments were performed at least three times. All in vivo studies were conducted after randomization into groups. The data were presented as mean ± standard deviation (S.D.) with the indicated sample size in figure legends. One‐way single factorial analysis of variance (ANOVA) was carried out to ascertain the statistical difference of the data. GraphPad Prism 10.1.2 was used for data statistics and statistical significance calculation. *P* < 0.05 was taken as statistically significant (*P < 0.05, **P < 0.01, ***P < 0.001, and ****P < 0.0001).

## Conflict of Interest

The authors declare no conflict of interest.

## Supporting information



Supporting Information

## Data Availability

The data that support the findings of this study are available from the corresponding author upon reasonable request.
