# Supplementary material for: Multi‐Enzyme Nanoparticles as Efficient Pyroptosis and Immunogenic Cell Death Inducers for Cancer Immunotherapy
Source: Adv Sci (Weinh). 2024 Oct 9;11(44):2408729. doi: 10.1002/advs.202408729 (PMC11600289; doi:10.1002/advs.202408729)
Supplement: Supplementary file 1 — Supporting Information [file ADVS-11-2408729-s001.docx]

**Multi-Enzyme Nanoparticles as Efficient Pyroptosis and Immunogenic Cell Death Inducers for Cancer Immunotherapy**

*Hekai Yang^1,#^, Guangzhao Xu^1,2,#^, Fahui Li^1,#^, Guanhong Guo^1^, Ping Yan ^1^, Yuxi Chen^1^, Yongkang Chen^1^, Wen Sun^3^, Weiguo Song^1,^*, Wenda Zhong^1,^**

*^1^ H. K. Yang, G. Z. Xu, Prof. F. H. Li, G. H. Guo, P. Yan, Y. X. Chen, Y. K. Cheng, Prof. W. G. Song, W. D. Zhong.*

*School of Pharmacy,* *Shandong Second Medical University, Weifang 261053, China*

*^2^ G. Z. Xu*

*Harway Pharma Co., Ltd. Dongying 254753, China*

*^3^ Prof. W. Sun*

*State Key Laboratory of Fine Chemicals, Dalian University of Technology, Dalian 116024, China*

*^#^These authors contributed equally to this work.*

**Corresponding authors.*

*E-mail: songwg@sdsmu.edu.cn; zhongwd@sdsmu.edu.cn.*

**Materials**

Zn(OAc)_2_·2H_2_O, manganese acetylacetonate, 2-methylimidazole (2-MIM), 4-(trssifluoromethyl)-1H-imidazole (TFMIM), methylene blue (MB), and ammonia solution (NH_3_·H_2_O) were sourced from Shanghai Aladdin Biochemical Technology Co., Ltd. Cu/Zn-SOD and Mn-SOD Assay Kit with WST-8 were procured from Beyotime Biotechnology (S0103). 5,5'-Dithiobis (2-nitrobenzoic acid) (DTNB), 1,3-Diphenylisobenzofuran (DPBF) were obtained from Bide Pharmatech Ltd (Shanghai, China). All solvents utilized were of analytical grade. Rose bengal (RB) and 9,10-Anthracenediyl-bis(methylene)dimalonic Acid (ABDA) were acquired from Shanghai Aladdin Biochemical Technology Co., Ltd. ER-Tracker Green, Lyso-Tracker Green, Golgi-Tracker Green, Hoechst 33342, Mitochondrial Membrane Potential Assay Kit with JC-1, Annexin V-FITC Apoptosis Detection Kit, and Cytotoxicity Assay Kit were purchased from Beyotime Biotechnology Co., Ltd. The Mouse IL-10 ELISA kit, Mouse IL-12, TNF- α ELISA kit and IFN-γ were procured from Shanghai Solarbio Bioscience & Technology Co., Ltd. PE Anti-Mouse CD86 Antibody[GL-1], FITC Anti-Mouse CD80 Antibody[16-10A1], FITC Anti-Mouse CD3 Antibody[17A2], PE Anti-Mouse CD8 Antibody[53-6.7], PE Anti-Mouse CD4 Antibody[RM4-5], FITC Anti-Mouse CD25 Antibody[PC-61.5.3], PE Anti-Mouse Foxp3 Antibody[3G3] and FITC Anti-Mouse CD206/MMR Antibody[C068C2] were obtained from Proteintech Biotechnology Co., Ltd. The ATP assay kit and lactate dehydrogenase assay kit were purchased from Beyotime Biotechnology Co., Ltd. MTT (3-(4,5-dimethyl-2-thiazolyl)-2,5-diphenyl-2-Htetrazolium bromide) was purchased from Energy Chemical Co., Ltd. Hydroxyphenyl fluorescein (HPF) was purchased from Shanghai Maokang Biotechnology Co., Ltd. Calcein-AM/PI Detection Kit, GSH and GSSG Assay Kit, GPX4 Rabbit Polyclonal Antibody, HRP-labeled Goat Anti-Rabbit IgG (H+L) were purchased from Beyotime Biotechnology Co., Ltd. C11 BODIPY 581/591 Lipid Peroxidation Sensor was purchased from Mao Kang Biotechnology Co., Ltd. (Shanghai, China). Michigan Cancer Foundation-7 (MCF-7) were purchased from Institute of Basic Medical Sciences (IBMS) of the Chinese Academy of Medical Sciences. Anti-cleaved N-terminal GSDME [EPR20867-248] were purchased from abcam. Tannins (TA) and FeCl_2_·4H_2_O were purchased from Bide Pharmatech Co., Ltd.

**Methods**

^1^H-NMR and ^13^C-NMR spectra were measured using a Bruker Avance II 400 spectrometer. Mass spectrometric (ESI-MS) data were detected utilizing an Ultimate 3000 (Thermo Scientific) instrument. Fluorescence images were captured using a TCS SP8 confocal laser scanning microscope (Lecia). Cell apoptosis detection was conducted via FCM (BECKMAN COULTER), and the cytotoxicity experiment was carried out using a multifunctional microporous reader (SpectraMax i3x). The morphology structures of IMZF were observed by the TEM (FEI Company, USA). Dynamic light scattering (DLS) and zeta potential measurements were conducted by a ZS nanohybrid analyzer (Malvern, England). Ultraviolet-visible-near infrared (UV–vis–NIR) absorption spectra of different samples were recorded by an Evolution 220 UV–vis spectrophotometer (UV-2700i, Japan). The surface element states were analyzed using XPS (ESCA Lab 250, Thermo Fisher Scientific, USA) experiments. ICP-OES were conducted by Agilent 5110 ICP-OES.

**Synthesis section**

Preparation of **compound 1**

In a three-neck flask, 5-Iodo-2,3,3-trimethyl-3H-indole (1.5 g, 6.31 mmol) and 4-Iodobenzyl bromide (3.93 g, 25.20 mmol) were combined with 20 mL of acetonitrile and placed in a water bath for 12 h. Following the heating process, the acetonitrile solvent was removed using a rotary evaporator. A small quantity of DCM was then introduced, and upon complete dissolution, a significant amount of n-hexane was added. After allowing the mixture to stand for a designated period, it was subjected to vacuum filtration, resulting in the isolation of a pale red solid identified as compound 1 (4.1 g, 87% yield).

Preparation of **compound 2**

POCl_3_ (2.1 mL) was slowly added to 9 mL of DMF in an ice bath and allowed to react for 2 h. The solution was then stirred at room temperature for an additional 3 h. Next, 2,4,5-Trifluorophenylaceticacid (1.48 g) was introduced, and the mixture was heated to 90 °C, condensed, and refluxed for 24 h. Upon cooling to room temperature, crushed ice was added, followed by NaClO_4_. The resulting solid was filtered, and washed twice with a saturated solution of sodium perchlorate, yielding the intermediate product. This intermediate was utilized without further purification and added to a 20 mL NaOH solution (0.8 g, 10 mmol), heated, and stirred at 90 °C until complete dissolution. After cooling to room temperature, 10 mL of deionized water was added for dilution. The pH of the solution was adjusted to 2 with 20% HCl to precipitate **compound 2** (pale yellow solid, 1.23 g, 72% yield).

Preparation of **ICy5**

In a three-necked flask, 0.5 mmol of condensation agent (1 equiv) and 1 mmol of quaternary ammonium salt (2 equiv) were introduced. Following this, 10 mL of absolute ethanol and three drops of pyridine were added. The reaction temperature was elevated to 75 °C, and the reaction proceeded under condensation and reflux conditions for 10 h. Monitoring was carried out via Thin-Layer Chromatography (TLC) analysis. After the reduction of the yellow intermediate, the solvent was evaporated under reduced pressure. The resulting residue was adsorbed onto silica gel, and separation was accomplished through column chromatography (DCM/MeOH = 20/1) to yield ICy5. **^1^H NMR (400 MHz, DMSO)** δ 8.44 (d, *J* = 14.2 Hz, 2H), 8.15 (s, 2H), 7.75 (d, *J* = 8.3 Hz, 2H), 7.64 (d, *J* = 7.9 Hz, 4H), 7.38 (d, *J* = 8.4 Hz, 2H), 6.78 (d, *J* = 7.9 Hz, 4H), 5.39 (d, *J* = 14.1 Hz, 2H), 5.11 (s, 4H), 4.11 (d, *J* = 5.4 Hz, 1H), 3.22 – 3.11 (m, 3H), 1.73 (s, 10H). **^13^C NMR** **(101 MHz, DMSO)** δ 173.62, 153.79, 144.04, 142.72, 138.04, 137.60, 135.02, 132.03, 129.18, 127.48, 114.11, 102.67, 94.43, 90.53, 55.41, 49.70, 49.08, 46.99, 27.23. **HRMS(ESI):** m/z calc. for [C_45_H_36_F_3_I_4_N_2_]^+^ 1168.90039, found 1168.88246 [M]^+^.

**Scheme S1.** The synthesis route for **ICy5**.

**Fabrication of IMZF**

The fluoride ZIF-8 doped with manganese (MZ) was synthesized following a procedure akin to that of ZIF-8, where Zn^2+^ was substituted with Mn^2+^ and 2-MIM was partially replaced by TFMIM. Specifically, Zn(OAc)_2_·2H_2_O (0.5 mM), manganese acetylacetonate (0.05 mM), 2-MIM (6.4 mM), TFMIM (1.6 mM), and NH_3_·H_2_O (300 μL) were combined in 50 mL of methanol. The mixture was stirred at 25 °C for 24 h, followed by centrifugation (10,000 g, 10 min) to isolate MZ. Subsequently, ICy5 was introduced into MZ; 5 mL of MZ (10 mg/mL) was mixed with 50 μL of ICy5 (10 mg/mL). After stirring in darkness for 24 h (25 °C), the mixture was centrifuged (10,000 g, 10 min) to obtain ICy5@MZ (IMZ). The loading amount of ICy5 in IMZ was determined using UV-visible-NIR absorption spectroscopy. To prepare F@IMZ (IMZF), 60 μL of TA solution (40 mg/mL) was slowly added to 3 mL of IMZ aqueous solution (5 mg/mL). After vigorous stirring for 10 min, 60 μL of FeCl_2_·4H_2_O solution (40 mg/mL) was slowly added, followed by stirring for another 10 min. The resulting mixture was centrifuged at room temperature at a speed of 15,000 rpm to collect the dark green precipitate. The precipitate was washed twice with deionized water, freeze-dried using water as the medium, and yielded a black-green solid powder.

**Support Figures**


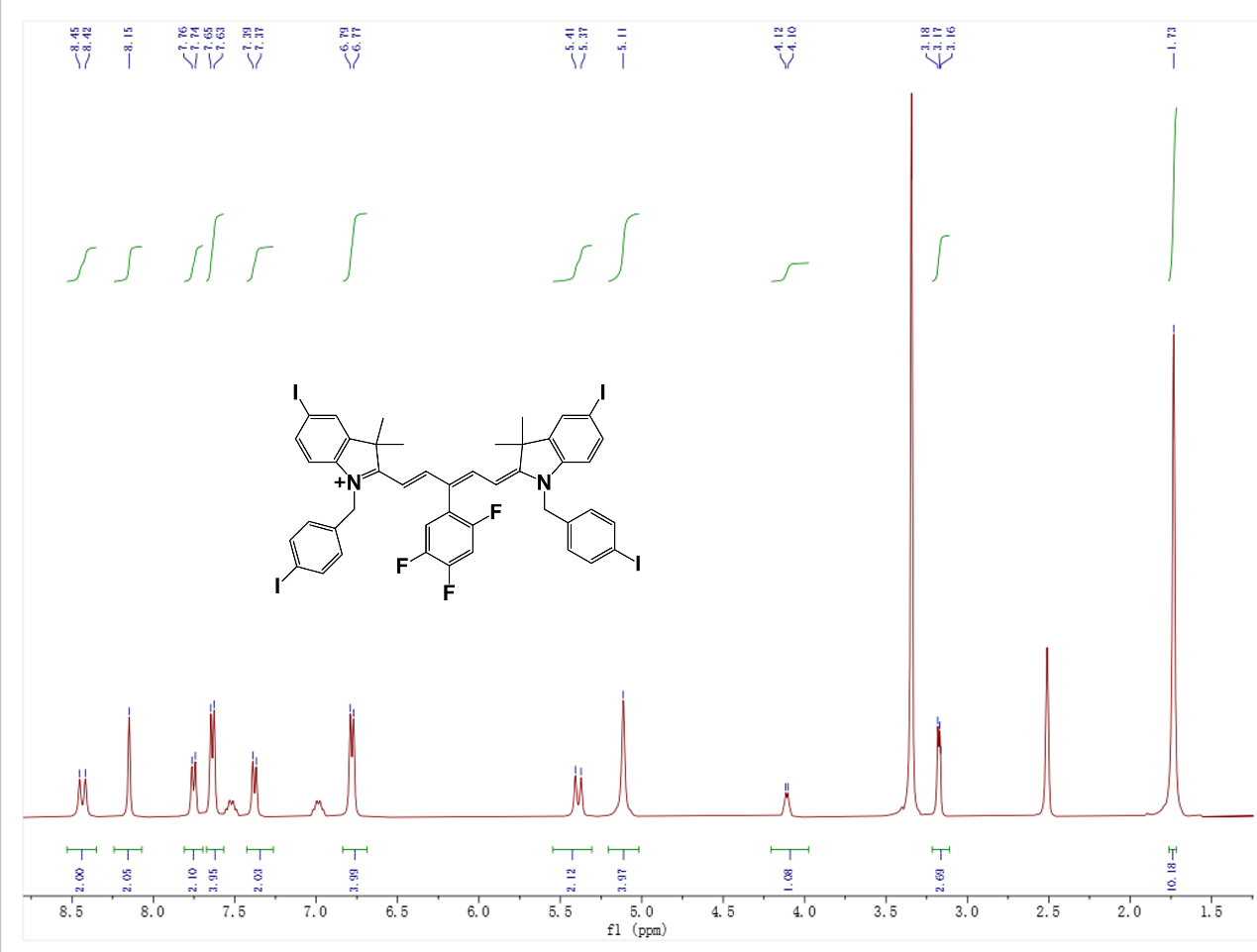


**Figure S1.** ^1^H NMR spectrum of ICy5 (400 MHz, DMSO-d6).


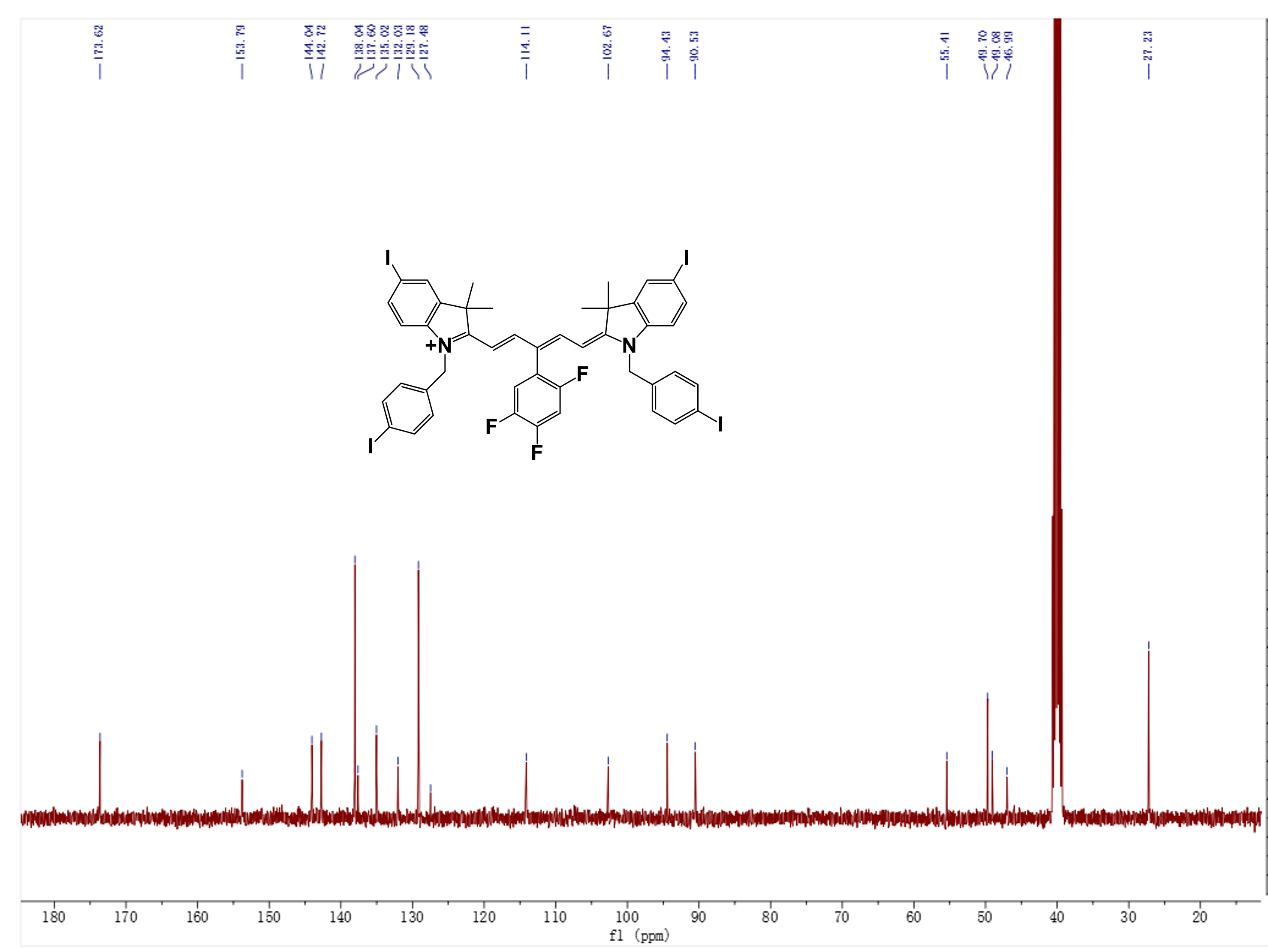
 **Figure S2.** ^13^C NMR spectrum of ICy5 (400 MHz, DMSO-d6).


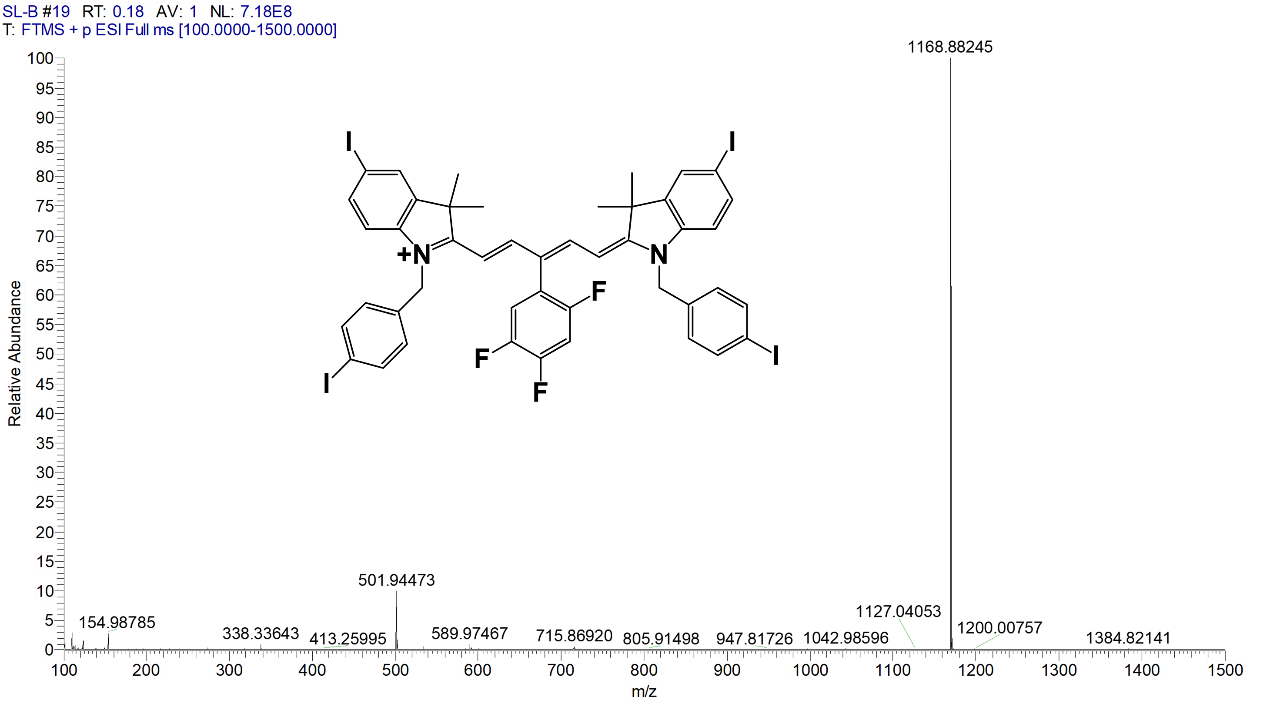


**Figure S3.** Mass spectrum of ICy5.


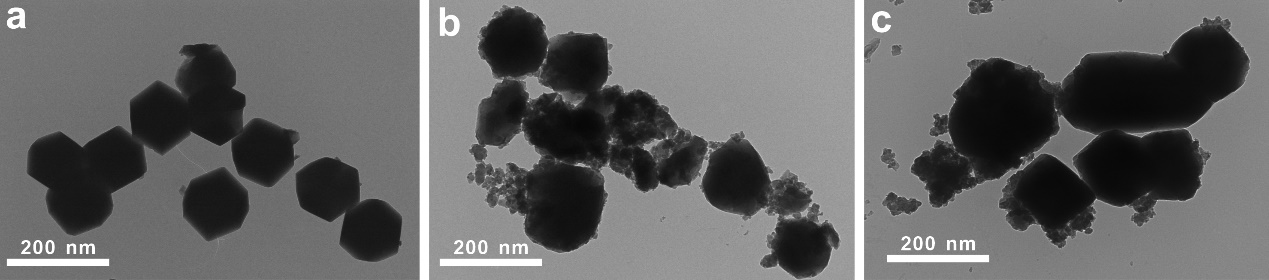


**Figure S4.** (a) TEM of ZIF-8. (b) TEM of IMZ. (c) TEM of IMZF.


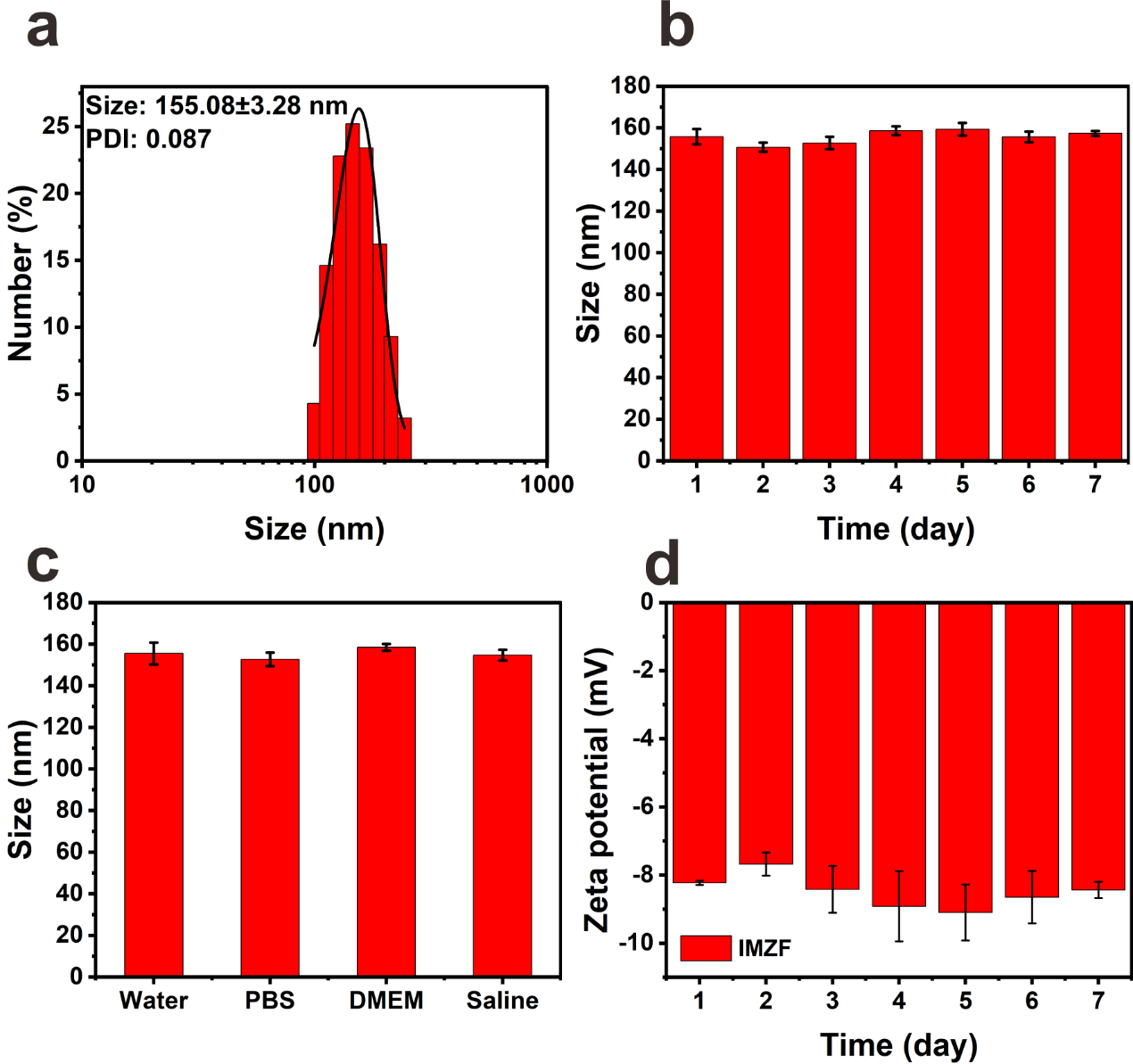


**Figure S5.** (a) The nano size of IMZF in ultrapure water solution. (b) The nano size of IMZF in ultrapure water solution over the past seven days. (c) The nano size variation of IMZF in different solutions. (d) The zeta potential of IMZF under physiological conditions over a period of seven days.


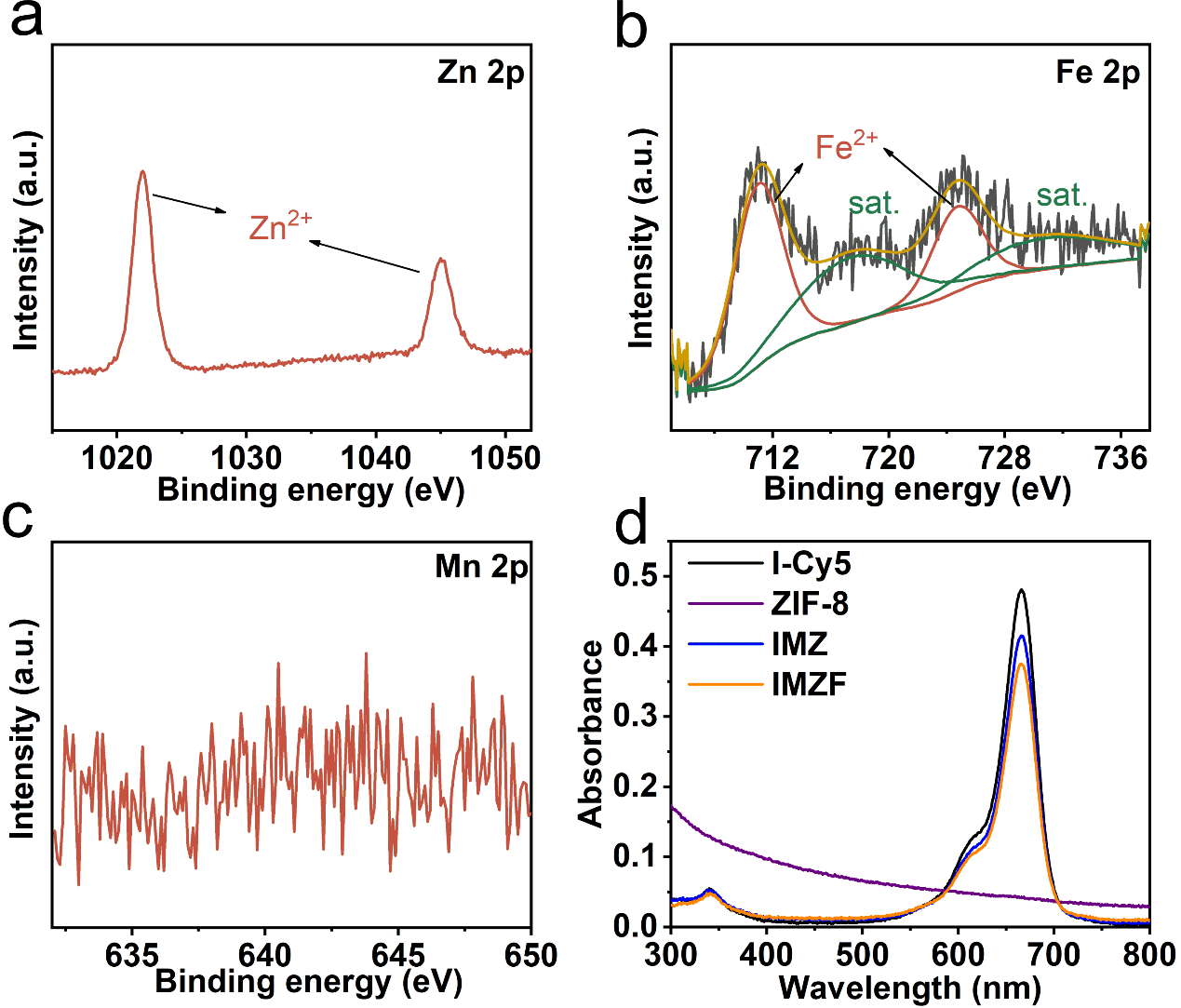


**Figure S6.** (a) Zn 2p spectrum, (b) Fe 2p spectrum and (c) Mn 2p spectrum of IZMF. (d) UV absorption spectra of ICy5, ZIF-8, IMZ and IMZF.


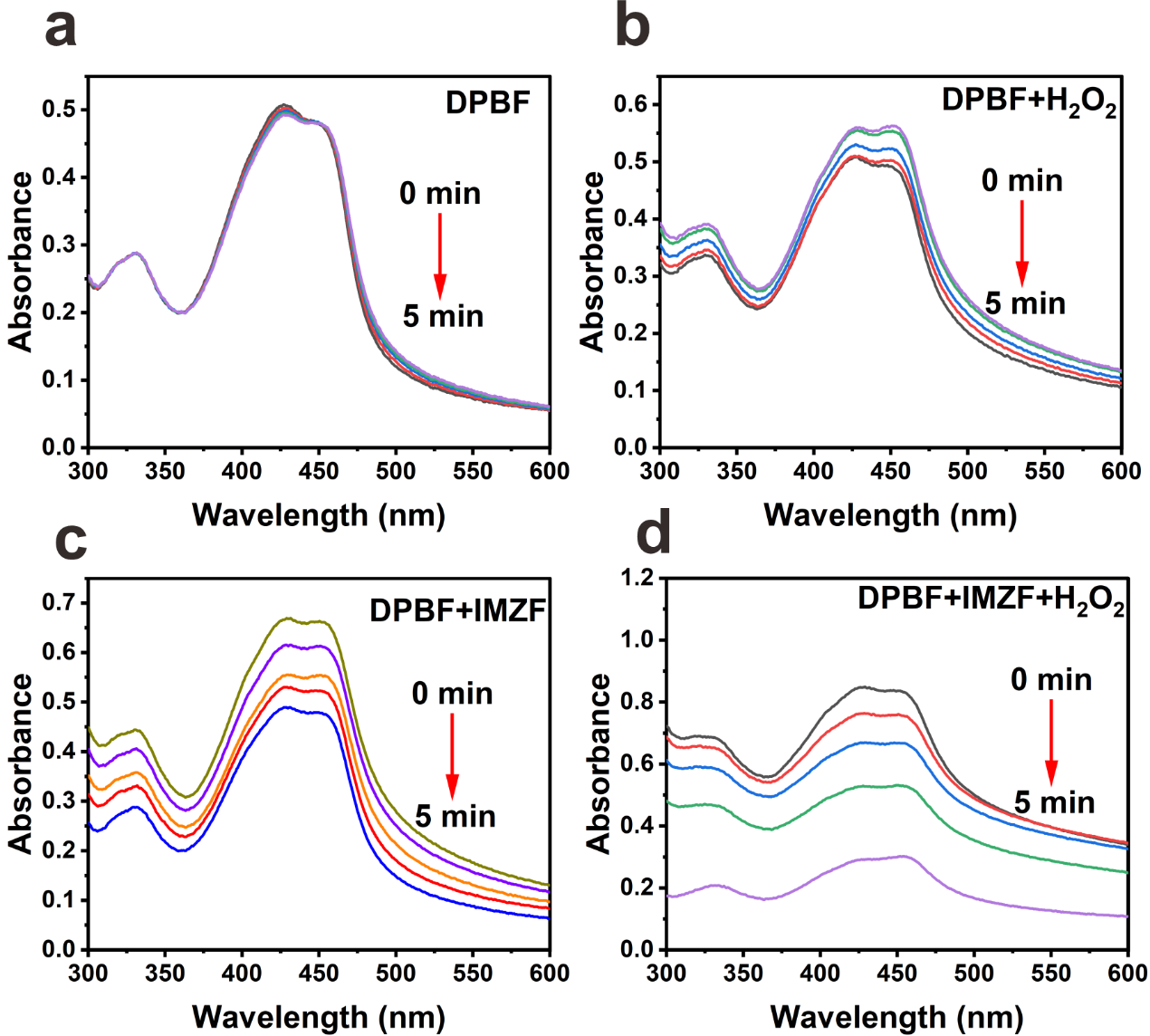


**Figure S7.** (a) ^1^O_2_ generation by DPBF under NIR light irradiation (660 nm, 10 mW/cm^2^) alone. (b) ^1^O_2_ generation under NIR light irradiation (660 nm, 10 mW/cm^2^) after co-incubation of DPBF with H_2_O_2_. (c) ^1^O_2_ generation under NIR light irradiation (660 nm, 10 mW/cm^2^) after co-incubation of DPBF with IMZF. (d) ^1^O_2_ generation under NIR light irradiation (660 nm, 10 mW/cm^2^) after co-incubation of DPBF with IMZF and H_2_O_2_.


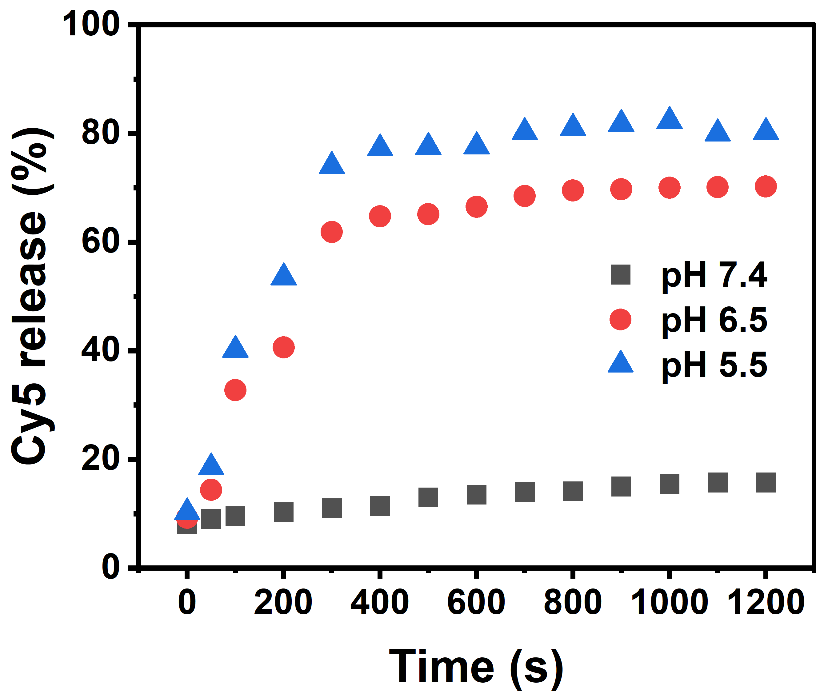


**Figure S8.** Release of ICy5 from IMZF under different conditions.


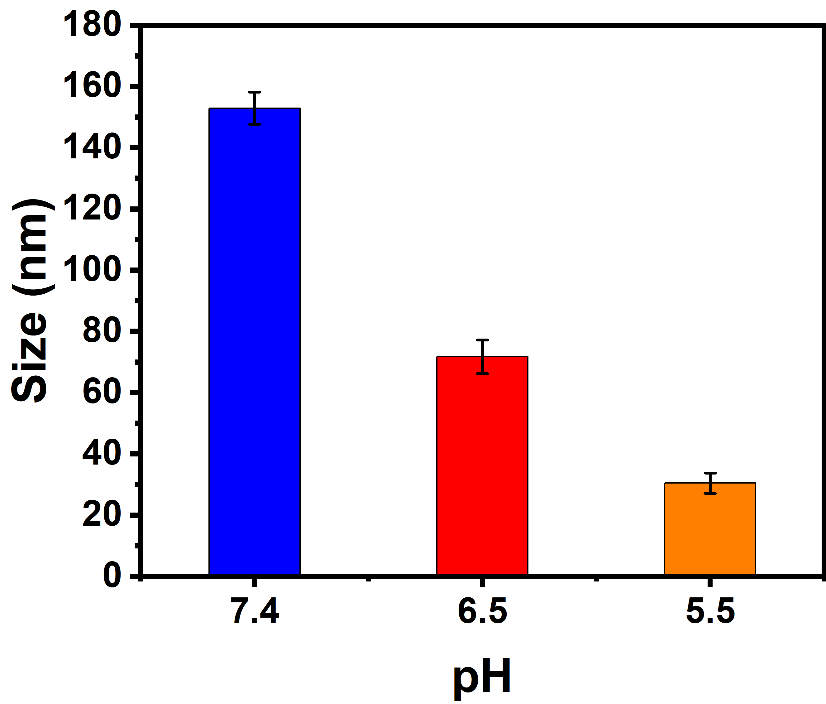


**Figure S9.** Nanoparticle size of IMZF at different pH values.


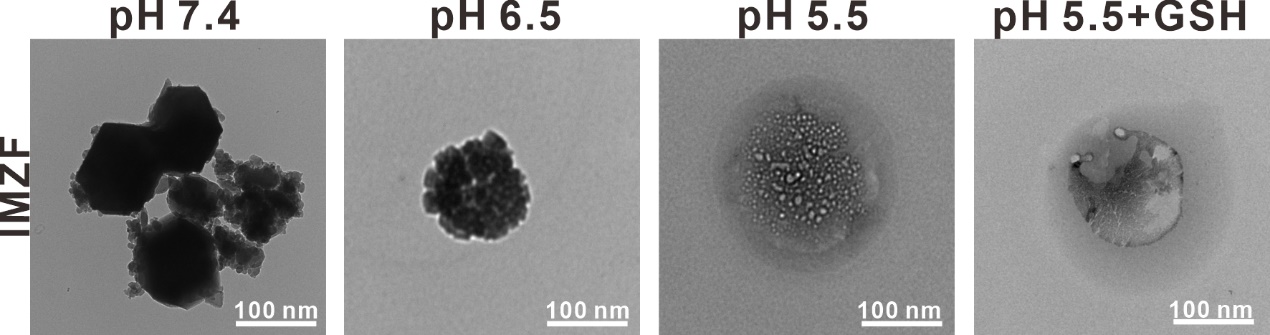


**Figure S10.** TEM images of IMZF after immersion in PB buffer (pH 7.4, pH 6.5, pH 5.5, and pH 5.5 plus GSH (10 mM) for 2 h.


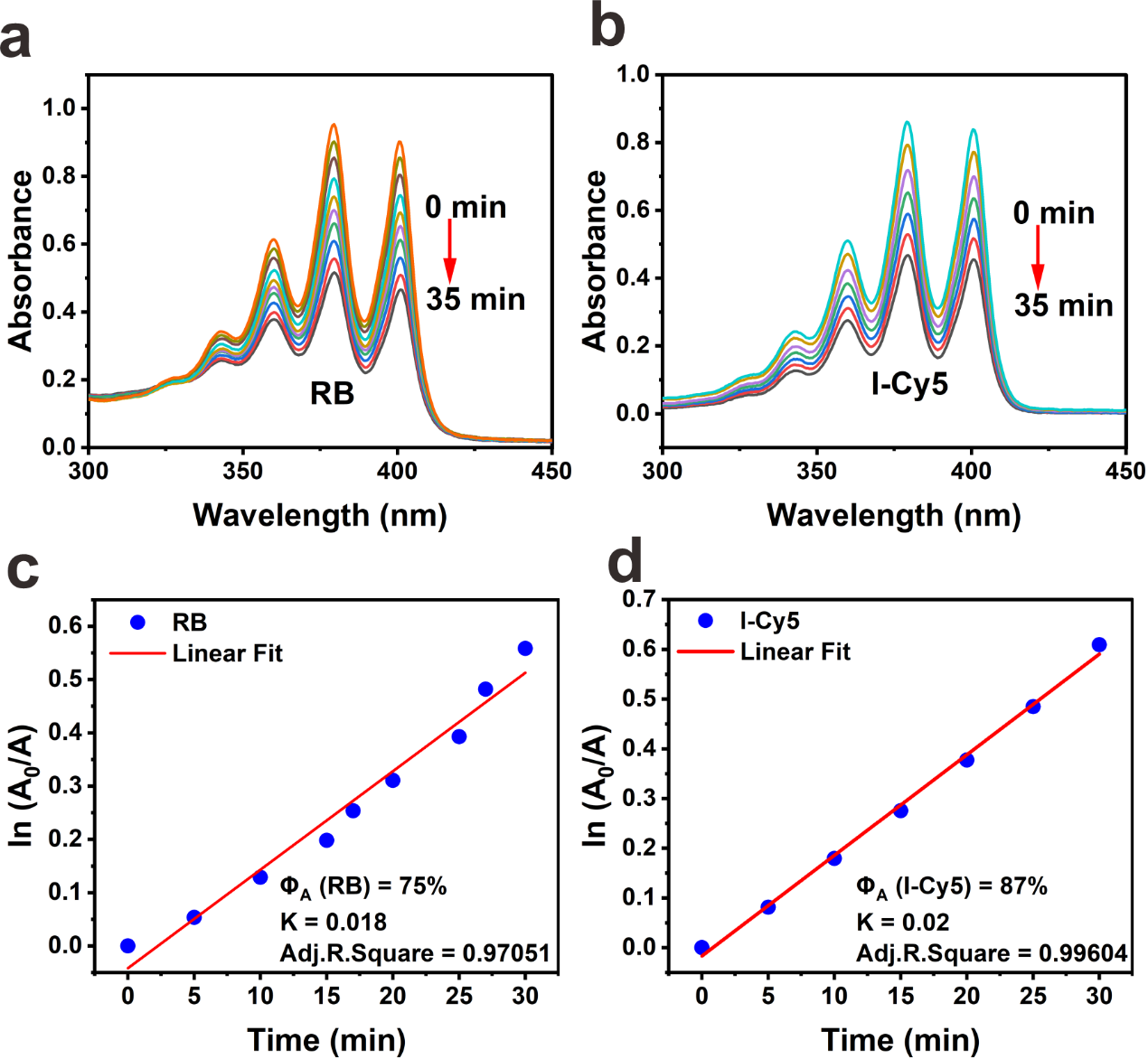


**Figure S11.** Ultraviolet-visible absorption spectrum of ABDA in (A) Rose Bengal (RB) (1×10^-5^ M) (B) I-Cy5 (1×10^-5^ M) irradiated for different durations with light irradiation (550 nm and 660 nm, 5 mW/cm^2^). (c) RB and (d) ICy5 were the fitting lines based on ABDA in a 380 nm aqueous solution under different light times.


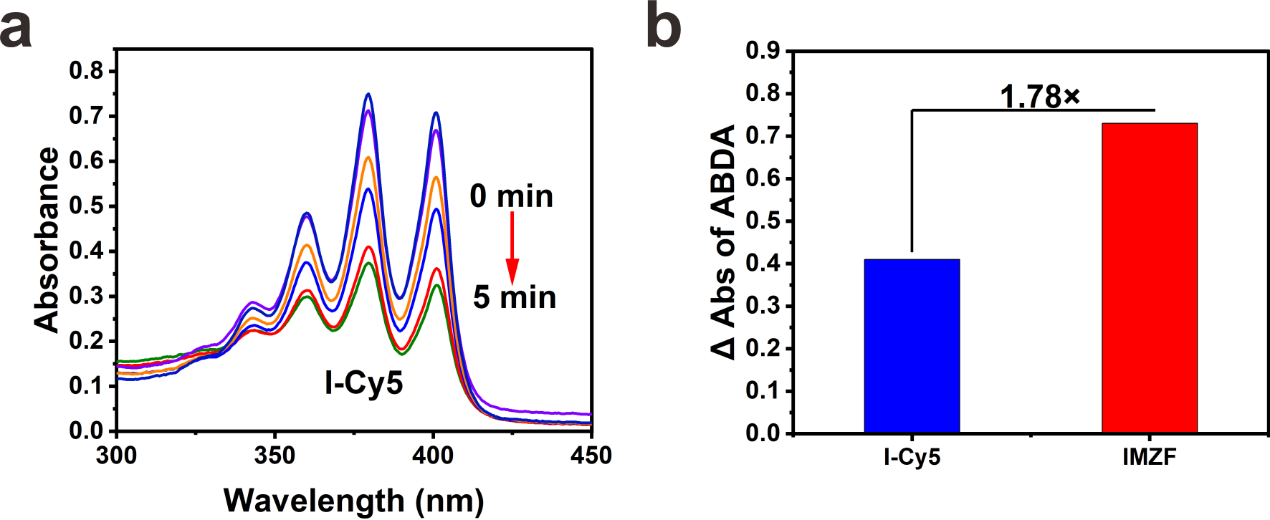


**Figure S12.** (a) Using ABDA to detect the generation of ^1^O_2_ by I-Cy5 under NIR light irradiation (660 nm, 10 mW/cm^2^) for 5 min. (b) Comparison of ^1^O_2_ generation by ICy5 and IMZF within 5 min.


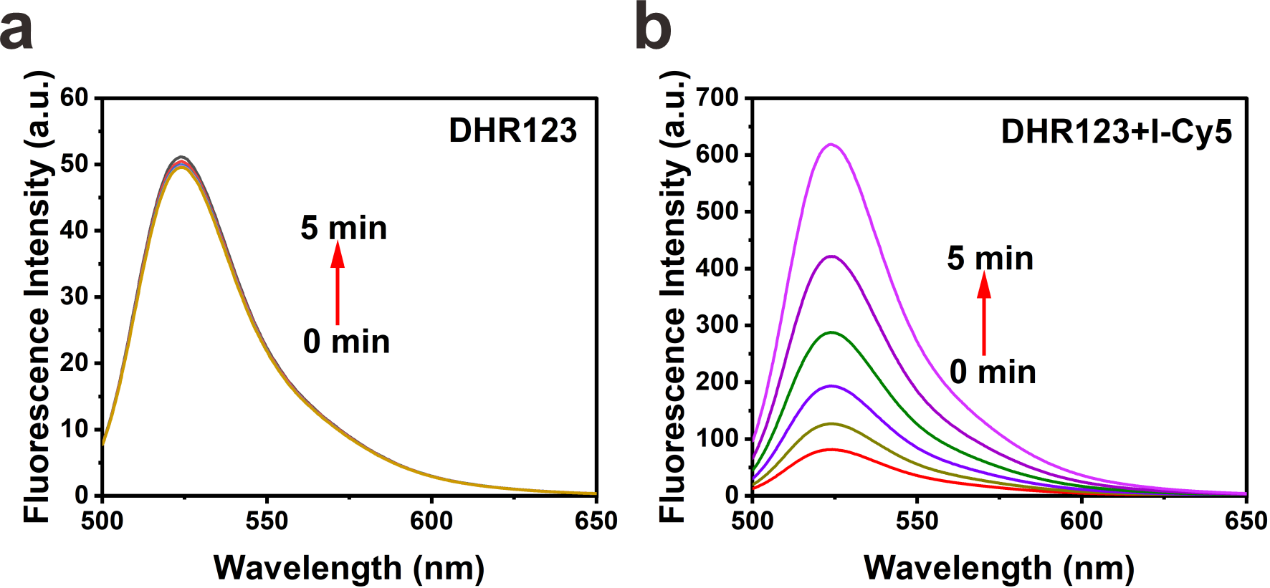


**Figure S13.** (a) ROS generation by DHR123 under NIR light irradiation (660 nm, 10 mW/cm^2^) alone. (b) ROS generation under NIR light irradiation (660 nm, 10 mW/cm^2^) after co-incubation of DHR123 with ICy5.


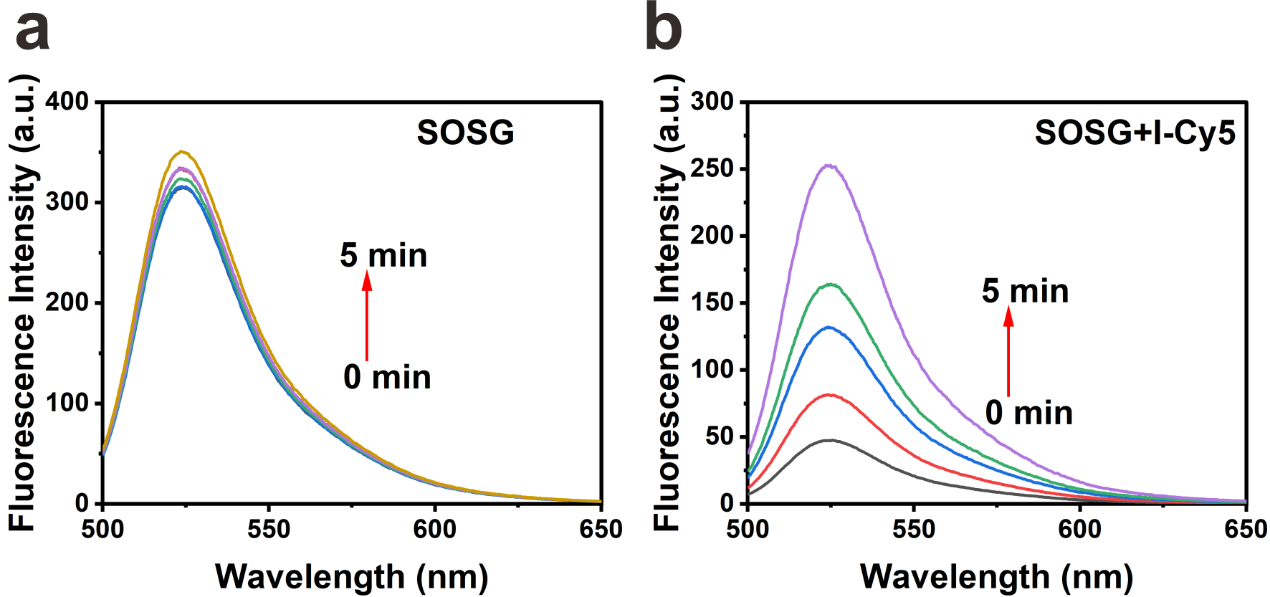


**Figure S14.** (a) ^1^O_2_ generation by SOSG under NIR light irradiation (660 nm, 10 mW/cm^2^) alone. (b) ^1^O_2_ generation under NIR light irradiation (660 nm, 10 mW/cm^2^) after co-incubation of SOSG with ICy5.


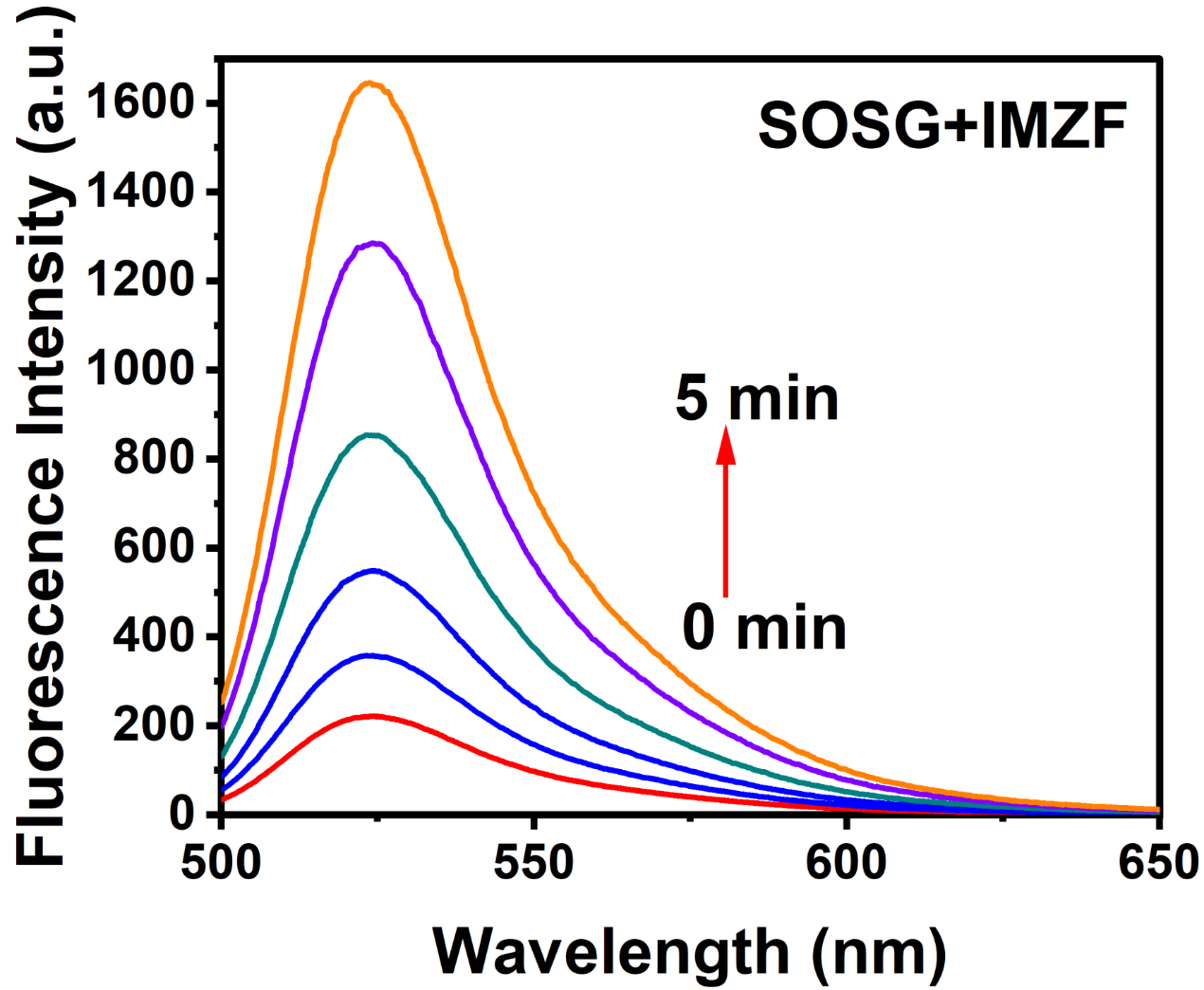


**Figure S15.** ^1^O_2_ generation under NIR light irradiation (660 nm, 10 mW/cm^2^) after co-incubation of SOSG with IMZF.


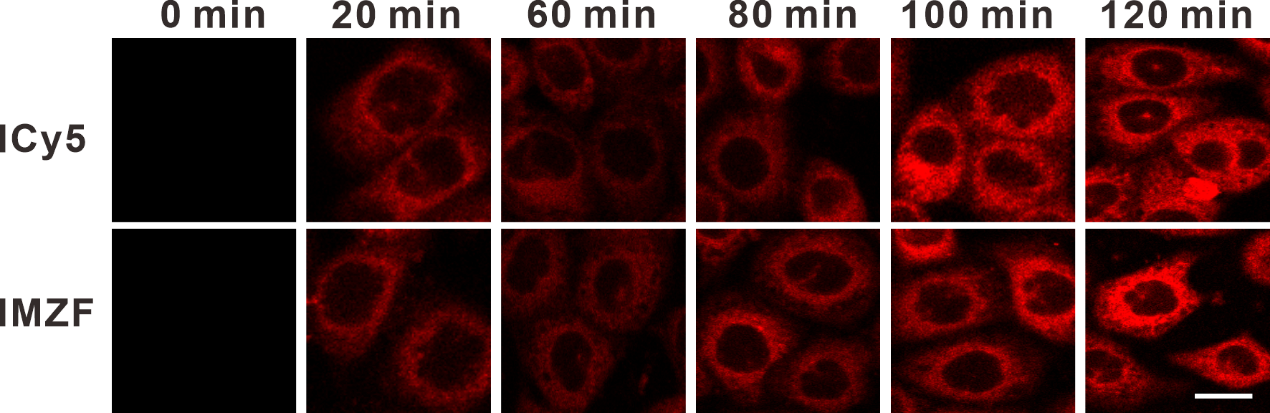


**Figure S16.** Cellular uptake of IMZF in MCF-7 cells. Emissions are collected at 750-800 nm (λ_ex_ = 640 nm).


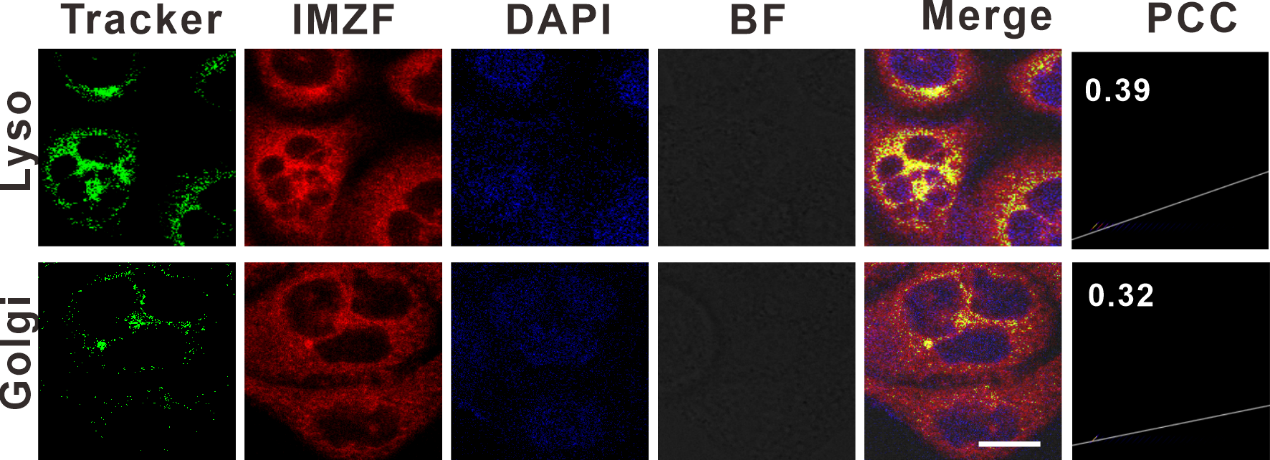


**Figure S17.** Spontaneous Lyso-localizability and Golgi-localizability of IMZF. Representative merged CLSM images of MCF-7 cells treated with 0.5 μM of Lyso/Golgi-Tracker Green and 50 μg/mL of IMZF (red) for 2 h. For the Lyso/Golgi-Tracker Green, emissions were collected at 500-600 nm (λ_ex_ = 490 nm), For the IMZF probes, emissions were collected at 750-800 nm (λ_ex_ = 640 nm).


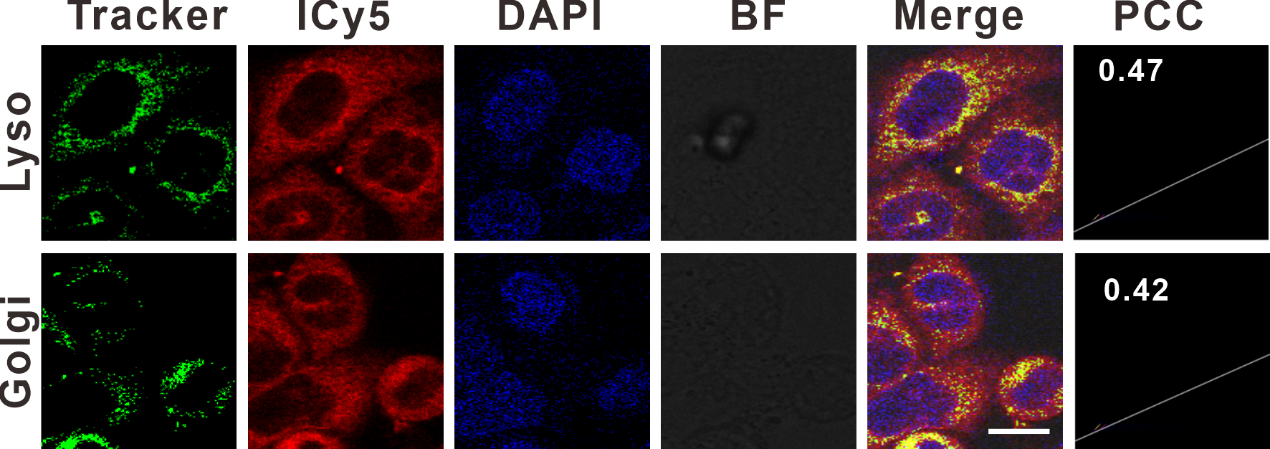


**Figure S18.** Spontaneous Lyso-localizability and Golgi-localizability of ICy5. Representative merged CLSM images of MCF-7 cells treated with 0.5 μM of Lyso/Golgi-Tracker Green and 1 μM of ICy5 (red) for 2 h. For the Lyso/Golgi-Tracker Green, emissions were collected at 500-600 nm (λ_ex_ = 490 nm), For the ICy5 probes, emissions were collected at 750-800 nm (λ_ex_ = 640 nm).

**
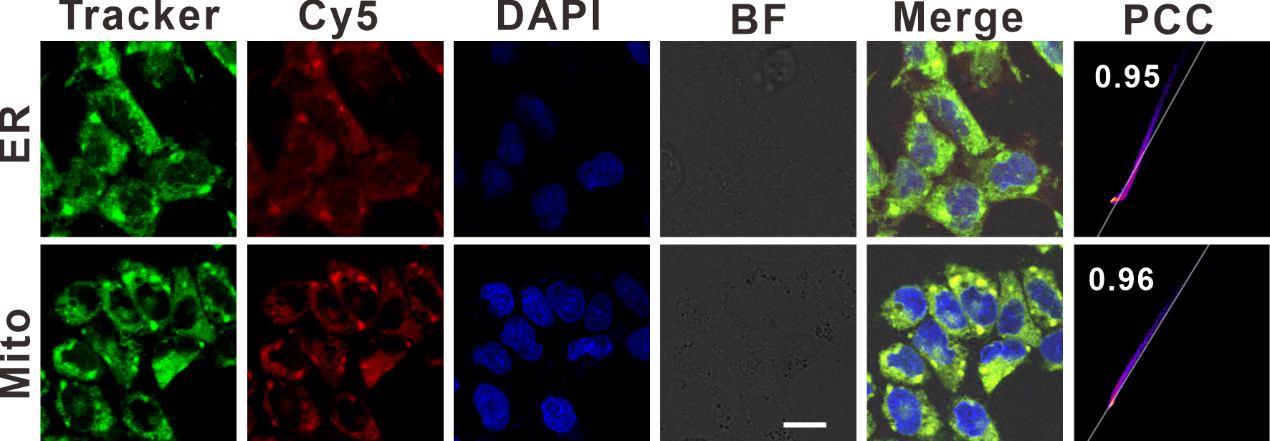
**

**Figure S19.** Representative merged CLSM images of 4T1 cells treated with 0.5 μM of ER-Tracker Green and 50 μg/mL IMZF of (red) for 2 h. For the ER-Tracker Green or Mito-Tracker Green, emissions were collected at 500-600 nm (λ_ex_ = 504 nm), For the IMZF, emissions were collected at 650-730 nm (λ_ex_ = 640 nm).


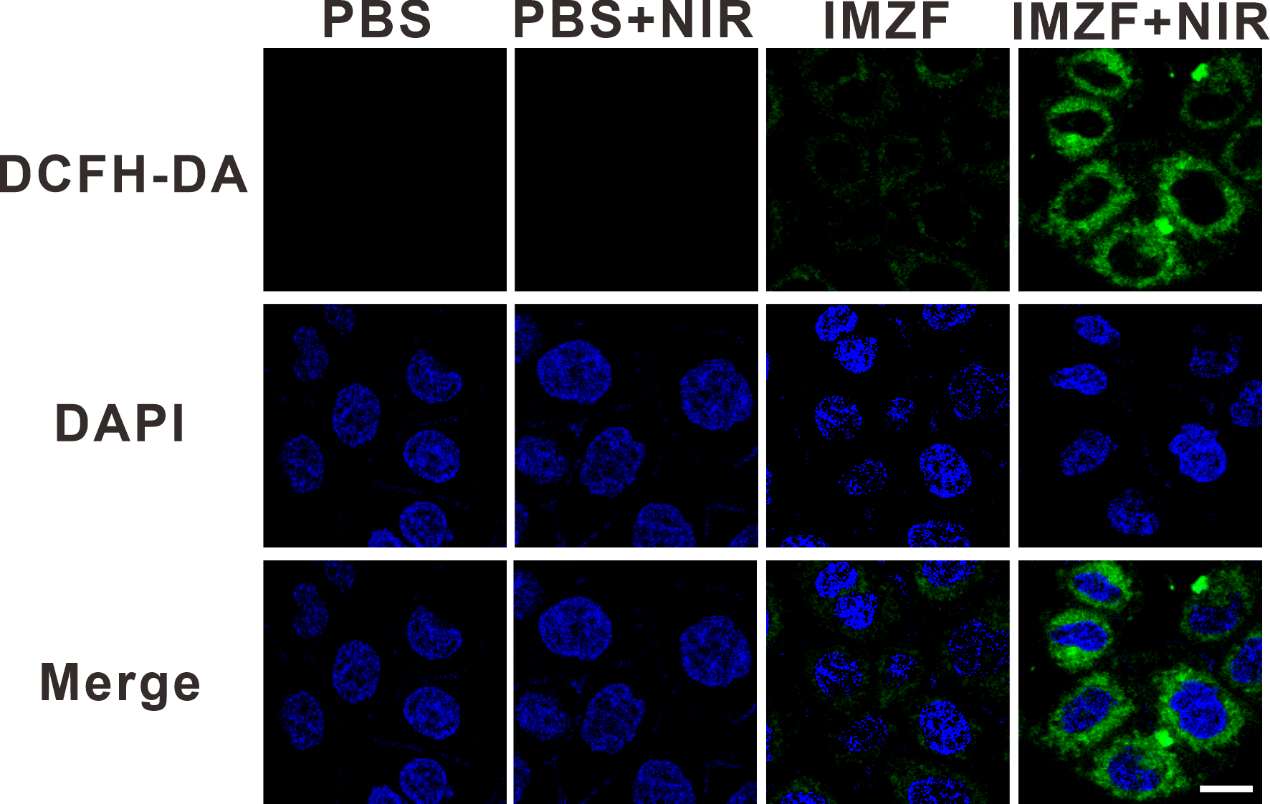


**Figure S20.** Detection of ROS generation under hypoxic conditions using the DCFH-DA probe. For the DCFH-DA, emissions were collected at 500-600 nm (λ_ex_ = 488 nm).


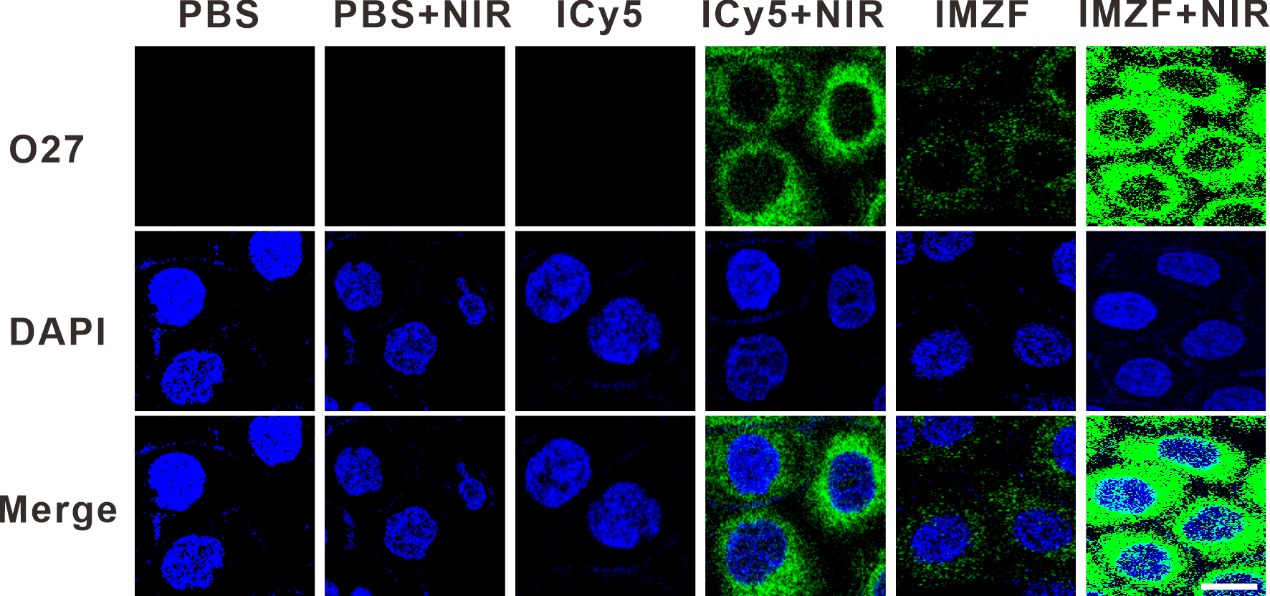


**Figure S21.** The O27 probe is utilized for detecting the intracellular generation of •OH. For the O27, emissions were collected at 500-600 nm (λ_ex_ = 488 nm).

**
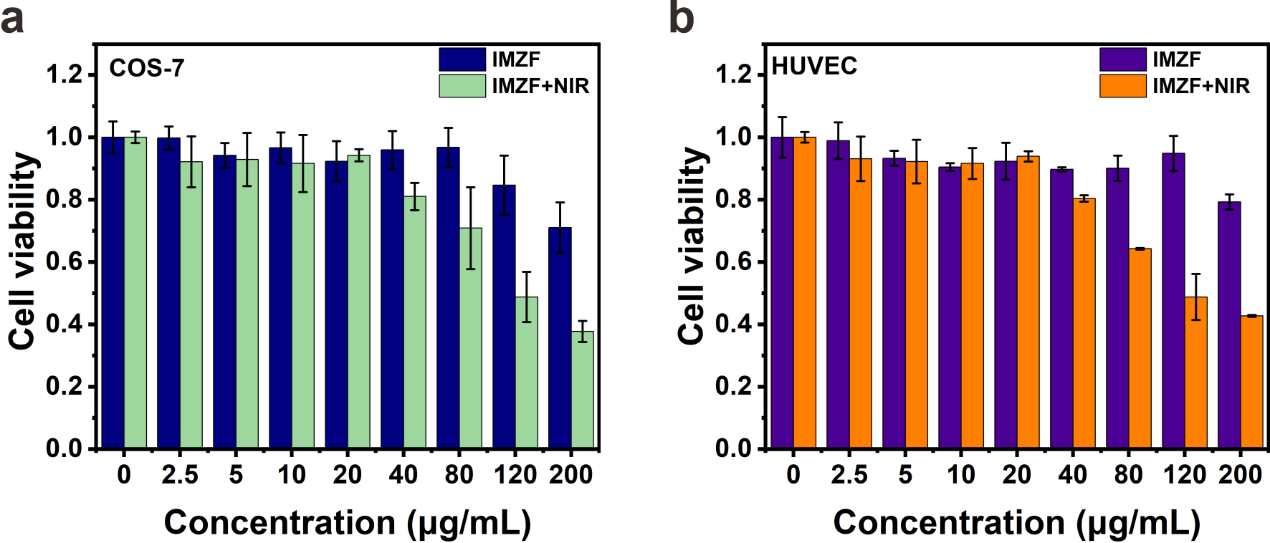
**

**Figure S22.** Phototoxicity of three compounds to COS-7 cells and HUVEC cells under 660 nm (10 mW/cm^2^, 5 min) light irradiation.


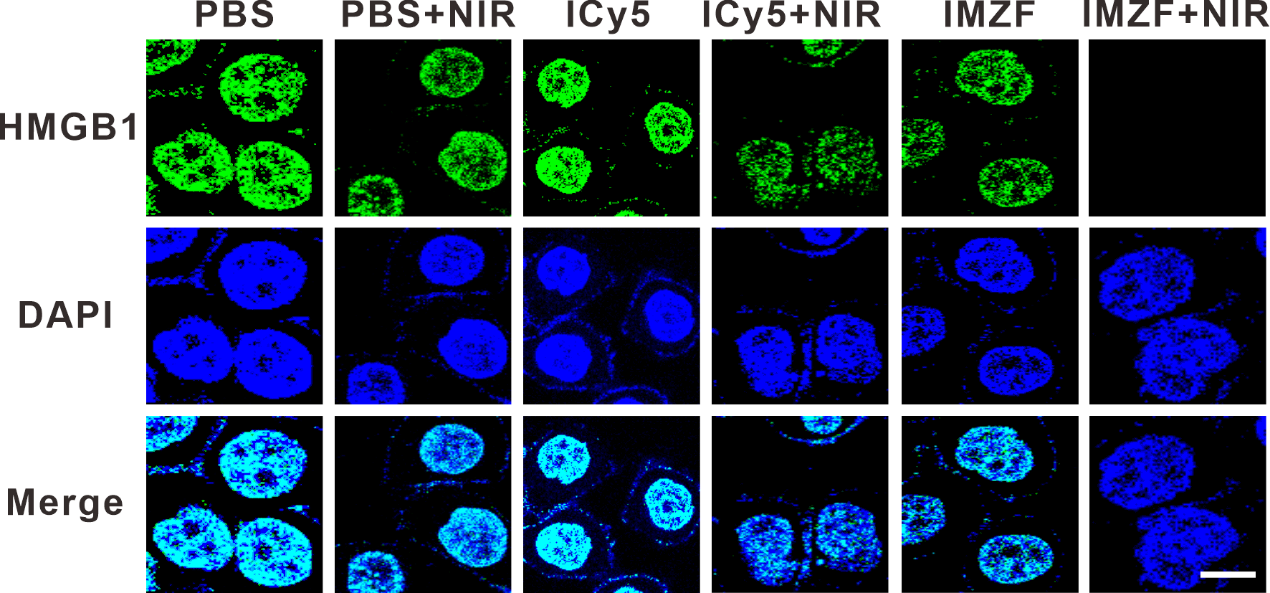


**Figure S23.** The protein fluorescence of HMGB1 was measured using fluorescence detection. For the HMGB1, emissions were collected at 500-600 nm (λ_ex_ = 488 nm).


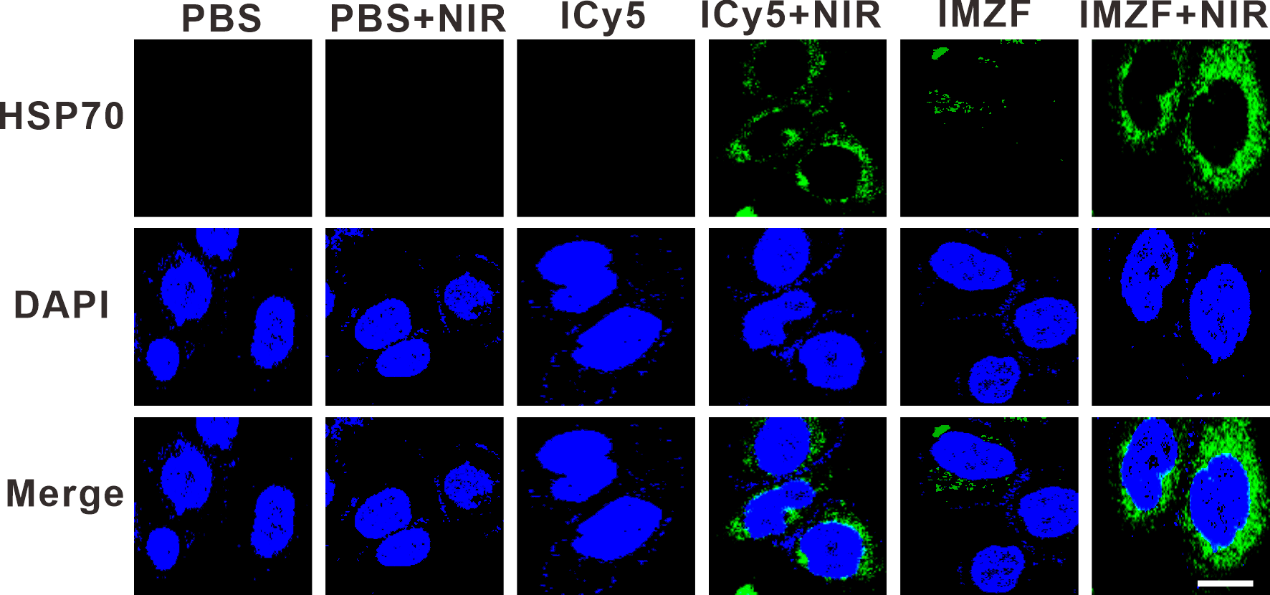


**Figure S24.** The protein fluorescence of HSP70 was measured using fluorescence detection. For the HSP70, emissions were collected at 500-600 nm (λ_ex_ = 488 nm).


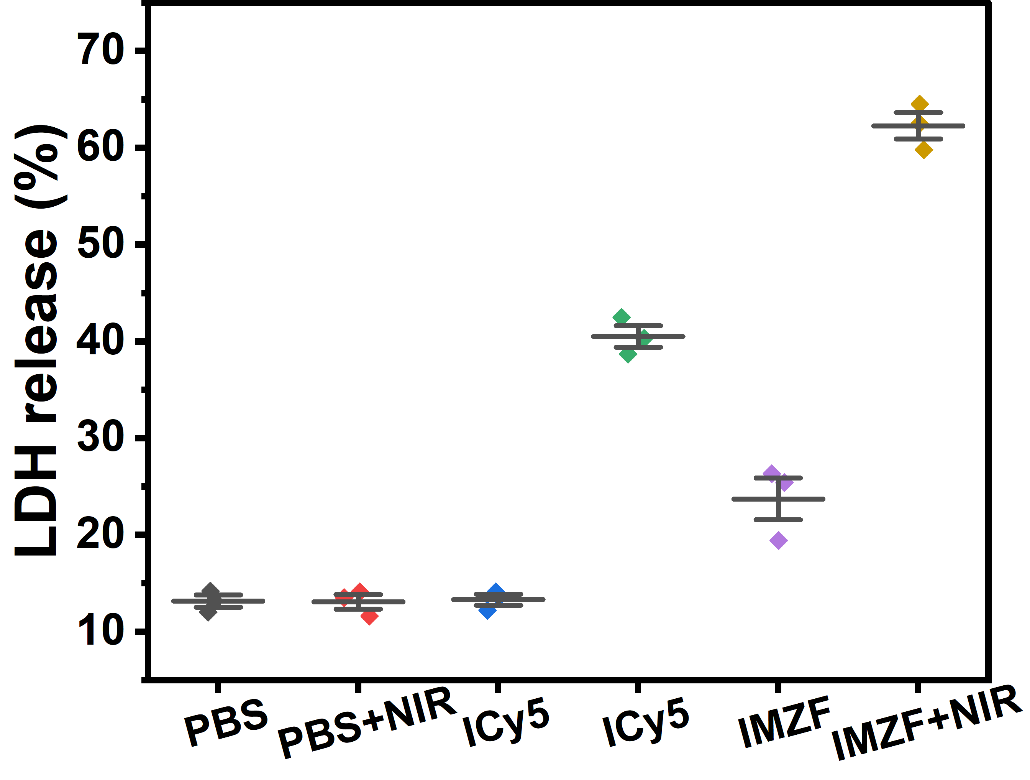


**Figure S25.** LDH detection in the supernatants of different treatment groups.


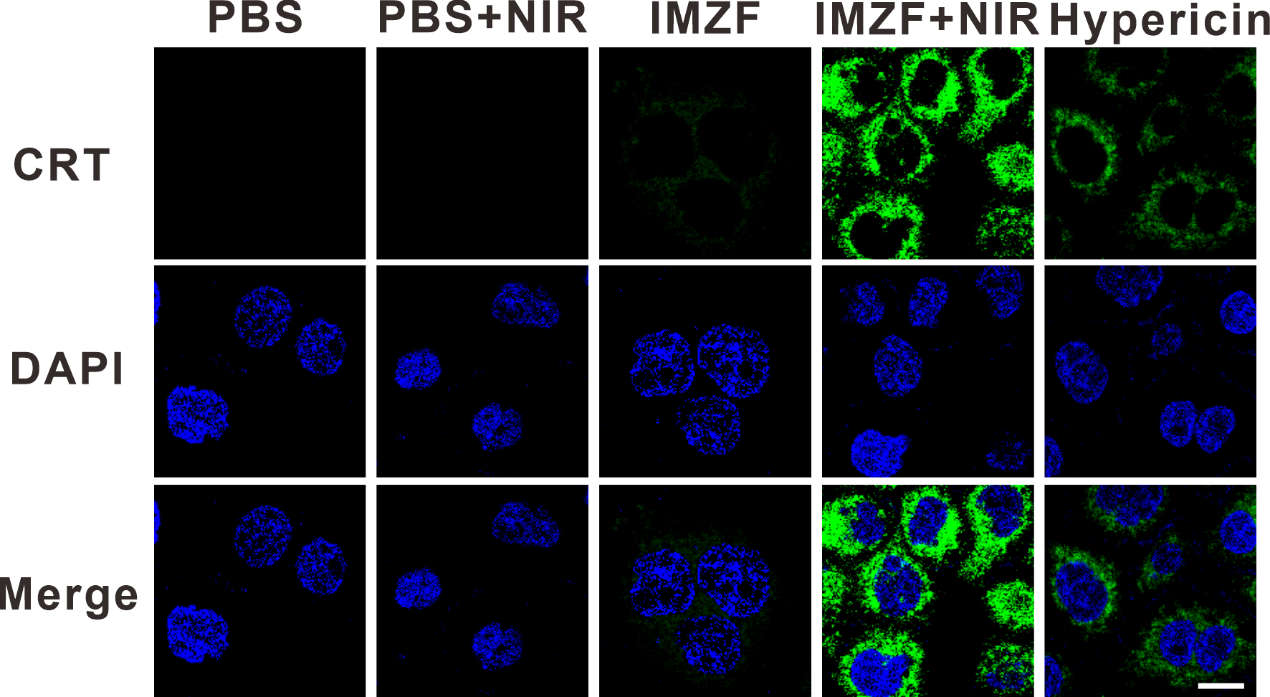


**Figure S26.** The protein fluorescence of CRT was measured using fluorescence detection. For the HSP70, emissions were collected at 500-600 nm (λ_ex_ = 488 nm).


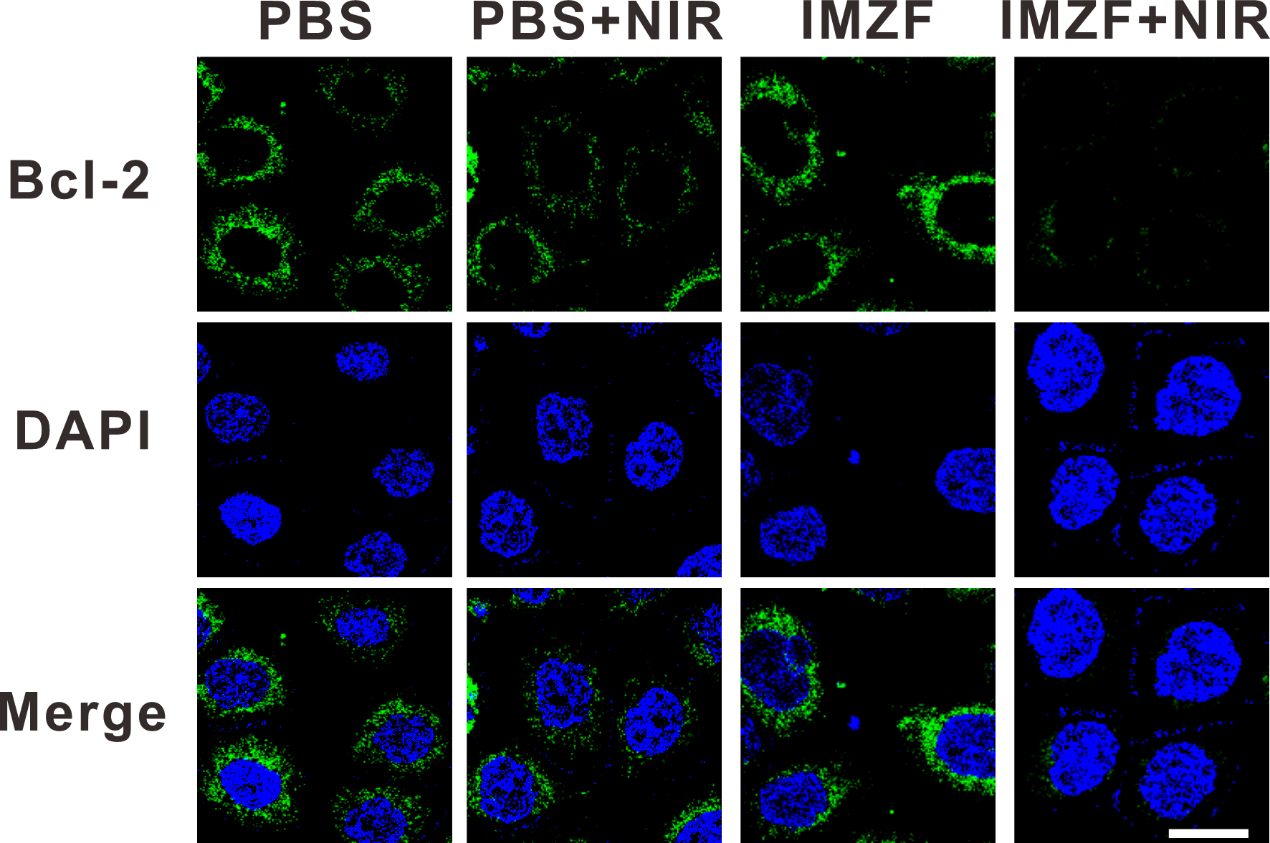


**Figure S27.** The protein fluorescence of Bcl-2 was measured using fluorescence detection. For the Bcl-2, emissions were collected at 500-600 nm (λ_ex_ = 488 nm).


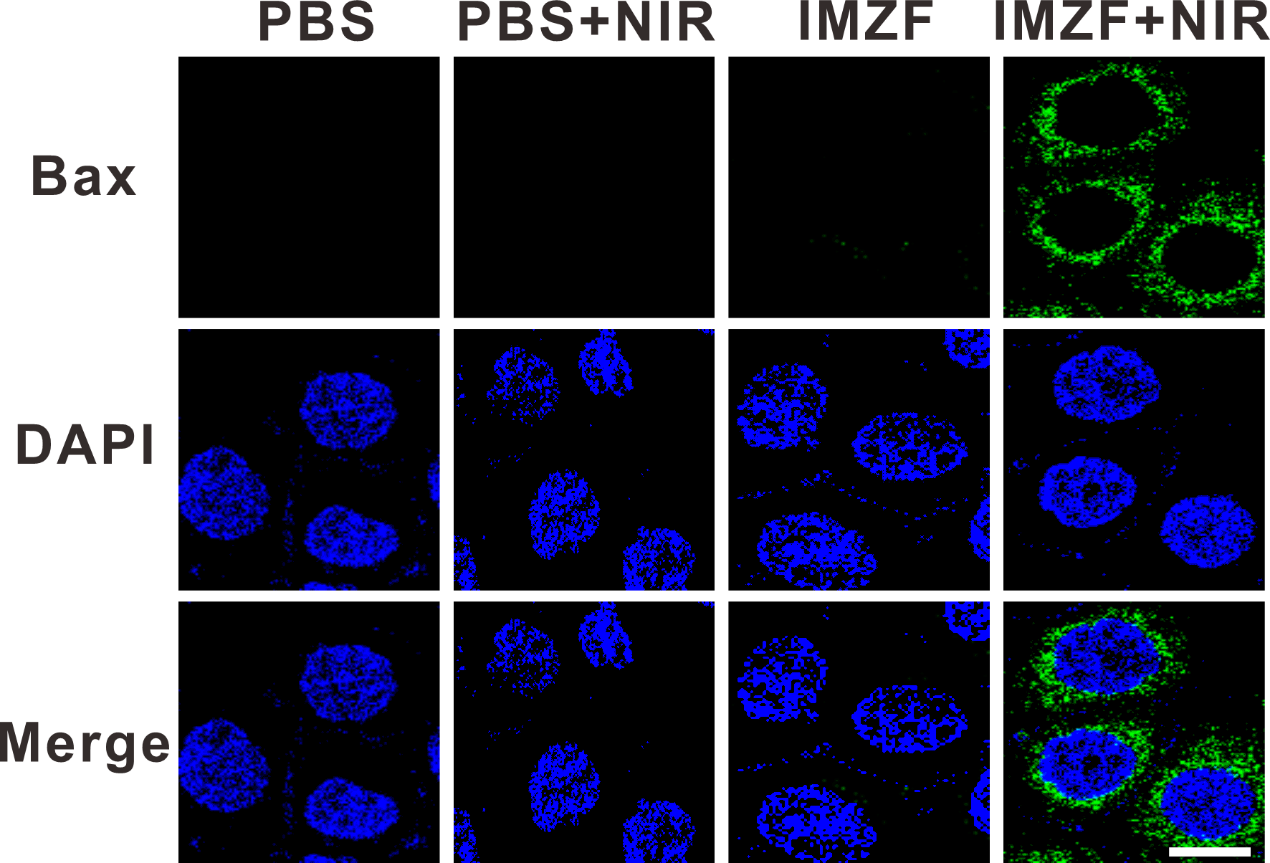


**Figure S28.** The protein fluorescence of Bax was measured using fluorescence detection. For the Bax, emissions were collected at 500-600 nm (λ_ex_ = 488 nm).


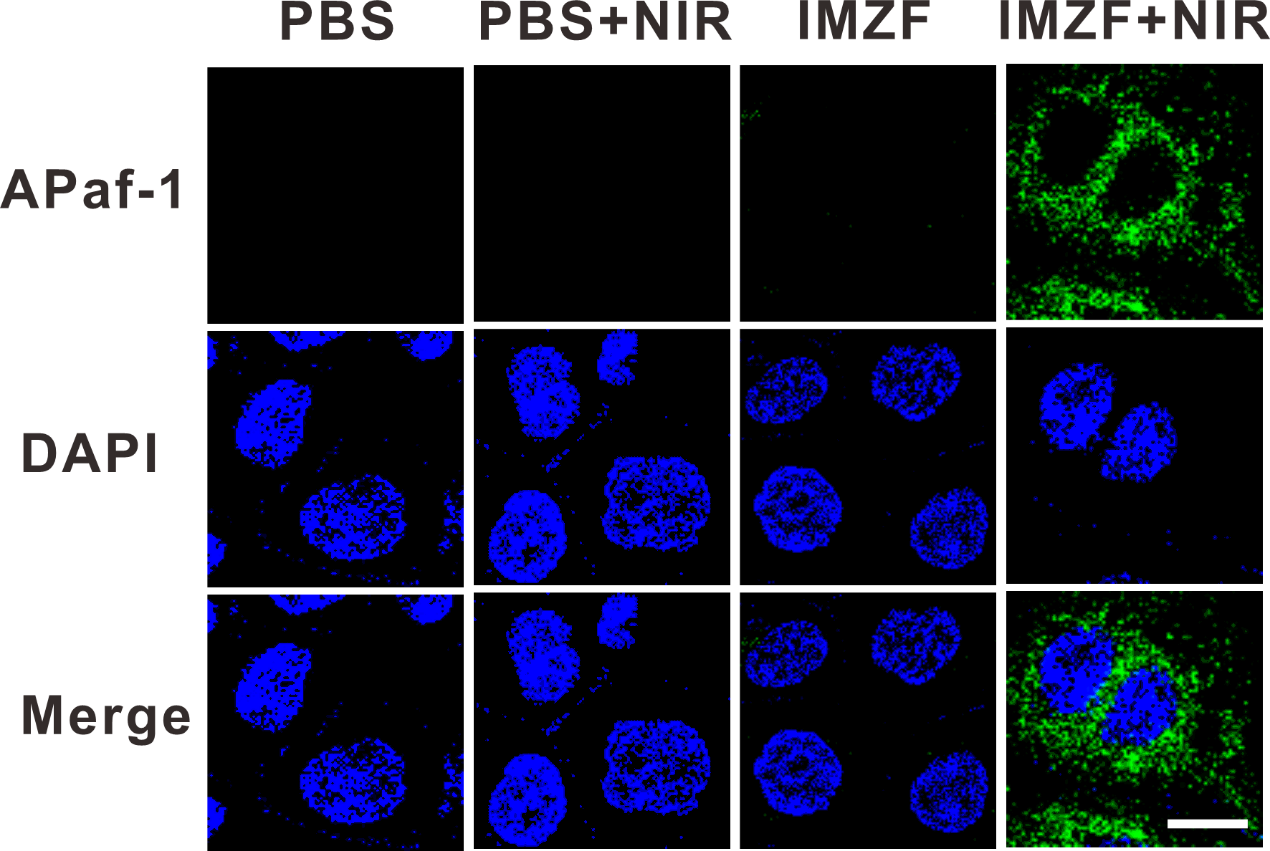


**Figure S29.** The protein fluorescence of Apaf-1 was measured using fluorescence detection. For the Apaf-1, emissions were collected at 500-600 nm (λ_ex_ = 488 nm).


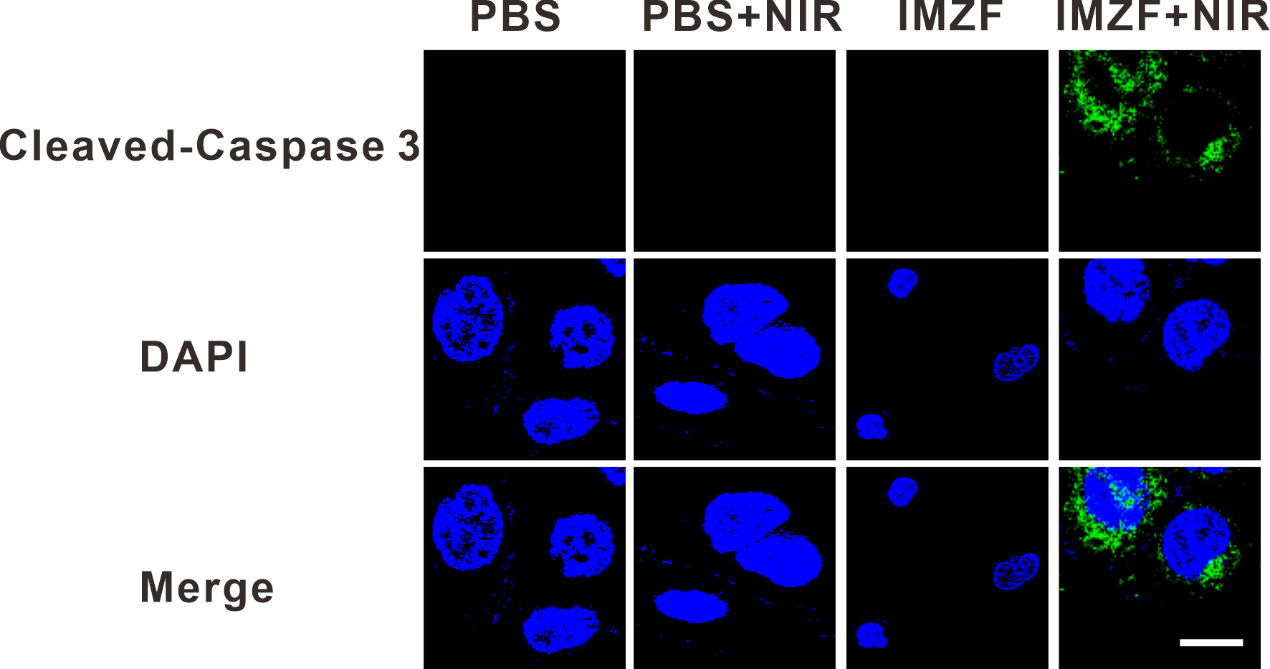


**Figure S30.** The protein fluorescence of Cleaved-caspase 3 was measured using fluorescence detection. For the Cleaved-caspase 3, emissions were collected at 500-600 nm (λ_ex_ = 488 nm).


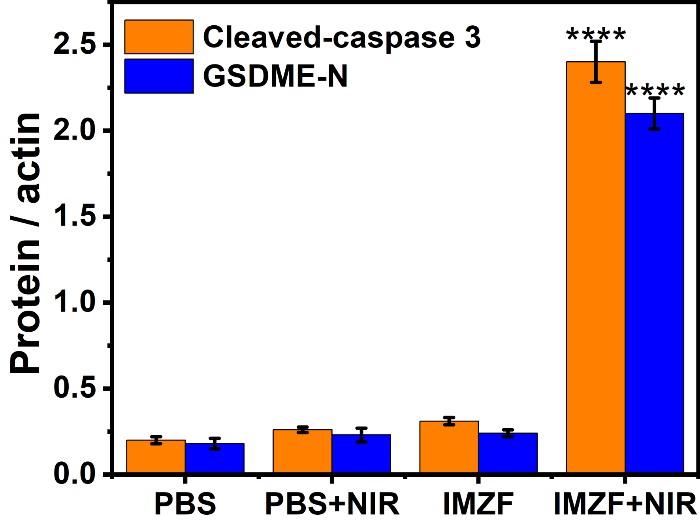


**Figure S31.** Quantitative analysis via western blotting. (*p<0.05, **p<0.01, ***p<0.001, ****p<0.0001).


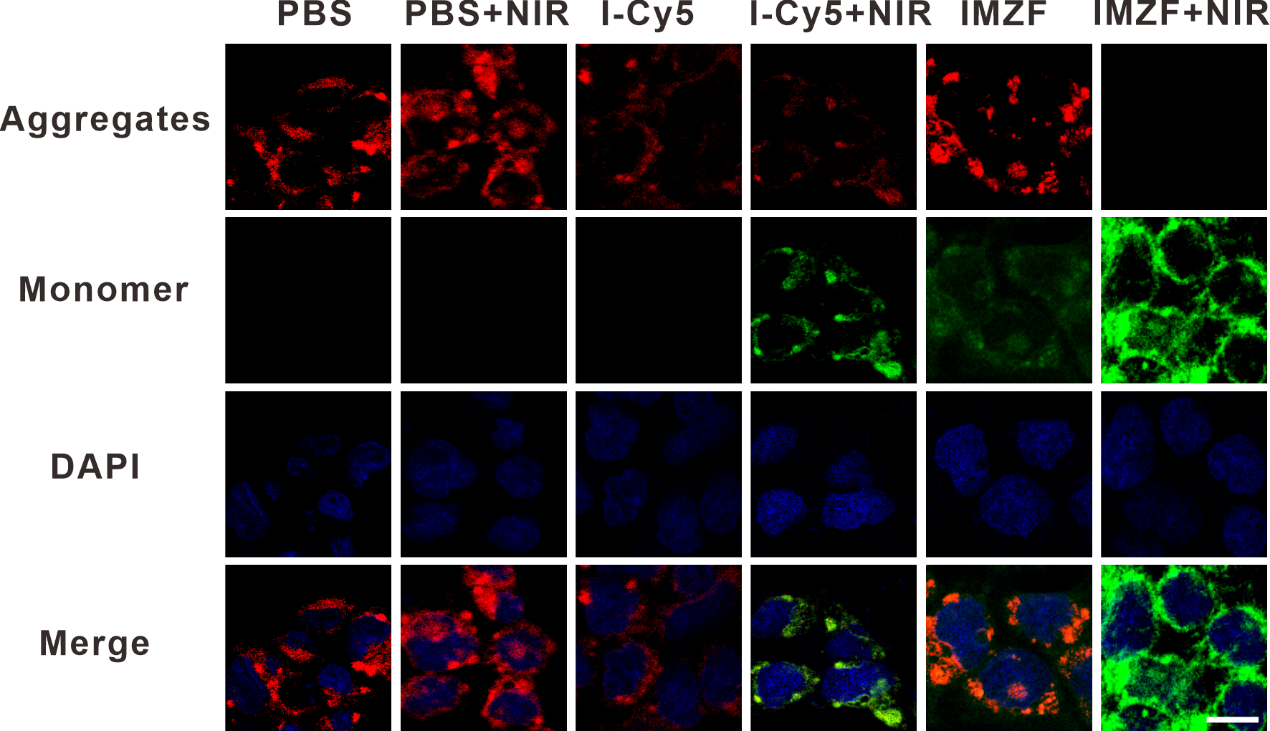


**Figure S32.** Detection of Membrane Potential in Different Treatment Groups of 4T1 Cells Using JC-1. For the JC-1 monomer probes, emissions were collected at 500-600 nm (λ_ex_ = 514 nm), For the JC-1 aggregates probes, emissions were collected at 600-700 nm (λ_ex_ = 585 nm), For the DAPI probes, emissions were collected at 400-500 nm (λ_ex_ = 350 nm).


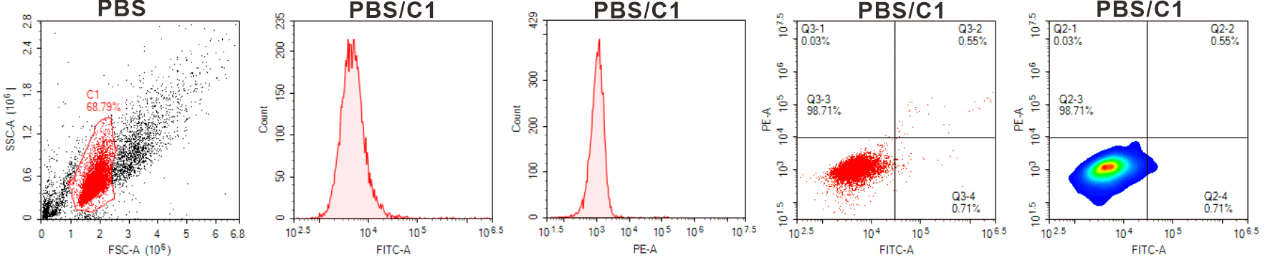


**Figure S33.** FCM apoptosis gating strategy.


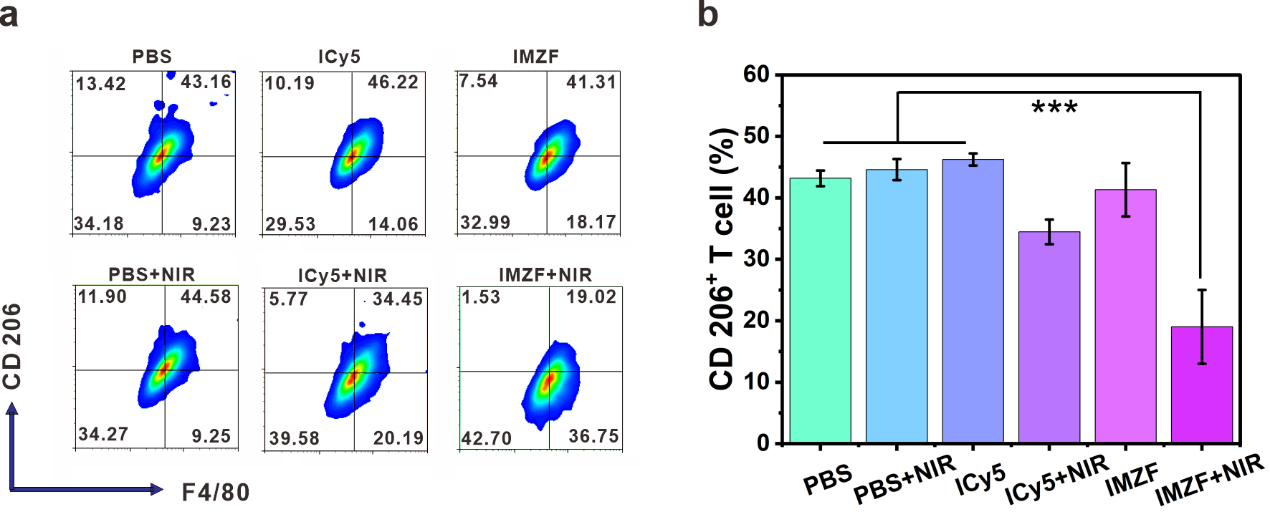


**Figure S34.** In vitro immune activated by IMZF. (a) FCM and (b) the relevant quantitative analysis of M2 phenotype macrophages (mean ± SD, n = 3, *p < 0.05 and **p < 0.01).


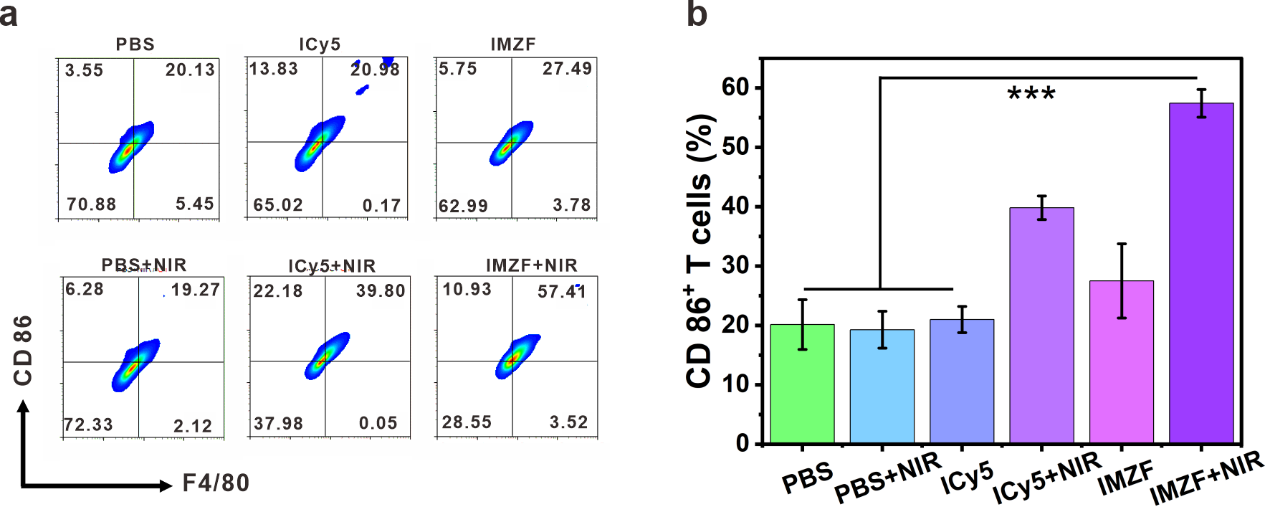


**Figure S35.** In vitro immune activated by IMZF. (a) FCM and (b) the relevant quantitative analysis of M1 phenotype macrophages (mean ± SD, n = 3, *p < 0.05 and **p < 0.01).


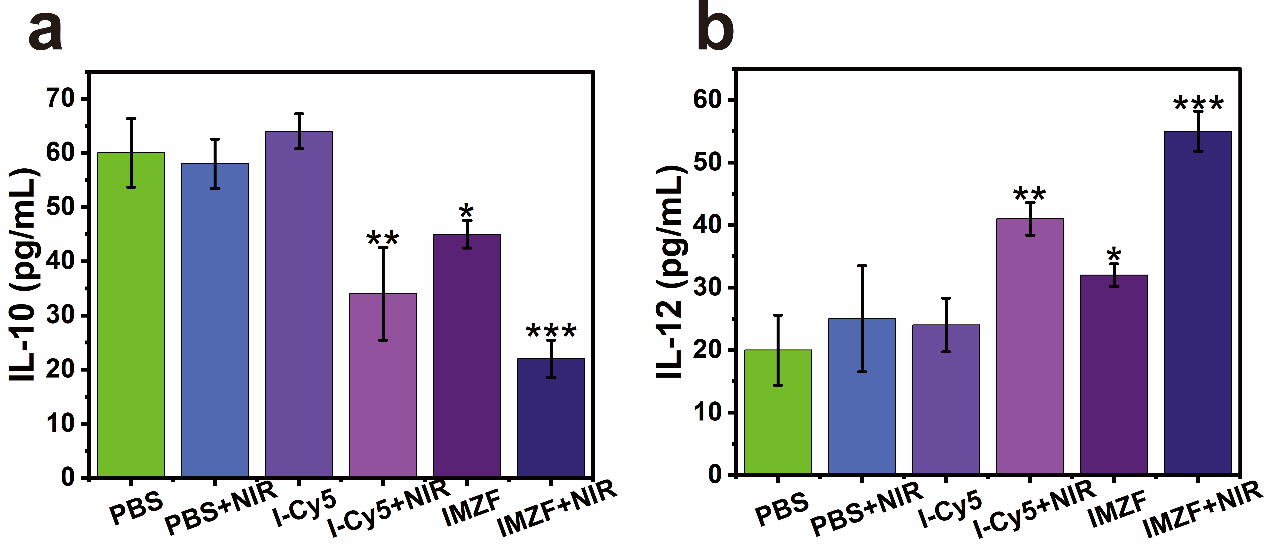


**Figure S36.** The concentration of IL-10 and IL-12 in the supernatant post various treatments (mean ± SD, n = 3, *p < 0.05, **p < 0.01, ***p < 0.001).


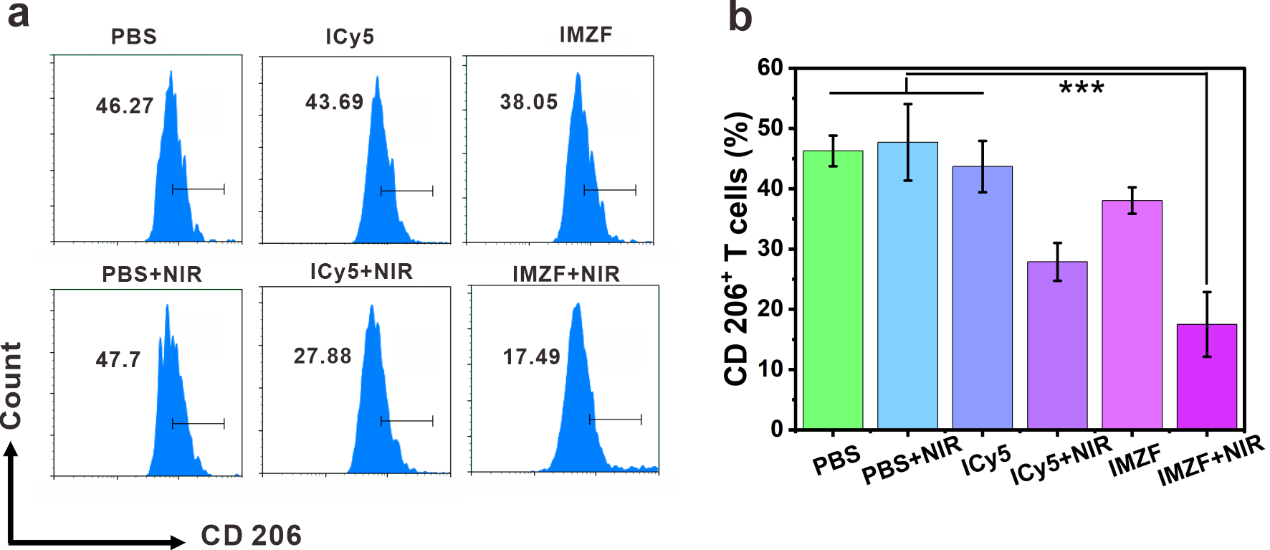


**Figure S37.** In vitro immune activated by IMZF. (a) FCM and (b) the relevant quantitative analysis of M2 phenotype macrophages (mean ± SD, n = 3, *p < 0.05 and **p < 0.01).


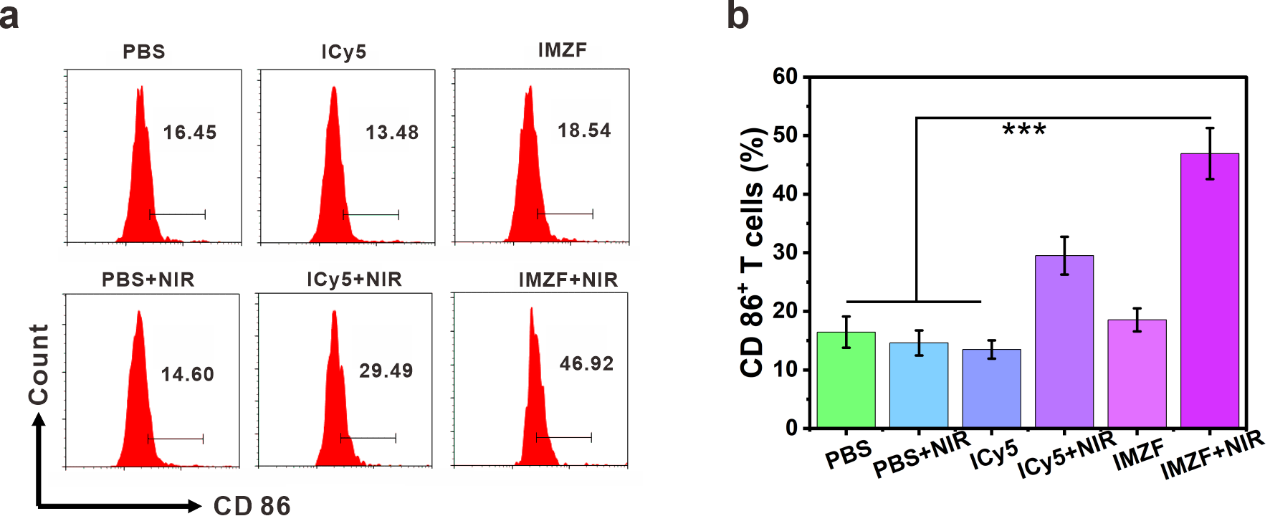


**Figure S38.** In vitro immune activated by IMZF. (a) FCM and (b) the relevant quantitative analysis of M1 phenotype macrophages (mean ± SD, n = 3, *p < 0.05 and **p < 0.01).


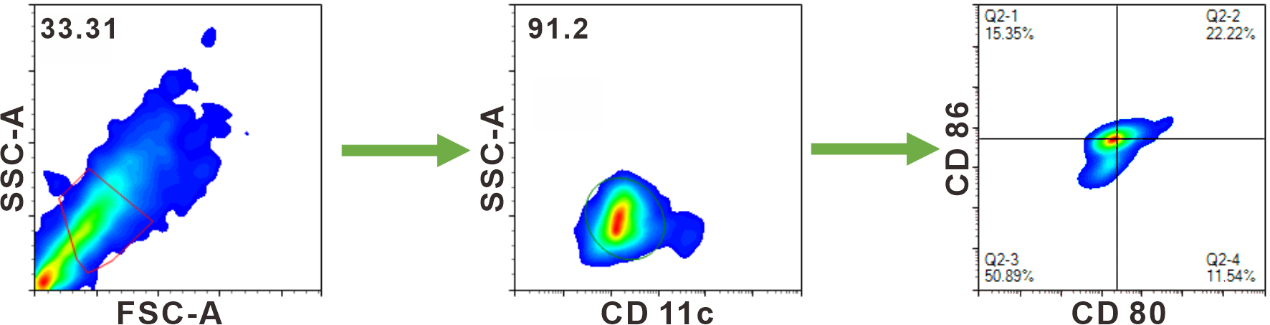


**Figure S39.** Representative FCM gating strategy for the DCs (CD11c^+^CD80^+^CD86^+^) in the tumors.


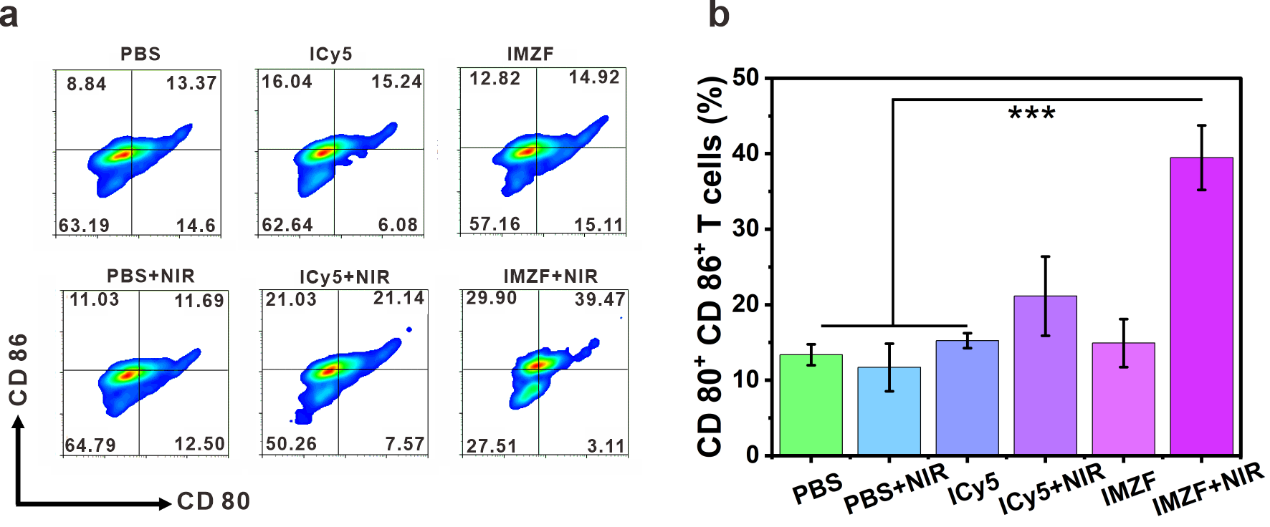


**Figure S40.** Flow cytometric detection of CD80^+^ CD86^+^ on the surface of mouse tumors (distant tumor).


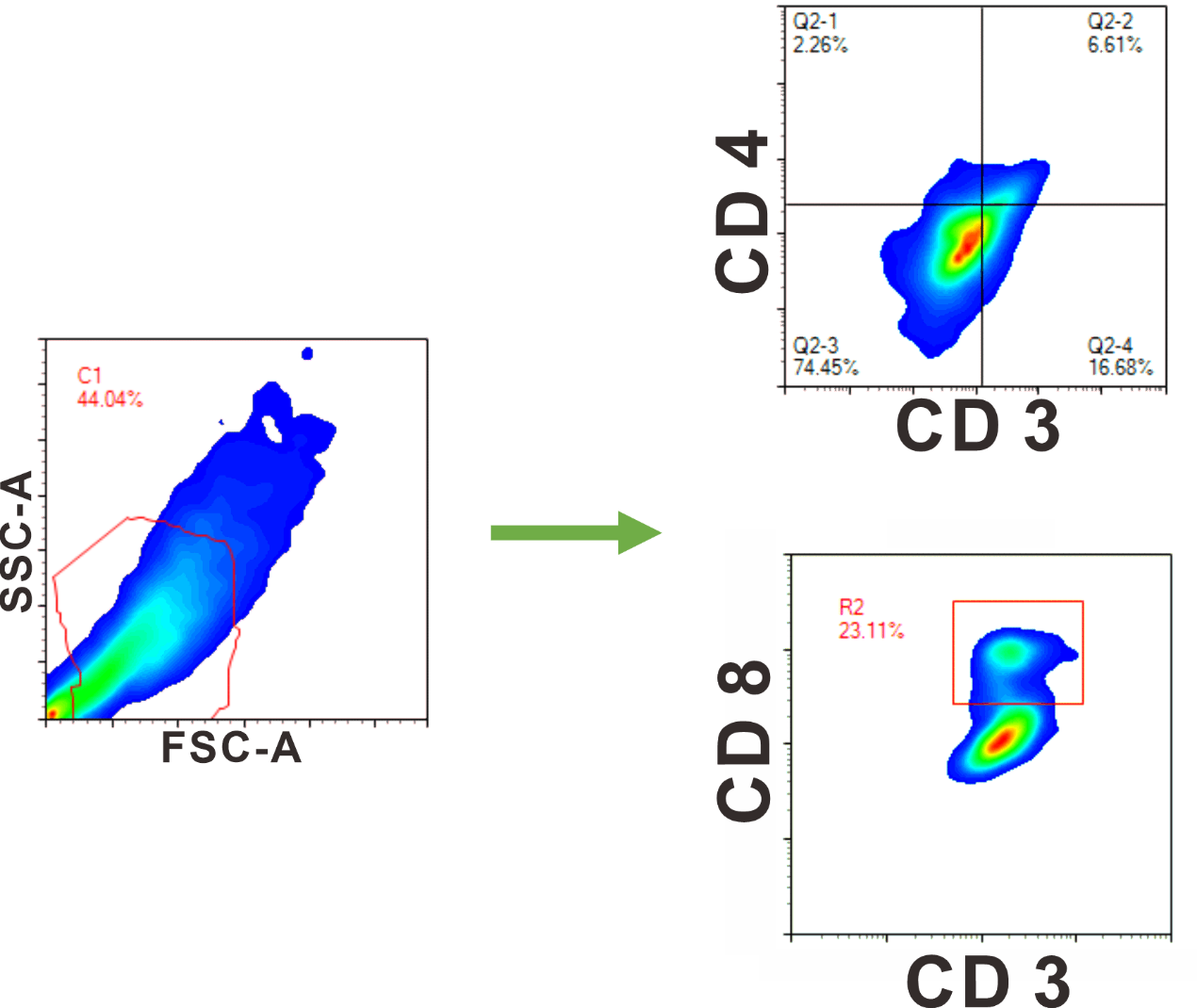


**Figure S41.** Representative flow cytometry gating strategies for CD3^+^CD4^+^ and CD3^+^CD8^+^ T cells in the tumors.


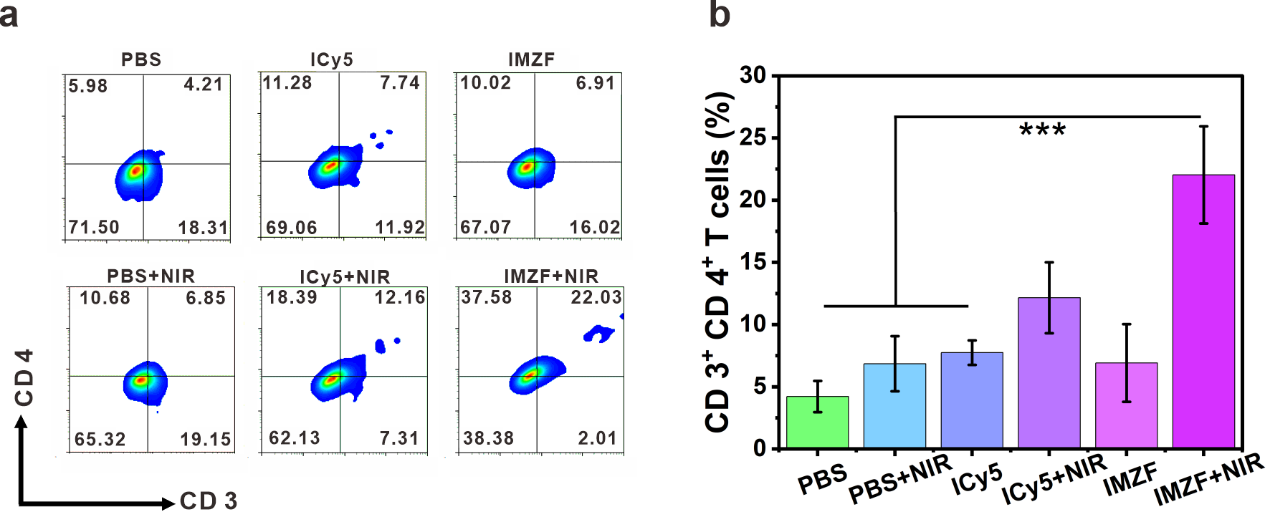


**Figure S42.** Flow cytometric detection of CD3^+^ CD4^+^ on the surface of mouse tumors (distant tumor).


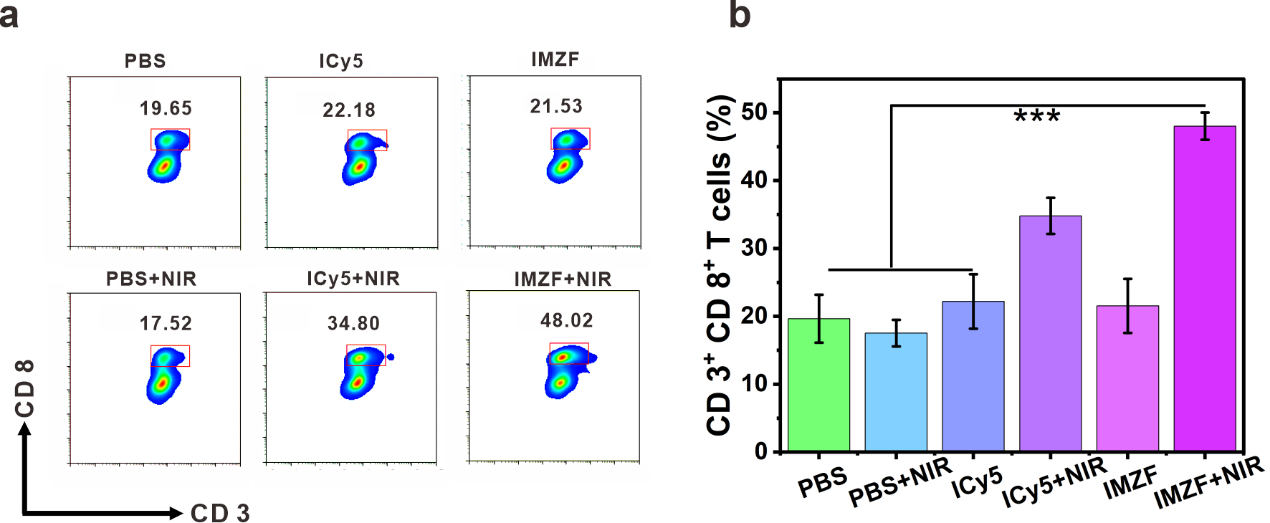


**Figure S43.** Flow cytometric detection of CD3^+^ CD8^+^ on the surface of mouse tumors (distant tumor).


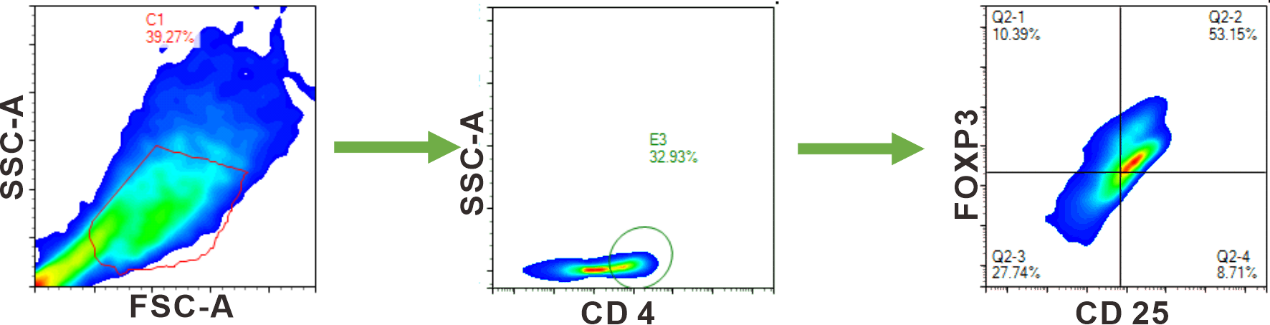


**Figure S44.** Representative FCM gating strategy for the Treg(CD4^+^CD25^+^FoxP3^+^) in the tumors.


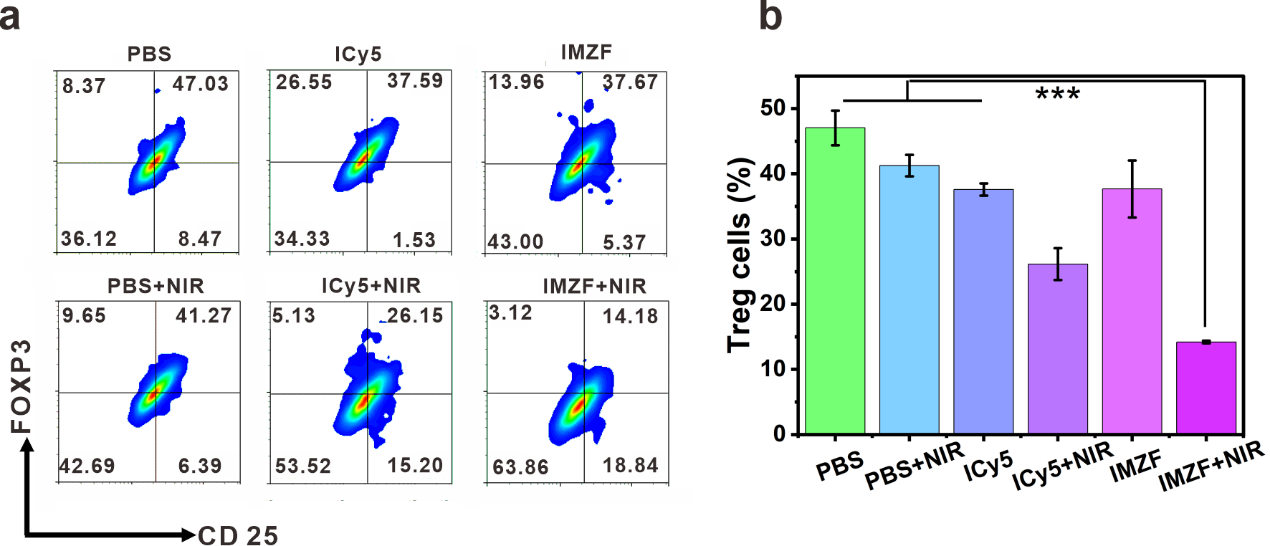


**Figure S45.** Flow cytometric detection of CD25^+^ FOXP3^+^ on the surface of mouse tumors (distant tumor).


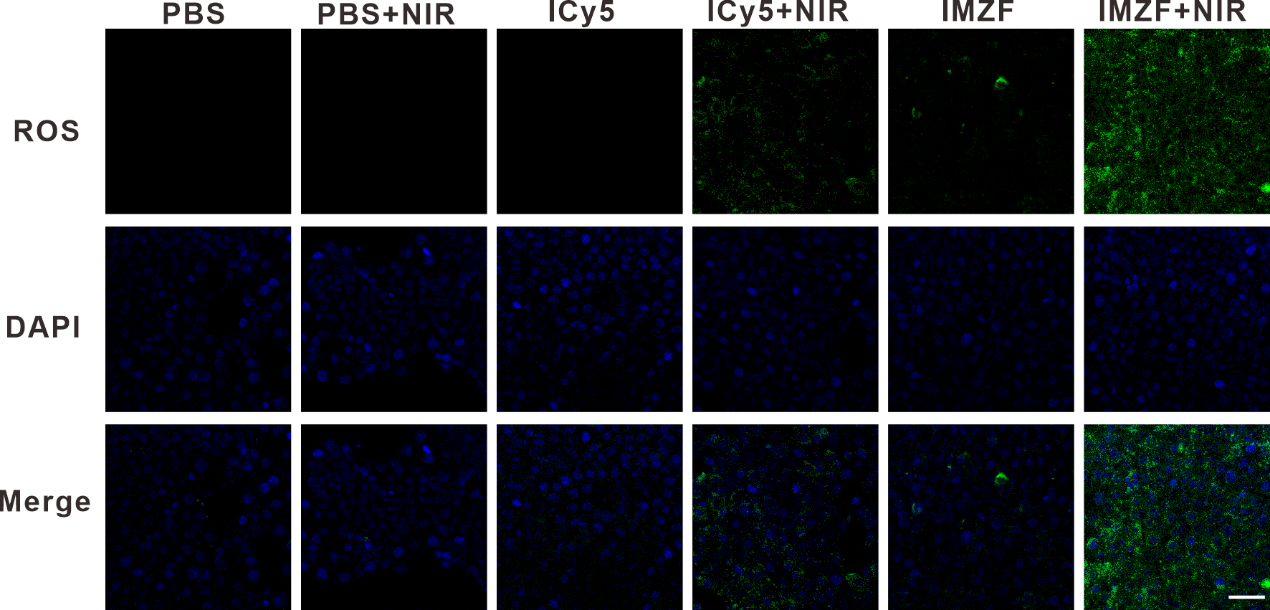


**Figure S46.** Detection of ROS in mouse tumor tissues.


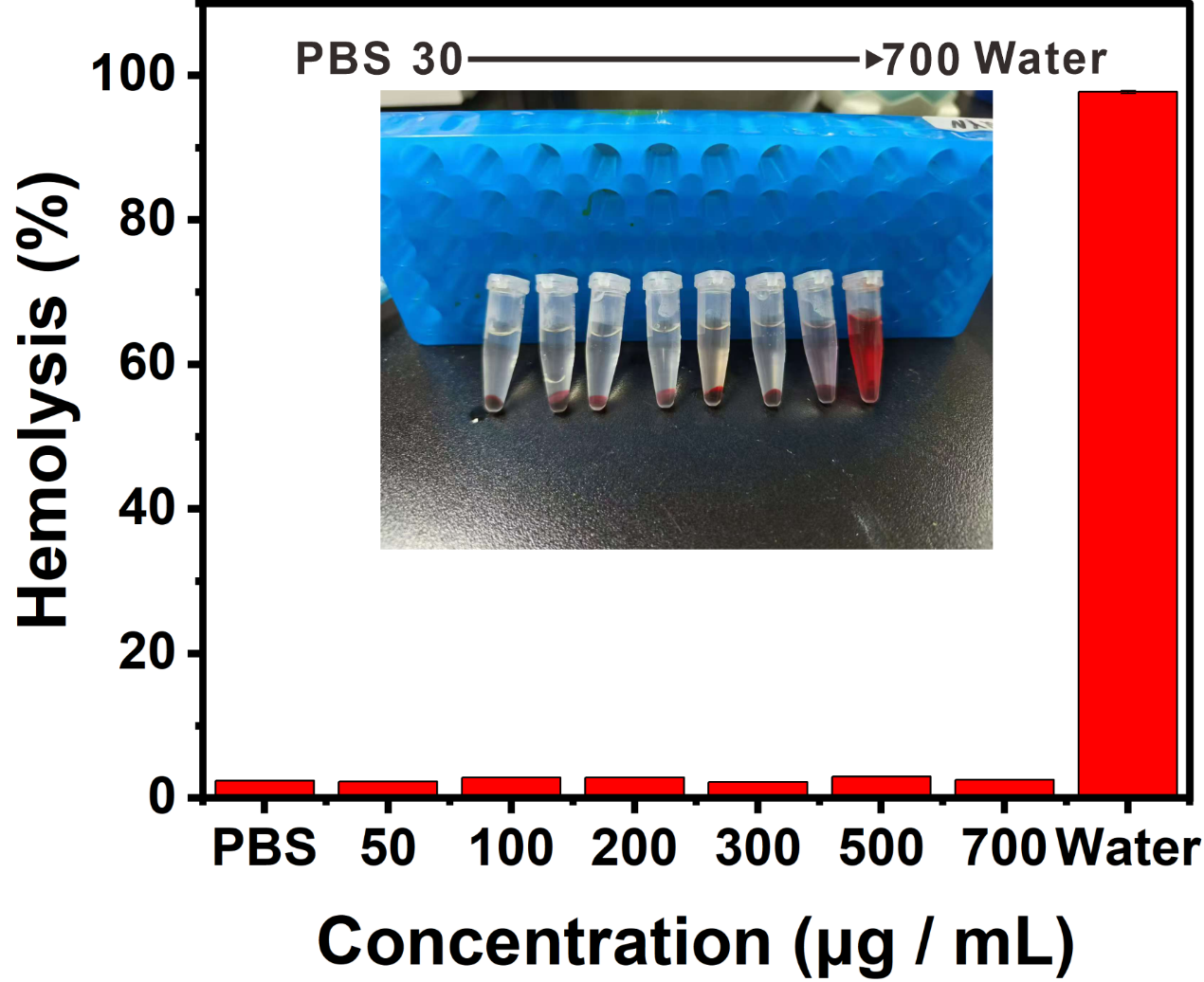


**Figure S47.** Determine the in vitro hemolysis rate of different concentrations of IMZF incubated with mouse red blood cells at 37°C for 4 h. PBS serves as the negative control, and deionized water as the positive control (mean ± SD, n = 3).


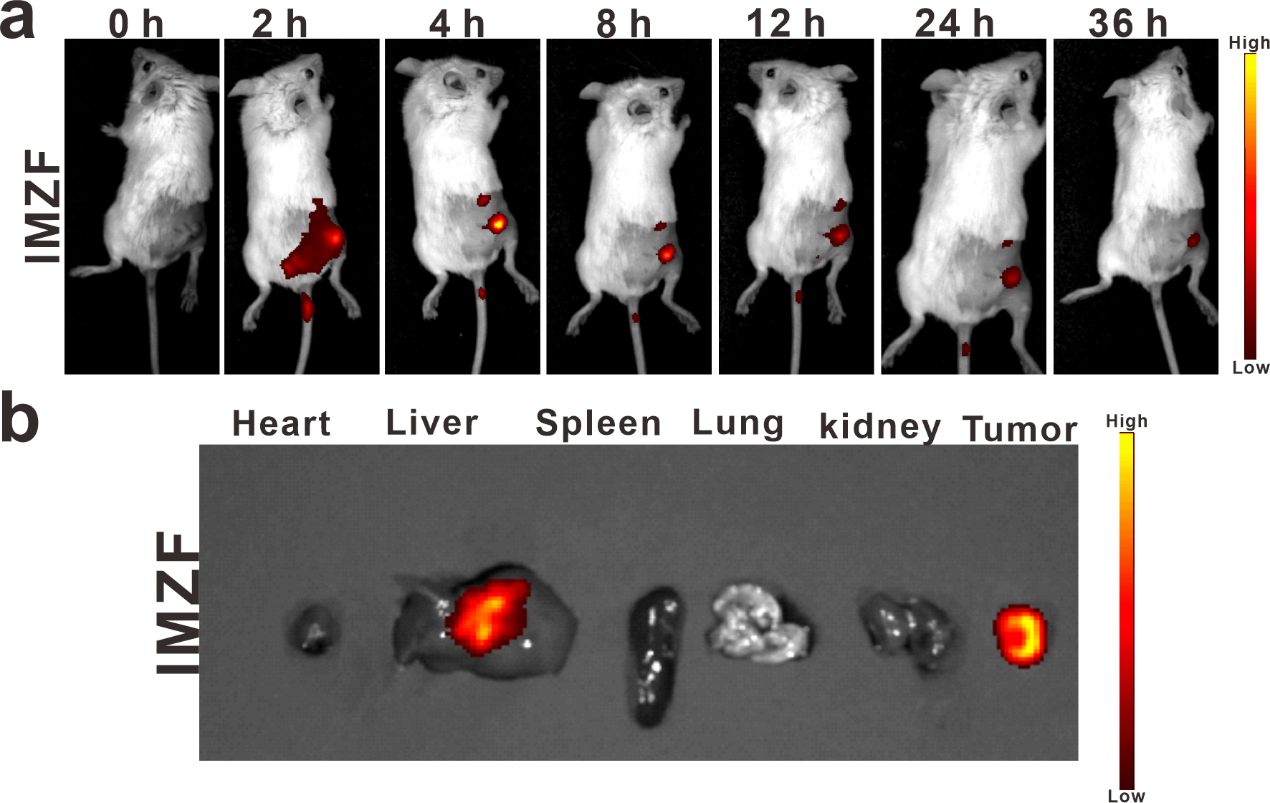


**Figure S48.** (a)Time-dependent fluorescence imaging of subcutaneous 4T1 tumor-bearing mice after i.v. injection of IMZF. (b) Fluorescence imaging of isolated organs in IMZF groups at 36 h post-injection (mean ± SD, n = 3).


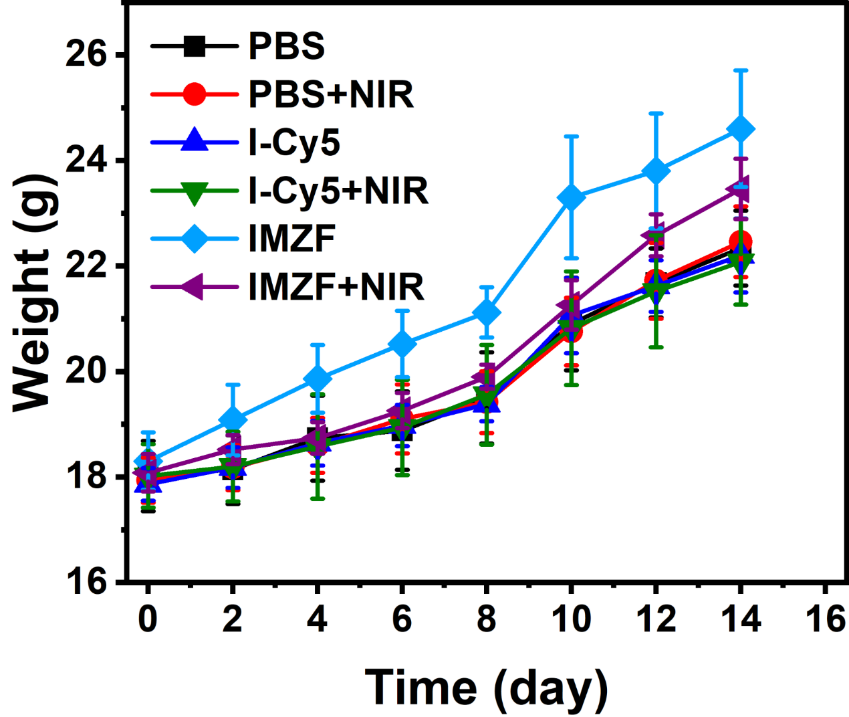


**Figure S49.** Mouse body weight.


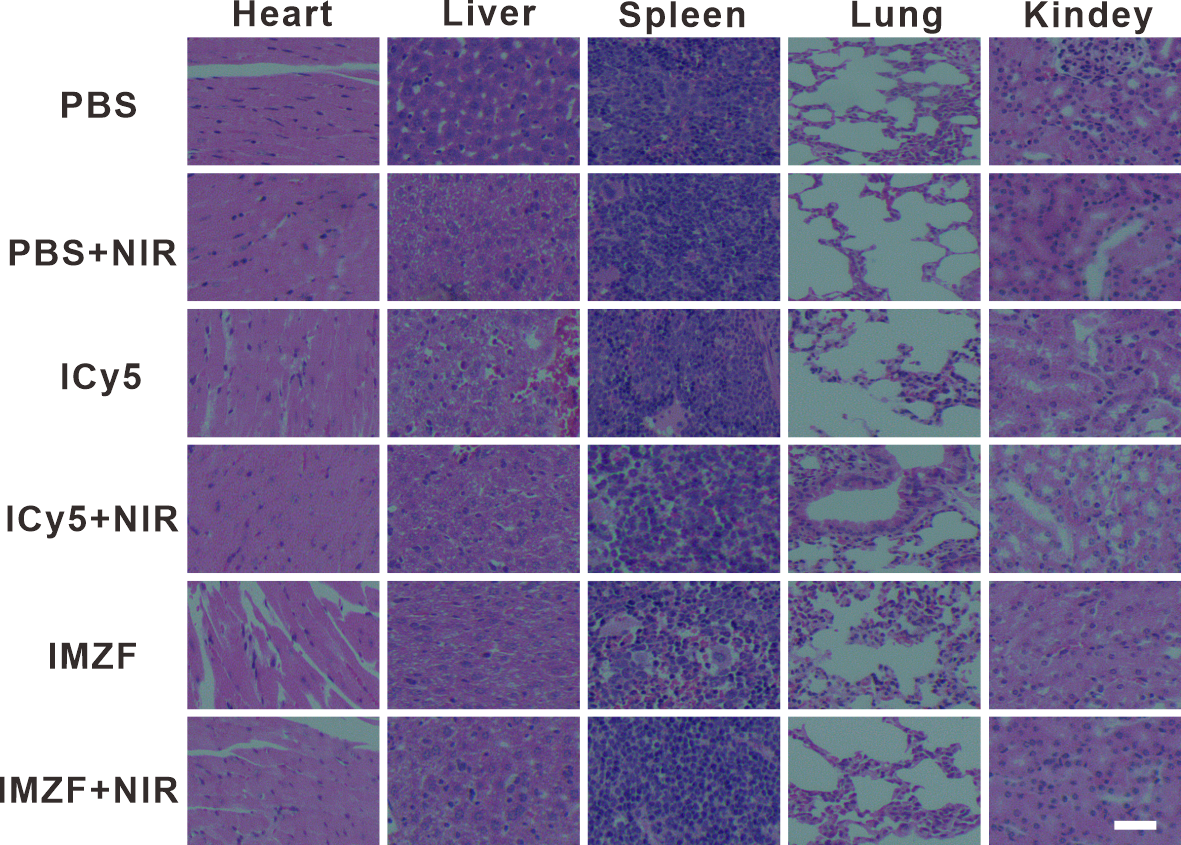


**Figure S50.** Histological Staining Experiments on Mouse Heart, Liver, Spleen, Lung, and Kidney Tissues.


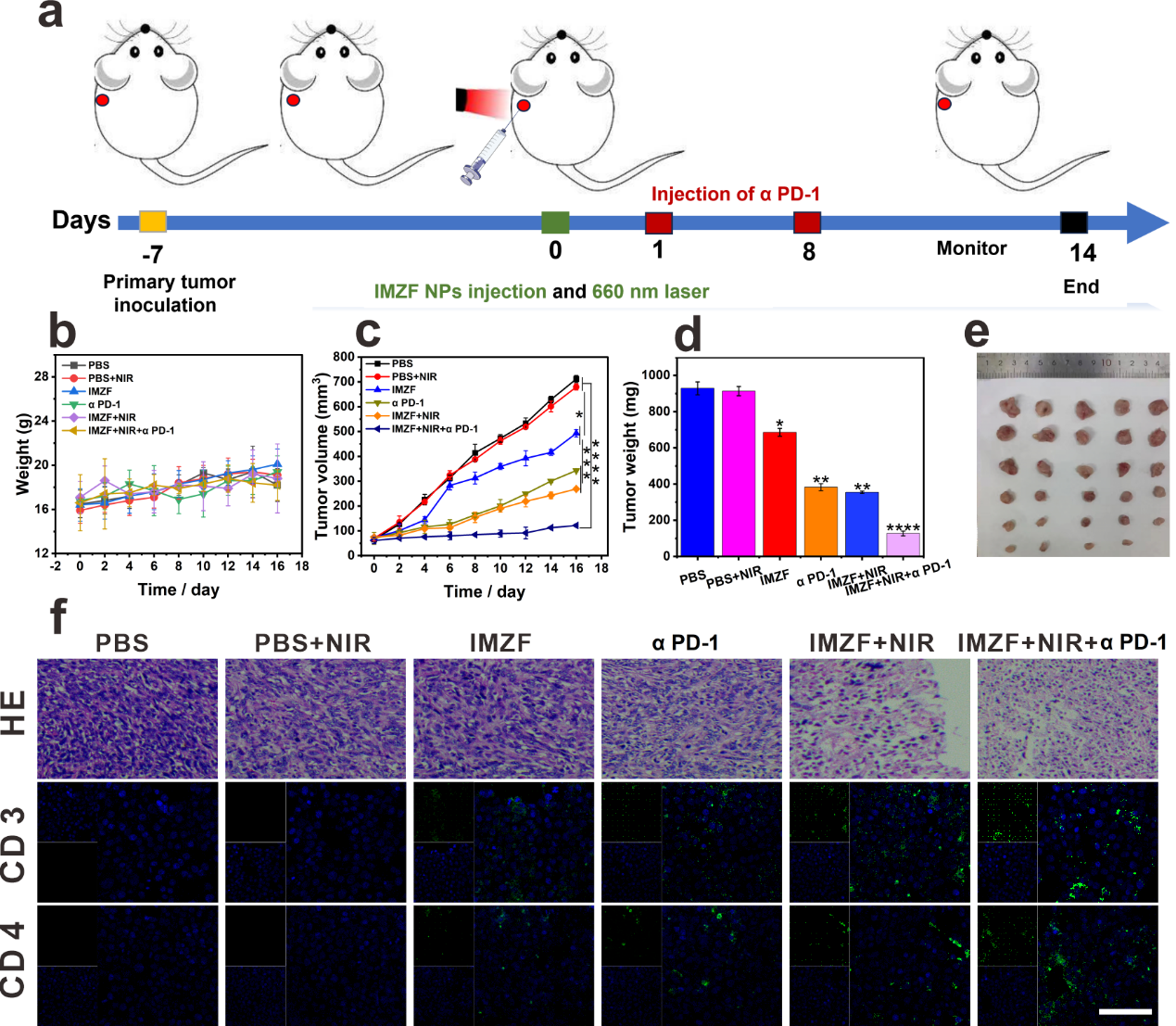


**Figure S51.** (a) Mouse model generation process diagram. (b) The weight of tumors in mice (mean ± SD, n = 5). (c) The volume of tumors in mice (mean ± SD, n = 5). (d) The weight of tumors in mice (mean ± SD, n = 5). (e) Images of proximal tumors in mice. (f) Immunofluorescent staining of tumor tissues in mice, including CD3 molecule, CD4 molecule.


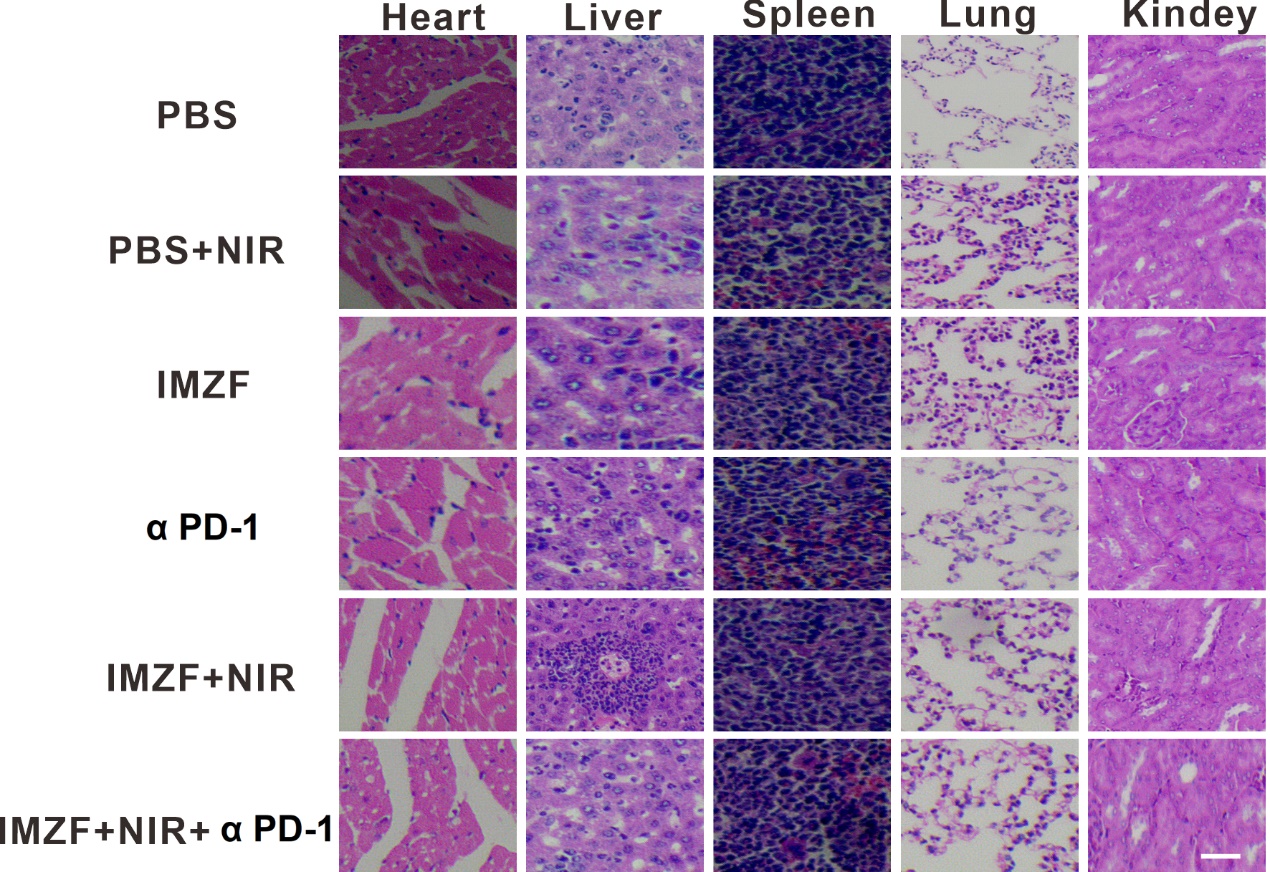


**Figure S52.** Histological Staining Experiments on Mouse Heart, Liver, Spleen, Lung, and Kidney Tissues.


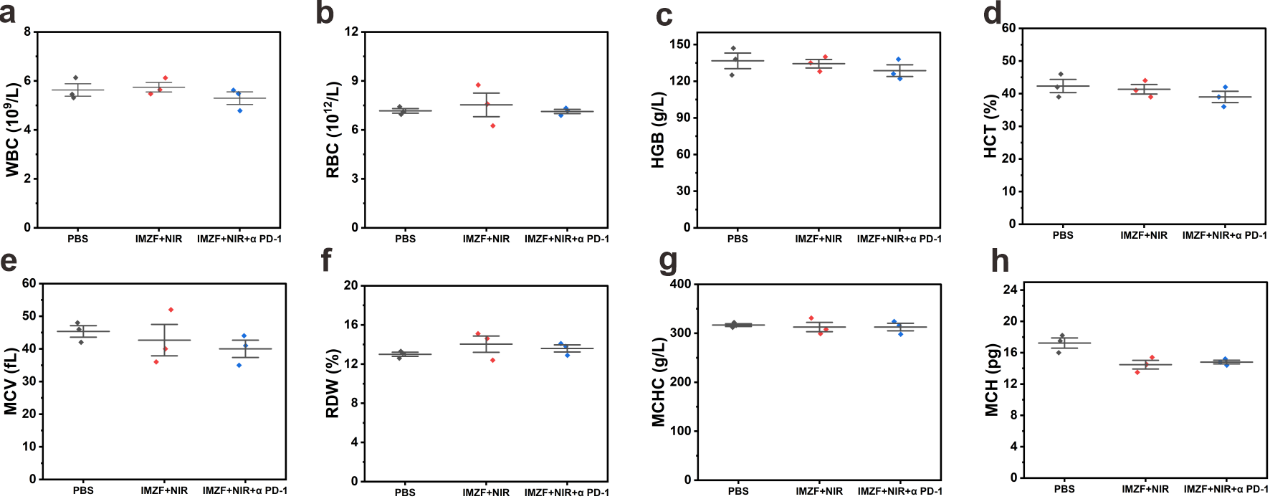


**Figure S53.** The routine blood analysis in different treatment groups. a) White blood cells (WBC), b) red blood cells (RBC), c) concentration of hemoglobin (HGB), d) hematocrit (HCT), e) mean corpuscular volume (MCV), f) red blood cell volume distribution width (RDW), g) mean corpuscular hemoglobin concentration (MCHC), h) mean corpuscular hemoglobin (MCH). (mean ± SD, n = 3).


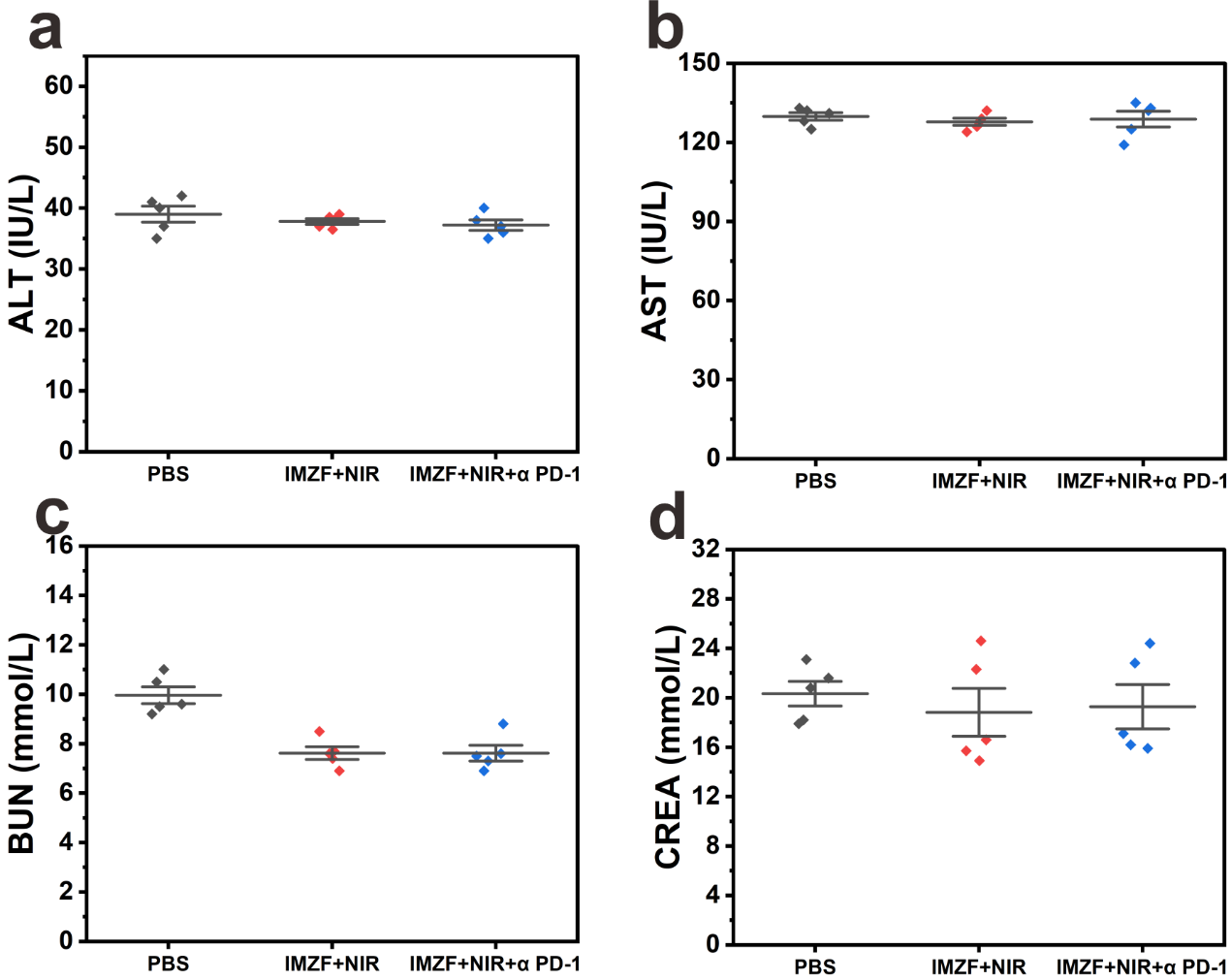


**Figure S54.** Blood biochemistry indexes including a) alanine transaminase (ALT), b) aspartate aminotransferase (AST), c) blood urea nitrogen (BUN), and d) creatinine (CREA) in mice with different treatments. (mean ± SD, n = 5).
